# Safety and efficacy of meplazumab in healthy volunteers and COVID-19 patients: a randomized phase 1 and an exploratory phase 2 trial

**DOI:** 10.1038/s41392-021-00603-6

**Published:** 2021-05-17

**Authors:** Huijie Bian, Zhao-Hui Zheng, Ding Wei, Aidong Wen, Zheng Zhang, Jian-Qi Lian, Wen-Zhen Kang, Chun-Qiu Hao, Jing Wang, Rong-Hua Xie, Ke Dong, Jie-Lai Xia, Jin-Lin Miao, Wen Kang, Guoquan Li, Di Zhang, Mingru Zhang, Xiu-Xuan Sun, Likun Ding, Kui Zhang, Junfeng Jia, Jin Ding, Zhiqin Li, Yanyan Jia, Lin-Na Liu, Zhe Zhang, Zhao-Wei Gao, Hong Du, Na Yao, Qing Wang, Ke Wang, Jie-Jie Geng, Bin Wang, Ting Guo, Ruo Chen, Yu-Meng Zhu, Li-Juan Wang, Qian He, Rui-Rui Yao, Ying Shi, Xiang-Min Yang, Jian-Sheng Zhou, Yi-Nan Ma, Ya-Tao Wang, Xue Liang, Fei Huo, Zhe Wang, Yang Zhang, Xu Yang, Ye Zhang, Lu-Hua Gao, Ling Wang, Xiao-Chun Chen, Hao Tang, Shuang-Shuang Liu, Qing-Yi Wang, Zhi-Nan Chen, Ping Zhu

**Affiliations:** 1 National Translational Science Center for Molecular Medicine and Department of Cell Biology, Fourth Military Medical University, Xi’an, China; 2 Department of Clinical Immunology, Xijing Hospital, Fourth Military Medical University, Xi’an, China; 3 Department of Pharmacy, Xijing Hospital, Fourth Military Medical University, Xi’an, China; 4 Center for Infectious Diseases, Tangdu Hospital, Fourth Military Medical University, Xi’an, China; 5 Department of Nuclear Medicine, Xijing Hospital, Fourth Military Medical University, Xi’an, China; 6 Department of Clinical Diagnosis, Tangdu Hospital, Fourth Military Medical University, Xi’an, China; 7 College of Military Preventive Medicine, Fourth Military Medical University, Xi’an, China; 8 Department of Pharmaceutics, Tangdu Hospital, Fourth Military Medical University, Xi’an, China; 9 Department of Pathology, Fourth Military Medical University, Xi’an, China; 10 Jiangsu Pacific Meinuoke Biopharmaceutical Co. Ltd, Changzhou, China; 11 Department of Foreign Languages, Fourth Military Medical University, Xi’an, China

**Keywords:** Molecular medicine, Infectious diseases

## Abstract

Recent evidence suggests that CD147 serves as a novel receptor for severe acute respiratory syndrome coronavirus 2 (SARS-CoV-2) infection. Blocking CD147 via anti-CD147 antibody could suppress the in vitro SARS-CoV-2 replication. Meplazumab is a humanized anti-CD147 IgG_2_ monoclonal antibody, which may effectively prevent SARS-CoV-2 infection in coronavirus disease 2019 (COVID-19) patients. Here, we conducted a randomized, double-blinded, placebo-controlled phase 1 trial to evaluate the safety, tolerability, and pharmacokinetics of meplazumab in healthy subjects, and an open-labeled, concurrent controlled add-on exploratory phase 2 study to determine the efficacy in COVID-19 patients. In phase 1 study, 59 subjects were enrolled and assigned to eight cohorts, and no serious treatment-emergent adverse event (TEAE) or TEAE grade ≥3 was observed. The serum and peripheral blood *C*_max_ and area under the curve showed non-linear pharmacokinetic characteristics. No obvious relation between the incidence or titer of positive anti-drug antibody and dosage was observed in each cohort. The biodistribution study indicated that meplazumab reached lung tissue and maintained >14 days stable with the lung tissue/cardiac blood–pool ratio ranging from 0.41 to 0.32. In the exploratory phase 2 study, 17 COVID-19 patients were enrolled, and 11 hospitalized patients were involved as concurrent control. The meplazumab treatment significantly improved the discharged (*P* = 0.005) and case severity (*P* = 0.021), and reduced the time to virus negative (*P* = 0.045) in comparison to the control group. These results show a sound safety and tolerance of meplazumab in healthy volunteers and suggest that meplazumab could accelerate the recovery of patients from COVID-19 pneumonia with a favorable safety profile.

## Introduction

The coronavirus disease 2019 (COVID-19) pandemic caused by severe acute respiratory syndrome coronavirus 2 (SARS-CoV-2) is considered the greatest challenge we have faced since World War II and has spread to every continent in 2020.^1^ The infection of SARS-CoV-2 leads to acute viral exudative pneumonia, presenting bilateral diffuse alveolar damage with cellular fibromyxoid exudates.^2^ About 20% of the patients have developed severe pneumonia and acute respiratory distress syndrome, which contribute to death.^3^ The COVID-19 has caused more than 100,000,000 detected cases and claimed >2,000,000 lives worldwide as of January 27, 2021.^4^

The current standard care of COVID-19 includes symptomatic treatment, oxygen therapy, antiviral treatment, fluid management, and antimicrobial therapy for secondary bacterial infections.^5,6^ Although neutralizing antibody drugs, bamlanivimab and REGEN-COV (casirivimab and imdevimab cocktail), have been authorized for emergency use in the treatment of mild-to-moderate COVID-19 out-patients, specific drugs with favorable efficacy are still in need to constrain the severe and critical cases.^7,8^

Angiotensin-converting enzyme 2 (ACE2) has been reported as the host cellular receptor for SARS-CoV-2,^9^ which exists in various human tissues.^10^ Our recent study reveals a novel virus entry route through which CD147 binds with SARS-CoV-2 spike protein, facilitating virus infection to host cells. Furthermore, CD147 is identified as a novel receptor for SARS-CoV-2 infection in target cells, including T lymphocytes.^11^ These results verify that CD147 is a central target for the development of a specific and effective drug against COVID-19. We previously generated a humanized anti-CD147 IgG_2_ monoclonal antibody, meplazumab (also known as HP6H8), which is proved to abolish the parasite invasion by blocking the interaction between CD147 on erythrocytes and rhoptry-associated protein two on *Plasmodium falciparum*.^12,13^ Meplazumab was approved for phase 1 trials by the US Food and Drug Administration (FDA) in the treatment and prophylaxis of severe malaria in January 2020 (IND 143872, Fast Track). We also prove that meplazumab can block the SARS-CoV-2-induced cytopathic effect dose-dependently with 24.86 μg/mL half-maximal effective concentration (EC_50_), and suppress the virus titer with 15.16 μg/mL half-maximal inhibitory concentration (IC_50_), which indicates that meplazumab significantly inhibits SARS-CoV-2 infection in vitro.^11^ Meplazumab has been approved for phase 1/2 trials in treating COVID-19 by the National Medical Products Administration of China (No. 2020L00012) on March 28, 2020. After finishing the phase 1 study, we applied for the Investigation New Drug from FDA, and it was approved for phase 2/3 trials on November 13, 2020 (IND 149626). In this article, we report the safety, tolerance, and pharmacokinetics (PK) results from a phase 1 clinical trial in healthy individuals, and the efficacy from an exploratory phase 2 in COVID-19 patients.

## Results

### Study participants and safety analyses of phase 1 trial

**Fig. 1:**
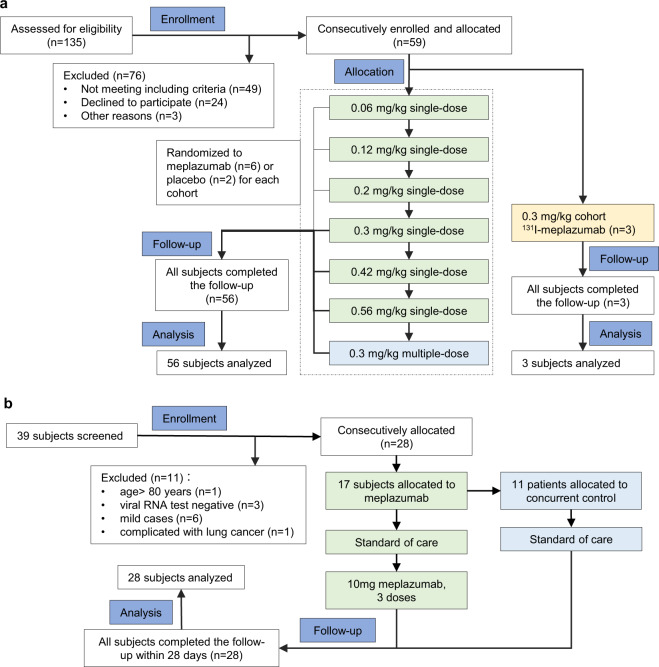
Flow chart of the phase 1 trial (**a**) and exploratory phase 2 trial (**b**)

The phase 1 study is a single-centered, randomized, double-blinded, placebo-controlled trial to evaluate the safety, tolerability, PK, and biodistribution in healthy volunteers. Between April 22, 2020 and May 29, 2020, 59 out of 135 healthy volunteers were enrolled and received the corresponding doses. Forty-eight subjects were assigned to six single-dose cohorts successively. After the last dose (0.56 mg/kg) in the single-dose cohort, eight newly recruited subjects were assigned to the multiple dose, and three new subjects were assigned to the biodistribution study cohort. At data cutoff (August 18, 2020), enrollment of all planned cohorts and planned visits for all subjects were completed (Fig. 1a). The demographic data are summarized in Table 1.

**Table 1:** Summary of demographics characteristics in phase 1 study

Parameter statistics	Single dose							Multiple dose	
	0.06 mg/kg (*n* = 6), *n* (%)	0.12 mg/kg (*n* = 6), *n* (%)	0.2 mg/kg (*n* = 6), *n* (%)	0.3 mg/kg (*n* = 6), *n* (%)	0.42 mg/kg (*n* = 6), *n* (%)	0.56 mg/kg (*n* = 6), *n* (%)	Placebo (*n* = 12), *n* (%)	0.3 mg/kg (*n* = 6), *n* (%)	Placebo (*n* = 2), *n* (%)
Age (years)									
Mean (SD)	30.5 (6.83)	25.2 (5.42)	30.2 (7.88)	39.5 (8.46)	28.8 (9.47)	30.7 (10.58)	29.4 (6.47)	34.0 (10.60)	26.5 (4.95)
Min–max	23–42	18–32	20–43	28–49	20–47	18–44	18–38	24–48	23–30
Gender, *n* (%)									
Male	5 (83.3)	6 (100)	5 (83.3)	5 (83.3)	4 (66.7)	6(100)	11 (91.7)	4 (66.7)	2 (100)
Female	1 (16.7)	0	1 (16.7)	1 (16.7)	2 (33.3)	0	1 (8.3)	2 (33.3)	0
Height (m)									
Mean (SD)	1.724 (0.107)	1.723 (0.044)	1.687 (0.075)	1.737 (0.059)	1.683 (0.067)	1.701 (0.083)	1.716 (0.088)	1.668 (0.081)	1.745 (0.127)
Min–max	1.595–1.890	1.680–1.780	1.575–1.760	1.650–1.815	1.580–1.750	1.560–1.795	1.515–1.850	1.540–1.780	1.655–1.835
Weight (kg)									
Mean (SD)	68.02 (11.833)	67.73 (3.894)	66.85 (8.182)	71.55 (7.546)	67.92 (5.654)	67.72 (12.231)	67.98 (8.992)	65.27 (9.088)	68.45 (2.192)
Min–max	51.4–83.3	61.2–72.5	55.2–77.7	62.0–81.9	57.4–72.8	54.0–83.9	54.2–81.9	56.3–78.0	66.9–70.0
BMI (kg/m^2^)^a^									
Mean (SD)	22.72 (2.011)	22.85 (1.439)	23.45 (1.700)	23.72 (2.122)	23.97 (1.352)	23.27 (2.590)	23.03 (1.963)	23.42 (2.084)	22.75 (4.031)
Min–max	18.9–24.6	21.1–24.4	21.0–25.4	21.2–26.0	21.9–25.5	19.5–26.0	19.8–25.7	21.1–25.9	19.9–25.6

^a^BMI (kg/m^2^) = weight (kg)/height (m)^2^.

No death, serious TEAE, or TEAE grade ≥3 was reported; no subject was withdrawn from the trial or discontinued from treatment due to the meplazumab-related TEAEs. The incidence rate of study medication-related TEAEs ranged from 25.0 to 87.5% across dose cohorts, with no apparent dose-dependency reported (Table 2). All TEAEs in this study were grade 1, except one subject reported grade 2 pyrexia. All TEAEs were resolved without medical intervention, and no related clinical signs or symptoms (except pyrexia) were observed. The TEAEs with the highest incidence in the single-dose study were blood bilirubin increase and blood-unconjugated bilirubin increase, with incidence rate ranging from 16.7 to 66.7% in a dose-independent manner. The TEAEs with the highest incidence in the multiple-dose study were granulocyte percentage increase, granulocyte count increase, and lymphocyte percentage decrease, with an incidence rate of 83.3%. The summary of meplazumab-related TEAEs by system organ class and preferred term are tabulated in Supplementary Table 1. No concomitant medication was administered in this study.

**Table 2:** Summary of treatment-emergent adverse events in phase 1 study

	Single-dose							Multiple-dose	
	0.06 mg/kg (*n* = 6), *n* (%), *m*	0.12 mg/kg (*n* = 6), *n* (%), *m*	0.2 mg/kg (*n* = 6), *n* (%), *m*	0.3 mg/kg (*n* = 6), *n* (%), *m*	0.42 mg/kg (*n* = 6), *n* (%), *m*	0.56 mg/kg (*n* = 6), *n* (%), *m*	Placebo (*n* = 12), *n* (%), *m*	0.3 mg/kg (*n* = 6), *n* (%), *m*	Placebo (*n* = 2), *n* (%), *m*
TEAEs	1 (16.7), 4	6 (100), 44	3 (50.0), 12	6 (100), 35	3 (50.0), 16	6 (100), 57	6 (50.0), 12	5 (83.3), 58	0
TEAEs with CTCAE grade ≥3	0	0	0	0	0	0	0	0	0
Serious TEAEs	0	0	0	0	0	0	0	0	0
Study medication-related TEAEs	1 (16.7), 3	6 (100), 43	2 (33.3), 6	6 (100), 33	2 (33.3), 12	5 (83.3), 54	5 (41.7), 11	5 (83.3), 58	0
Study medication-related serious TEAEs	0	0	0	0	0	0	0	0	0
TEAEs leading to study medication dose adjustment or interruption	0	0	0	0	0	0	0	0	0
TEAEs leading to withdrawal study	0	0	0	0	0	0	0	0	0
TEAEs leading to death	0	0	0	0	0	0	0	0	0
Injection site reactions	0	0	0	0	0	0	0	0	0

*N* the number of safety analysis set subjects in each group, *n* (%) the number and percentages of subjects with at least one TEAE, *m* number of events.

### Meplazumab immunogenicity

Anti-drug antibody (ADA) was observed in all cohorts of this study, including two subjects administered a placebo. From predose to Day 84, the ADA incidence of 0.06, 0.12, 0.2, 0.3, 0.42, and 0.56 mg/kg for single dose, and 0.3 mg/kg for multiple dose was 3/6, 4/6, 3/5, 1/6, 4/6, 3/6, and 5/6, respectively. The maximal antibody titers were 1:32, 1:128, 1:512, 1:4, 1:128, 1:2048, and 1:64, respectively. Frequency counts by assessment day and observed range of titers are summarized in Supplementary Table 2. No obvious relation was found between the frequency of a confirmed positive ADA response (or titer) and the meplazumab dose level administered. No apparent effect of ADA formation on meplazumab blood cell concentrations and safety was observed in each cohort.

### Meplazumab PK

Meplazumab concentration in serum was low in single-dose cohorts and fell below quantifiable levels (1 ng/mL) generally within 12 h post dose. Median serum meplazumab half-life (*T*_1/2_) was estimated from the highest two single doses, ranging from 1.21 to 2.54 h. Maximum concentration (*C*_max_) and total exposure (area under the curve (AUC)) in serum showed dosage of non-linear characteristics ranging from 0.06 to 0.56 mg/kg. At doses >0.2 mg/kg, exposure showed an increase greater than a linear dose-dependent pattern. At a dosage of 0.3, 0.42, and 0.56 mg/kg, the *C*_max_ was 20.62 ± 11.05, 51.56 ± 20.70, and 26.42 ± 4.23 ng/mL, with the highest mean *C*_max_ observed in 0.42 mg/kg cohort; AUC_last_ was 12.05 ± 6.48, 30.89 ± 11.31, and 22.55 ± 7.01 h·ng/mL, respectively. The accumulation factor (AUC_(0–last)_ second dose/AUC_(0–last)_ first dose) of meplazumab in healthy subjects receiving multiple doses was 1.38, indicating no significant accumulation (Supplementary Table 3).

**Fig. 2:**
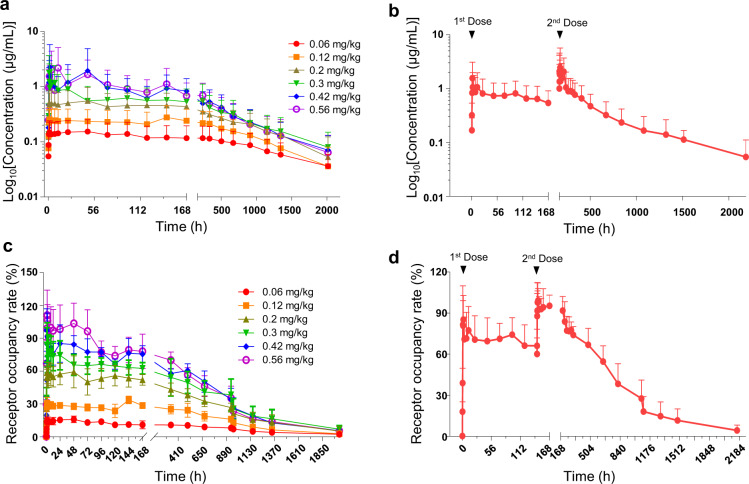
**a** Mean drug concentration–time profile on peripheral blood cells of healthy subjects in single-dose cohorts; **b** mean drug concentration–time profile on peripheral blood cells of healthy subjects in the multiple-dose cohort; **c** mean RO%-time profile on peripheral blood cells of healthy subjects in single-dose cohorts; **d** mean RO%–time profile on peripheral blood cells of healthy subjects in the multiple-dose cohort. Means and standard deviations are presented

Meplazumab concentration on peripheral blood cells was determined using a receptor occupancy (RO%) assay. Following a single intravenous infusion of meplazumab with a dose ranging from 0.06 to 0.56 mg/kg in healthy volunteers, the concentration of meplazumab binding to blood cells was positively correlated with dosage. In contrast to serum, high and extended exposure of meplazumab was observed in peripheral blood cells. Following single-dose administration, meplazumab was quantifiable in peripheral blood cells through 84 days with an estimated mean *T*_1/2_ ranging from 612 to 976 h. *C*_max_ and AUC on human peripheral blood cells showed non-linear PK characteristics (Fig. 2a and Supplementary Table 4). Both *C*_max_ and AUC_(0–inf)_ plateaued at 0.42 mg/kg dose. At multiple doses, the accumulation factor was 1.76, indicating no significant accumulation was observed. Total AUC (sum of AUC_(0–last)_ following the first dose and AUC_(0–inf)_ following the second dose) was ~15% higher than AUC_(0–inf)_ following the single 0.3 mg/kg dose, which suggests that peripheral blood cells were saturated at the repeated dose regimen (Fig. 2b and Supplementary Table 4).

After healthy volunteers were administered meplazumab (0.06–0.56 mg/kg) in a single dose via intravenous infusion, the level of RO% on human peripheral blood cells gradually escalated in a dose-dependent manner until it got the saturation state at 0.42 mg/kg (Fig. 2c). The highest mean RO% in each cohort was 15.89%, 33.69%, 59.33%, 83.92%, 97.61%, and 111.00%, respectively. On Day 84 of administration, the average RO% in each cohort was 2.26%, 2.76%, 5.21%, 6.99%, 5.62%, and 5.41%, respectively. At meplazumab doses of ≥0.2 mg/kg, mean RO% remained >10% through 84 days of sample collection. At multiple doses, RO% in the second dose increased slightly more than in the first dose. The curve of RO% vs. time for repeated doses was similar to those for single dose. The mean RO% on Day 84 of the second dosing was 4.3% (Fig. 2d). Taken together, RO% can reach a saturated state at a single dose of 0.42 or above and multiple doses of 0.3 mg/kg, maintaining a 50% level of RO% for >14 days.

### Meplazumab biodistribution

The Single-Photon Emission Computed Tomography images of subjects were shown in Supplementary Fig. 1, and the radioactivity ratios of ROI of tissues to cardiac blood pool (T/C) were shown in Table 3. As illustrated in Supplementary Fig. 1, the liver and spleen uptake of ^131^I-meplazumab was the two tissues with high background due to the metabolic functions of the reticuloendothelial system, but gradually decreased over time. The uptake of meplazumab in the liver was higher than that of the heart during the first 1 to 216 h, but lower than the uptake of the heart after 240 h post dose. The radioactivity in the heart (representing cardiac blood pool) was 56.04 ± 4.52 at 1 h after injection and gradually decreased throughout the experiment (7.79 ± 1.77 at 336 h). The uptake in other tissues, such as the lung, kidney, and thyroid, also reduced gradually. For target tissue lung, the distribution of ^131^I-meplazumab was kept stable with a T/C ratio ranging from 0.41 to 0.32 over the 1 to 336 h post dose (Table 3), indicating that the amount of meplazumab can reach its intended target tissue and sustain for at least 14 days.

**Table 3:** The ratio of tissue to cardiac blood pool (T/C) for ^131^I-meplazumab (mean ± SD)

Time	Liver	Spleen	Lung	Kidney	Thyroid
1 h	1.44 ± 0.25	0.76 ± 0.26	0.40 ± 0.03	0.28 ± 0.06	0.29 ± 0.03
2 h	1.55 ± 0.25	0.80 ± 0.31	0.41 ± 0.04	0.28 ± 0.05	0.28 ± 0.03
8 h	1.67 ± 0.22	0.79 ± 0.33	0.41 ± 0.04	0.30 ± 0.05	0.28 ± 0.04
12 h	1.69 ± 0.31	0.78 ± 0.32	0.39 ± 0.04	0.29 ± 0.06	0.28 ± 0.01
24 h	1.69 ± 0.22	0.82 ± 0.37	0.40 ± 0.06	0.31 ± 0.06	0.29 ± 0.01
36 h	1.62 ± 0.23	0.69 ± 0.21	0.38 ± 0.05	0.31 ± 0.04	0.29 ± 0.01
48 h	1.58 ± 0.23	0.67 ± 0.19	0.37 ± 0.04	0.30 ± 0.02	0.28 ± 0.02
60 h	1.54 ± 0.25	0.66 ± 0.2	0.36 ± 0.03	0.30 ± 0.03	0.29 ± 0.01
72 h	1.50 ± 0.23	0.62 ± 0.18	0.35 ± 0.03	0.30 ± 0.03	0.28 ± 0.01
84 h	1.46 ± 0.24	0.64 ± 0.16	0.36 ± 0.02	0.31 ± 0.03	0.28 ± 0.01
96 h	1.40 ± 0.23	0.61 ± 0.16	0.35 ± 0.02	0.30 ± 0.04	0.27 ± 0.01
120 h	0.79 ± 0.56	0.45 ± 0.36	0.33 ± 0.02	0.19 ± 0.13	0.18 ± 0.12
144 h	1.28 ± 0.19	0.63 ± 0.20	0.36 ± 0.03	0.32 ± 0.02	0.30 ± 0.03
168 h	1.19 ± 0.20	0.60 ± 0.17	0.33 ± 0.03	0.31 ± 0.03	0.31 ± 0.03
192 h	1.09 ± 0.17	0.60 ± 0.18	0.32 ± 0.02	0.29 ± 0.01	0.27 ± 0.01
216 h	1.03 ± 0.14	0.57 ± 0.18	0.33 ± 0.01	0.30 ± 0.02	0.27 ± 0.01
240 h	1.00 ± 0.15	0.56 ± 0.22	0.33 ± 0	0.30 ± 0.01	0.26 ± 0.02
288 h	0.92 ± 0.14	0.56 ± 0.18	0.33 ± 0	0.31 ± 0.01	0.29 ± 0.04
336 h	0.80 ± 0.09	0.55 ± 0.18	0.32 ± 0.04	0.29 ± 0.01	0.27 ± 0.04

### Study participants of exploratory phase 2 trial

To further verify the efficacy of meplazumab treatment in patients with COVID-19, we conducted an open-labeled, concurrent controlled add-on exploratory phase 2 clinical trial of meplazumab among the hospitalized COVID-19 patients. In this exploratory phase 2 trial, 39 patients diagnosed with COVID-19 were enrolled, with 17 patients assigned to the meplazumab cohort. Eleven hospitalized patients served as concurrent control. The demographic data were generally balanced across two groups, as shown in Supplementary Table 5. All patients received standard of care according to the *Chinese Clinical Guidance for COVID-19 Pneumonia Diagnosis and Treatments*,^14^ and the patients in meplazumab cohort had received 10 mg meplazumab on Days 1, 2, and 5 by intravenous infusion (Fig. 1b).

### Safety and effects analysis of meplazumab on the COVID-19 patients

In the exploratory phase 2 trial, both alanine aminotransferase (ALT) and aspartate aminotransferase (AST) elevation (≥2 upper limits of the normal range) was reported in two patients of the meplazumab group (2/17, 11.8%). In the control group, both ALT and AST elevations were reported in one case (9.1%), and a single ALT elevation was reported in one case (9.1%). The increased ALT and AST levels returned to normal after 7 days in all four patients, and the treatment procedure was not affected by their fluctuation. The abnormal transaminase was not related to meplazumab treatment after comprehensive judgment by the investigators. No other adverse event was reported in meplazumab-treated patients.

**Fig. 3:**
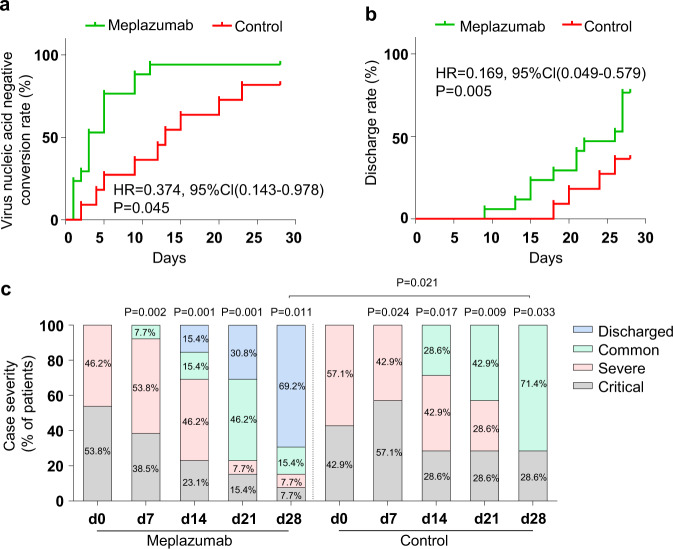
**a** Time to virus-negative conversion. The *P* value was calculated by Cox regression analysis, and *P* value 0.045 and HR 0.374, 95% CI [0.143–0.978]). The median of time to negative was 3 days (95% CI [1.5–4.5]) for the meplazumab cohort and 13 days (95% CI [6.5–19.5]) for the control cohort. **b** Analysis of time to discharge. The *P* value was determined using Cox regression analysis. **c** Distribution of case severity in severe and critical patients. The *P* values were from Ordinal regression. The *P* value comparing between baseline (Day 0) and each time point was shown on the top of the column, and the *P* value comparing between meplazumab and control at Day 28 was shown on the line

To observe the efficacy of meplazumab add-on therapy, we analyzed the time to virus negative. The result indicated that meplazumab-treated patients converted to negative in a shorter period than patients in the control group significantly (median 3, 95% confidence interval (CI) [1.5–4.5] vs. 13, [6.5–19.5]; *P* = 0.045, hazard ratio (HR) = 0.374, 95% CI [0.143–0.978], Fig. 3a). The results of multiple Cox regression indicate that no baseline characteristics, including age (*P* = 0.092), glucocorticoid treatment (*P* = 0.339), and case severity (*P* = 0.455) contributed to the difference of time to virus negative. All these data indicate an obvious favorable result of meplazumab treatment in the clearance of SARS-CoV-2.

We analyzed the time to discharge and the results indicated that meplazumab treatment significantly shortened the time to discharge when compared to the control cohort (*P* = 0.005, Fig. 3b). Multiple Cox regression results suggest that only case severity (*P* = 0.001) contributed to the difference of time to discharged. Recovery in severe and critical cases is of paramount importance to reduce the mortality of COVID-19. The case severity of severe and critical cases in both groups was improved significantly compared to baseline since 7 days post treatment (Fig. 3c). On Day 28, the case severity of the meplazumab group was markedly ameliorated compared to the control group (*P* = 0.021, Fig. 3c). These results indicated that meplazumab treatment accelerated the improvement and made a rapid recovery from COVID-19 pneumonia, especially for the severe and critical cases.

**Fig. 4:**
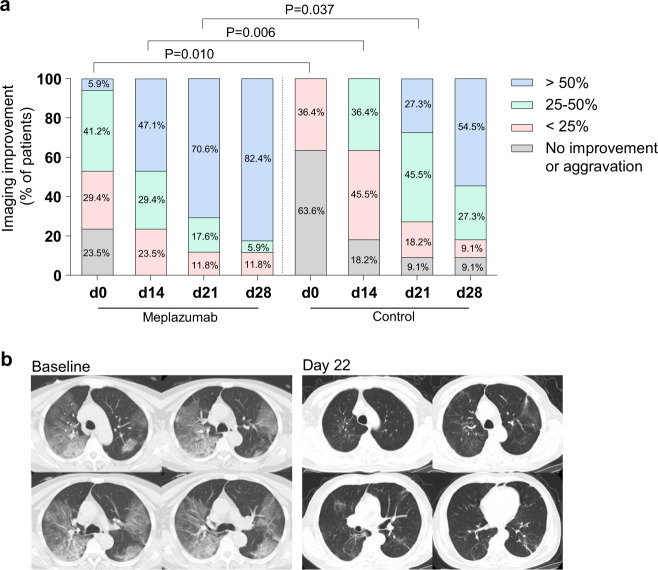
**a** Chest imaging analysis. The *P* values comparing meplazumab and control at Days 7, 14, 21, and 28 were from Ordinal regression; **b** chest CT image, transverse chest CT images from a 65-year-old male patient showing bilateral large flaky ground-glass shadows, and consolidation on the baseline period. On Day 22, the intrapulmonary lesions were significantly absorbed and dissipated by meplazumab add-on treatment

Chest radiographic analysis was performed independently by two radiologists and graded by the changed areas of ground-glass opacity and consolidation compared with the baseline. The results showed more significant improvement in the meplazumab cohort than the control cohort on Days 7, 14, and 21 (*P* = 0.010, *P* = 0.006, and *P* = 0.037, respectively) (Fig. 4a), coincident with the improvement of case severity. A representative chest CT image of the meplazumab-treated patient was shown in Fig. 4b, which displayed the CT imaging manifestations (bilateral ground-glass shadow and consolidation) of COVID-19 at baseline period, and all these lesions were resolved at 22 days post treatment.

**Fig. 5:**
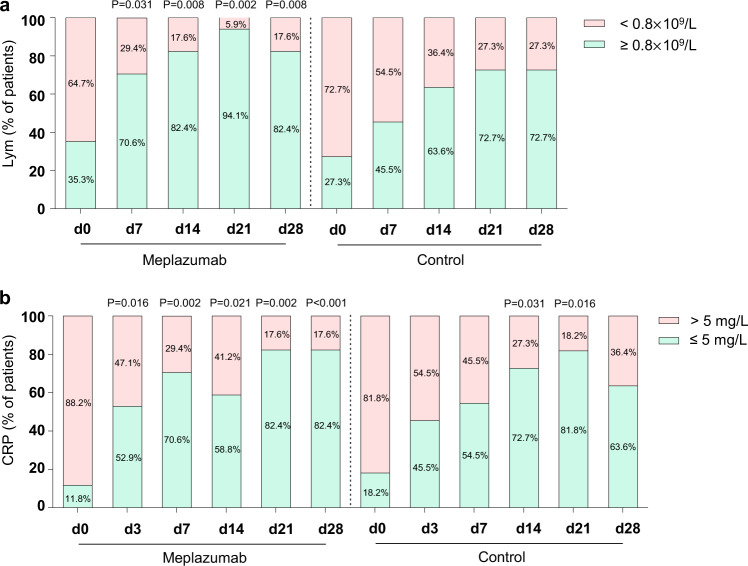
The proportion of patients regarding lymphocyte count and CRP concentration. The proportion of patients (% patients) with or without normal lymphocyte count (**a**) and CRP concentration (**b**). The *P* value comparing between baseline (d0) and each time point was determined by McNemar’s test, shown on the top of the column

It was reported that 75.4% of COVID-19 patients have lymphopenia.^15^ In this study, compared with baseline, the percentages of patients with a normal lymphocyte count (>0.8 × 10^9^/L) increased in both cohorts between Days 7 and 28, while the improvement in the meplazumab cohort was more notable (Fig. 5a). C-reactive protein (CRP) concentration serves as a predictor of COVID-19 severity.^16^ A significant increase in the percentages of patients with a normal CRP concentration (≤5 mg/L) was observed in the meplazumab cohort since Day 3 compared to baseline, while that was observed since Day 14 in the control cohort (Fig. 5b). The data suggest that meplazumab exhibited an effect on the control of virus-induced acute inflammation at early management.

## Discussion

With the rapid escalation of COVID-19, nearly 200 countries have reported >100 million cases and killed >2 million people globally, and the estimated case fatality rate is between 0.6 and 3.5%.^17,18^ Theoretically, vaccination is the best way to end the pandemic. Several successful COVID-19 vaccines have been approved for use in some counties, and nearly 2 million doses were administered every day globally.^19^ However, it has been reported that the duration of anti-SARS-CoV-2 is far less than SARS-CoV or Middle East respiratory syndrome coronavirus. The level of neutralizing antibodies in COVID-19 convalescent individuals start to drop within 2 to 3 months after infection, and the median half-life of the anti-SARS-CoV-2 IgG is 62 and 59 days.^20,21^ The impermanent immunity and ever-emerging SARS-CoV-2 variant will greatly impair the COVID-19 vaccination and will make SARS-CoV-2 enter into regular circulation, which was also projected by Kissler et al. using a deterministic model.^22^ All these facts indicate the necessity to develop specific therapeutic agents of COVID-19.

As of December 2020, >2300 randomized COVID-19 clinical trials have been registered, among which nearly one-tenth are antibody therapeutics.^23^ Most clinical trials used immunomodulatory monoclonal antibody drugs to modulate the immune response. Very few clinical trials used neutralizing antibodies, such as the REGN-COV2 cocktail and bamlanivimab, to block the direct entry and infection of SARS-CoV-2 to human cells. Different from neutralizing antibody, which interacts with the SARS-CoV-2 spike protein, meplazumab binds the receptor of spike protein to block the infection, and we believe this character could minimize the impact of SARS-CoV-2 variation on the therapeutic effect. Here, we reported a phase 1 study of meplazumab to assess safety, tolerability, PK, and biodistribution in healthy volunteers. To further investigate the efficacy, we conducted an exploratory phase 2 add-on trial of meplazumab in COVID-19 patients. For our information, this is the only paper with reports on targeting the receptor to spike protein and blocking the “gate” of SARS-CoV-2 to host cells.

In phase 1 clinical trial, the increase of blood bilirubin and blood-unconjugated bilirubin was reported as the highest incidence TEAEs in the single-dose study. The reasons for the increase of unconjugated bilirubin include hemolysis of erythrocytes, inhibition of bilirubin conjugating mechanisms in the liver, and abnormal hepatic uptake unconjugated bilirubin, transfusion reaction, scarring of the liver (cirrhosis), and the Gilbert syndrome.^24^ As an IgG_2_ antibody, meplazumab can bind the CD147 on erythrocytes’ surface and form antibody–antigen complexes. However, in our non-clinical study, 2 mg/mL of meplazumab, which is much higher than the highest concentration in this trial, did not cause human erythrocyte hemolysis in vitro.^13^ In the phase 1 trial, the increase of unconjugated bilirubin was also not correlated with the RO% of meplazumab on peripheral blood cells. Based on these data, we believed that meplazumab could not destroy erythrocytes via cytotoxicity either in an antibody-dependent cell-mediated manner or a complement-dependent manner. We presumed the blood-unconjugated bilirubin increase and blood bilirubin increase were caused by the destruction of the small population of sensitive erythrocytes (e.g., aging erythrocytes). Meplazumab bound CD147 on the surface of erythrocytes and formed antigen–antibody complex, which boosts the destruction of aging erythrocytes when passing through the capillary network. The increase of unconjugated bilirubin was transient and recovered within 48 h without medicinal intervention in our study. Due to the destruction of erythrocytes, erythrocytes’ released contents can activate the reticuloendothelial system, causing transient pyrexia and neutrophilic leukocytosis. After the removal of the sensitive erythrocyte population, the level of blood-unconjugated bilirubin and blood bilirubin was recovered, and all abnormalities disappeared within 48 h.

The results of PK of meplazumab indicate that meplazumab had a longer half-life time in the human body with the higher drug exposure and long-lasting receptor occupation, which may provide a high concentration antibody-drug reservoir to facilitate the penetration of the drug to target tissue. We confirmed that meplazumab binding to erythrocytes could dissociate from the cells and bind to lung epithelial cells, BEAS-2B, which suggests that erythrocytes serve as a reservoir for meplazumab (Supplementary Fig. 2). The results of the biodistribution study indicate that meplazumab can reach lung tissue and remain for no <14 days. Based on the results, in the initial 3 h from the infusion, the circulating meplazumab is absorbed by CD147 on target tissues. Three hours later, when the circulating meplazumab is used up, the whole blood cells serve as a reservoir, gradually releasing meplazumab.

The characteristics of PK, RO, and biodistribution of meplazumab also provide a reasonable explanation for the efficacy of meplazumab at a low dose in exploratory phase 2 study. Patients received 10 mg meplazumab three times within 5 days (~0.3 to 0.4 mg/kg total dose), and the dose was selected considering the nonhuman primate toxicology results to ensure the safety of clinical research.^13^ Based on the results of receptor occupation assay and PK, despite a relatively lower dose of meplazumab, it can still maintain a high receptor occupancy rate for a long time. The antibody reservoir formed by erythrocyte-bound meplazumab can continuously and stably release meplazumab, leading to a longer retention (>14 days) of meplazumab in the target tissue. In our unpublished data, a concentration-dependent RO rate was determined in Vero E6 to establish a correlation between meplazumab RO% and viral inhibition in Vero E6 cell. At the meplazumab concentration of 17.58 μg/mL, RO% on Vero E6 cells was modeled to be 47.19%, which resulted in ~50% viral inhibition in infected Vero E6 cells. At the concentration of 200 μg/mL, the RO% was calculated as 88.92%, which resulted in ~90% viral inhibition. Therefore, this lower dose is effective in maintaining therapeutic concentrations for a long period, which subsequently facilitates the efficacy of meplazumab in treating COVID-19.

A median time of ~15 days were reported for mild cases and 3–6 weeks for severe or critical patients conditions from the onset to clinical recovery.^25^ The patients’ condition usually deteriorates the second week from the onset of illness. For instance, the median time is 8–12 days for acute respiratory distress syndrome from the onset of illness .^1,26,27^ Some patients with COVID-19 who seem stable or on the mend can suddenly become critically ill during the second week, called “second-week crash.” In this study, the PK analysis indicated that *T*_1/2_ in a single dose was 25.5–40.67 days. Biodistribution analysis also demonstrated that meplazumab could reach lung tissue and be maintained for >14 days. These characteristics of meplazumab are instrumental to overcoming “second-week crash” and improving the prognosis of COVID-19 pneumonia, showing a reasonable prospect of meplazumab in the treatment of COVID-19 with a novel pharmacological mechanism.

It was reported that 18.5% of COVID-19 cases were categorized as severe and critical cases, with the fatality rate for critical cases reaching 49.0–61.5%.^1,3^ In the exploratory phase 2 trial, meplazumab treatment accelerated the improvement of COVID-19, especially in severe and critical cases. On Day 28, 69.2% of severe and critical cases were discharged, and no death case was reported. The improved chest imaging, clearance of virus, and recovered lymphocyte count were observed within 1-week management, which indicated that the therapeutic effects facilitated prognosis.

Lymphopenia was common in patients with COVID-19 and SARS patients, and can be used as an indicator for disease severity and prognosis.^28^ Due to the absence of ACE2 in lymphocytes, it has been proposed that the lymphocytopenia in SARS patients is caused by the indirect mechanism, including inflammation storm, vascular cell adhesion, soluble Fas ligand, and glucocorticoids.^10,29^ However, the SARS-CoV particles and genomic sequence were detected in a large number of circulating lymphocytes, destroying lymphocytes.^30^ In this study, lymphocyte count in meplazumab-treated COVID-19 patients was restored within 7 days. CD147 is highly expressed on the activated T cells,^31^ which facilitates the infection of SARS-CoV-2 pseudovirus to T lymphocytes by binding spike, suggesting that CD147 is involved in lymphocytopenia.^11^ We presume that meplazumab interrupts this process by preventing virus infection to keep lymphocytes survived.

The limitation of this study includes the insufficient cytokine data, as well as the small sample size in the exploratory phase 2 trial. Therefore, an international multi-center, seamless, randomized, third-party-blind, phase 2/3 trial is ongoing to fully assess the efficacy and safety of meplazumab in addition to standard of care for hospitalized adult patients with COVID-19.

Our results show the safety and tolerability of meplazumab in healthy subjects. Meplazumab is effective in treating hospitalized COVID-19 patients by specifically targeting host cell receptor CD147. All these results provide strong support for the ongoing international multi-center, randomized phase 2/3 clinical trial.

## Materials and methods

### Study design and participants

The phase 1 trial is a single-center, randomized, double-blinded, placebo-controlled clinical study that was registered at ClinicalTrials.gov (NO. NCT04369586) and conducted at Xijing Hospital in Xi’an, China. This trial was approved by the Independent Ethics Committee of the Xijing Hospital (YS202001001-X-1). Eight cohorts were planned, of which six cohorts were designed for a single dose (*n* = 8, 6 active/2 placebo), one cohort for a multiple dose (*n* = 8, 6 active/2 placebo), and one cohort for biodistribution (*n* = 3, 3 active). All enrolled subjects met the inclusion and exclusion criteria described in Supplementary Protocols 1 and 2. For the biodistribution study, three subjects received a single dose of ^131^I-labeled meplazumab. For the other seven cohorts, enrolled subjects were double-blind randomly assigned to meplazumab or placebo. No subject was involved in more than one cohort in the study.

The exploratory phase 2 trial is an open-labeled, concurrent controlled add-on clinical trial of meplazumab in hospitalized COVID-19 patients. The study was registered at ClinicalTrials.gov (No. NCT04275245). The study protocol was approved by the Independent Ethics Committee of the Institution for National Drug Clinical Trials at the Tangdu Hospital (K202002-01). In this trial, 39 patients diagnosed with COVID-19 were enrolled, with 17 patients assigned to the meplazumab cohort. Eleven hospitalized patients served as concurrent control. All patients received standard of care according to the *Chinese Clinical Guidance for COVID-19 Pneumonia Diagnosis and Treatments*.^14^ For the meplazumab cohort, 10 mg meplazumab was administered on Days 1, 2, and 5 by intravenous infusion.

### Randomization and masking

In the phase 1 trial, subjects received their subject identification number (screening number) as soon as they had signed the informed consent form. For each cohort, eligible subjects were randomly assigned in a double-blinded fashion to meplazumab or placebo in a ratio of 3:1, respectively. An unblinded statistician created the computer-generated randomization schedules before the initiation of the study. On Day −1 or Day 0, subjects were allocated a randomization number in sequential order, immediately before administration. A copy of the randomization schedule was sent to the clinical unit pharmacist and clinical unit project manager. Investigators remained blinded to each subject until all final clinical data entered the database, and all data queries were resolved, and the assignment of subjects to the analysis sets was completed. If judged necessary by the Safety Review Committee, an individual or the complete cohort may be unblinded during data evaluation.

As an open-labeled study, there is no need for blindness in the exploratory phase 2 trial.

### Outcomes

At the primary endpoint of the phase 1 trial, the occurrence rate of adverse events was assessed, including severe adverse events that were at least possibly related to the meplazumab infusions. A full list of endpoints was provided in the study protocol (Supplementary Protocol 1).

The primary efficiency endpoint of the exploratory phase 2 trial was the virological clearance (negative conservation rate and time to negative). Secondary efficacy endpoints were assessment of time to recovery of vital signs, chest radiographic improvement, rate of PaO_2_/FiO_2_ recovery, time (days) to discharge, and inflammation recovery (percentage of the patient with normal CRP concentration).

### Procedures

In the phase 1 trial, subjects were screened from Day −14 to Day −2. Information of their general condition, medical history, medication history, vital signs, physical examination, laboratory examination, electrocardiogram, and other related examinations was collected. In single-dose study, a single dose of meplazumab (0.06, 0.12, 0.2, 0.3, 0.42, and 0.56 mg/kg, respectively) or placebo was administered intravenously in 60 ± 5 min on the first day (Day 0). In the multiple-dose study, two doses were planned for a duration of 7 days in total. Subjects received the first dose (0.3 mg/kg meplazumab or placebo) on Day 0, and the second dose (0.3 mg/kg meplazumab or placebo) on Day 7 by an intravenous infusion in 60 ± 5 min. In the biodistribution study, a single-dose ^131^I-meplazumab of 0.3 mg/kg (total 10 mCi) was intravenously administered in 60 ± 5 min on Day 0. The investigators assessed safety, tolerability, PK, RO%, and immunogenicity (ADA analysis) at every scheduled visit, and the timing of each visit was described in the study protocol (Supplementary Protocols 1 and 2).

In the exploratory phase 2 trial, eligible patients received recommended treatment, subject to the *Chinese Clinical Guidance for COVID-19 Pneumonia Diagnosis and Treatment*.^14^ Physicians were allowed to use any necessary treatment, laboratory, and radiographic examination with their standard of care. Clinical, laboratory, and radiographic assessments were conducted at baseline, including complete blood count, serum biochemical test (renal function, liver function, and CRP), serum electrolytes test, coagulation analysis (prothrombin time, activated partial thromboplastin time, international normalized ratio, fibrinogen, d-dimer), chest computed tomography/chest X-ray, nasopharyngeal swab test for SARS-CoV-2 (using quantitative reverse transcription-PCR assay approved by the National Medical Products Administration). The investigators assessed the safety and efficacy assessed at every scheduled visit, and the timing of each visit was described in the study protocol (Supplementary Protocol 3).

### Pharmacokinetics and immunogenicity

The ADA in volunteers’ serum was examined using a validated Meso Scale Discovery (MSD) electrochemiluminescence method. RO% tests were performed by flow cytometry. PK parameters were determined by the concentration in serum and whole blood cells, and calculated using a non-compartment model (NCA) in WinNonlin 8.0.0. The protocol of PK parameter measurement and RO% test were described in the study protocol (Supplementary Protocol 1).

### Biodistribution

The meplazumab distribution was determined by the region of interest (ROI) radioactive counts in each organ, which were measured using a GE Xeleris 3 image workstation. The protocol of radioactive count measurement in each organ was described in the study protocol (Supplementary Protocol 2). The investigators assessed biodistribution at every scheduled visit, and the timing of each visit was described in the study protocol (Supplementary Protocol 2).

### ADA assay

As a heterologous protein, meplazumab may cause ADA in the human body and affect the safety and efficacy of treatment. The anti-drug binding antibody in volunteers’ serum was detected by a validated MSD method. In brief, the samples and quality control were diluted with 0.1 M acetic acid (v/v = 1:19) and incubated on a plate shaker (800 r.p.m.) at room temperature (19–25 °C) for 30 min. After the acidolysis reaction, the acidolysis sample was added Sulfo-labeled meplazumab (94 μL/well, 0.125 μg/mL, AbMax Biotechnology Co., Ltd), biotin-labeled meplazumab (0.0625 μg/mL, AbMax Biotechnology Co., Ltd) mixture, and 6 μL/well 1 M Tris-HCl (pH 9.5). Then, this mixture was sealed with dark seal plate film, and incubated on a plate shaker (800 r.p.m.) at room temperature (19–25 °C) for 2 h. The MSD SA plate was blocked by blocking buffer (150 µL/well), then covered with plate sealer, and incubated on a plate shaker (800 r.p.m.) at room temperature (19–25 °C) for 1 h. Following the incubations, the MSD plate was manually washed four times with a refill of 1× wash buffer (200 µL/well) for each wash. Samples and quality controls (50 μL/well) were transferred to the SA plate, and incubated at room temperature (19–25 °C) for 1 h. After wash, Read Buffer (150 µL/well) was applied and the plates at MESO QuickPlex SQ120 (MSD) were then monitored.

### Meplazumab releasing and rebinding assay

The plasma and erythrocytes were separated from 120 mL whole blood by centrifugation (3000 r.p.m. for 20 min at room temperature). Then, erythrocytes were resuspended to 120 mL with phosphate-buffered saline (PBS), and meplazumab was added to a final concentration of 1 mg/mL, followed by incubation of suspension at 37 °C for 1 h with gentle shaking. After that, erythrocytes were washed with PBS and resuspended with the plasma collected from the first step. Samples were aliquoted into 20 mL and cultured in a shaking incubator (37 °C, 180 r.p.m.) for 0, 24, 48, 72, 96, and 120 h, respectively. Then, the erythrocytes and supernatant were separated and collected by centrifugation (300 r.p.m. for 10 min at 4 °C).

To detect the meplazumab on erythrocytes, cells were incubated by using phycoerythrin (PE)-labeled anti-human IgG antibody (30 min at 4 °C), and the amount of meplazumab on erythrocytes was analyzed by flow cytometric.

For assessing the meplazumab concentration in the supernatant, the immunoradiometric assay was used. In brief, a rabbit anti-meplazumab polyclonal antibody was coated on the inner wall of the polystyrene test tube, and ^125^I-labeled CD147 was used to detect meplazumab. The radiation intensity was measured by radioimmunoassay γ counter. The counts per minute value of the sample was substituted into the standard curve to obtain the concentration of meplazumab.

For evaluating the meplazumab-rebinding ability, human normal lung epithelium cells, BEAS-2B cells, were used. Briefly, 1 × 10^6^ BEAS-2B cells were incubated with the 100 µL supernatant at 37 °C for 1 h, with the supernatant discarded, and PBS washed for three times, then resuspending the cells in 100 μl PBS. Resuspended BEAS-2B cells were reserved for incubation with PE-labeled anti-human IgG antibody at 4 °C for 30 min, and the meplazumab on BEAS-2B cells was tested by flow cytometric.

### Statistical analysis

The phase 1 trial is the first-in-human study of meplazumab, with no testing of formal statistical hypothesis conducted. The sample size in the phase 1 clinical trial was not based on formal statistical calculations, but was considered adequate to characterize the distribution of the planned endpoints. The primary PK parameters, e.g., *T*_max_, *C*_max_, and AUC_(0–*t*)_ were calculated by non-compartmental analysis. Student’s *t* test was performed for statistical analysis. The PK parameters of each subject were calculated using an NCA in the WinNonlin 8.0.0.3176 software.

In the exploratory phase 2 trial, the continuous variables were expressed as median (inter-quartile range) and compared with the Mann–Whitney *U* test. Categorical variables were defined as the number (%) and compared by Fisher’s exact test, Ordinal regression, or McNemar’s test. One hundred percent stacked column was drawn to describe the composition of case severity, chest radiographic improvement, lymphocyte count, and CRP concentration. Differences between discharged and virus nucleic acid-negative conversion curves were analyzed by Cox regression analysis. Multiple Cox regression was also used to determine potential confounding variables, including group, age, case severity, and glucocorticoid treatment. A two-sided *α* <0.05 was considered statistically significant. Statistical analyses were performed using the SPSS software, version 23.0, and GraphPad Prism software, version 5.0.

## Supplementary information

Supplementary Materials

Supplementary protocol 1

Supplementary protocol 2

Supplementary protocol 3

## Data Availability

The data that support the findings of this study are available from the corresponding authors upon reasonable request. Participant data without names and identifiers will be made available with approvals from all corresponding authors. After the publication of study findings, the data will be available for others to request. The research team will provide an email address for communication once the data are approved to be shared with others. A proposal with a detailed description of study objectives and statistical analysis plan will be needed for evaluation of the reasonability to request for our data. The corresponding authors will make a decision based on these materials. Additional materials may also be required during the process.
