# Supplementary material for: Safety and efficacy of meplazumab in healthy volunteers and COVID-19 patients: a randomized phase 1 and an exploratory phase 2 trial
Source: Signal Transduct Target Ther. 2021 May 17;6:194. doi: 10.1038/s41392-021-00603-6 (PMC8127508; doi:10.1038/s41392-021-00603-6)
Supplement: Supplementary file 1 — Supplementary Materials [file 41392_2021_603_MOESM1_ESM.pdf]

Supplementary Materials for

Safety and efficacy of meplazumab in healthy volunteers and COVID-19 patients: a randomized phase 1 and an exploratory phase 2 trial

Huijie Bian*#1, Zhao-Hui Zheng*2, Ding Wei*1, Aidong Wen*3, Zheng Zhang*1, Jian-Qi Lian*4, Wen-Zhen Kang*4, Chun-Qiu Hao*4, Jing Wang*5, Rong-Hua Xie[2], Ke Dong[6], Jie-Lai Xia[7], Jin-Lin Miao[1], Wen Kang[4], Guoquan Li[5], Di Zhang[3], Mingru Zhang[5], Xiu-Xuan Sun[1], Likun Ding[3], Kui Zhang[2], Junfeng Jia[2], Jin Ding[2], Zhiqin Li[2], Yanyan Jia[3], Lin-Na Liu[8], Zhe Zhang[6], Zhao-Wei Gao[6], Hong Du[4], Na Yao[4], Qing Wang[2], Ke Wang[1], Jie-Jie Geng[1], Bin Wang[1], Ting Guo[1], Ruo Chen[1], Yu-Meng Zhu[1], Li-Juan Wang[1], Qian He[1], Rui-Rui Yao[1], Ying Shi[1], Xiang-Min Yang[1], Jian-Sheng Zhou[1], Yi-Nan Ma[1], Ya-Tao Wang[1], Xue Liang[1], Fei Huo[1], Zhe Wang[9], Yang Zhang[1], Xu Yang[1], Ye Zhang[4], Lu-Hua Gao[4], Ling Wang[7], Xiao-Chun Chen[10], Hao Tang[10], Shuang-Shuang Liu[10], Qing-Yi Wang[11], Zhi-Nan Chen#1, Ping Zhu#2

Correspondence to: Ping Zhu (zhuping@fmmu.edu.cn); Zhi-Nan Chen (znchen@fmmu.edu.cn); Huijie Bian (hjbian@fmmu.edu.cn)

This PDF file includes:

Figures. S1 to S2
Tables S1 to S5

Other Supplementary Materials for this manuscript include the following:

Supplementary Protocol 1 to Supplementary Protocol 3

Figure. S1.

The SPECT images of ^{131}I-Meplazumab for subjects injected with 0.3 mg/kg (10 mCi).
The ROI of each tissue of subjects was shown in the left column of panel ANT, which was applied to ensure the same ROI of the same organ to avoid measurement bias at every time-point.

Figure S1

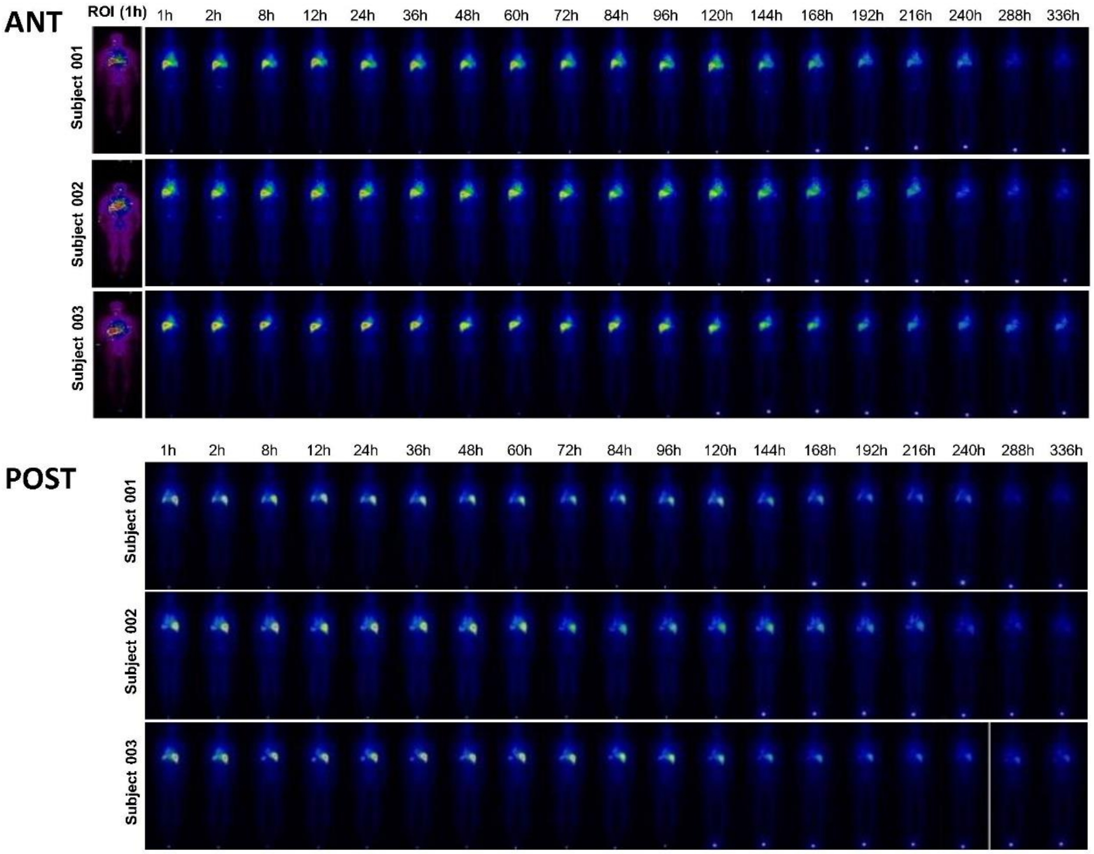

Figure. S2.

Meplazumab is released from human erythrocytes and rebinds to BEAS-2B cells.

(a) The mean fluorescent intensity of meplazumab on erythrocytes as detected by flow cytometry; (b) The concentration of meplazumab in the culture supernatant of erythrocytes; (c) The mean fluorescent intensity of released meplazumab binding to BEAS-2B cells as detected by flow cytometry. Y01-Y06 indicates erythrocytes obtained from six healthy volunteers.

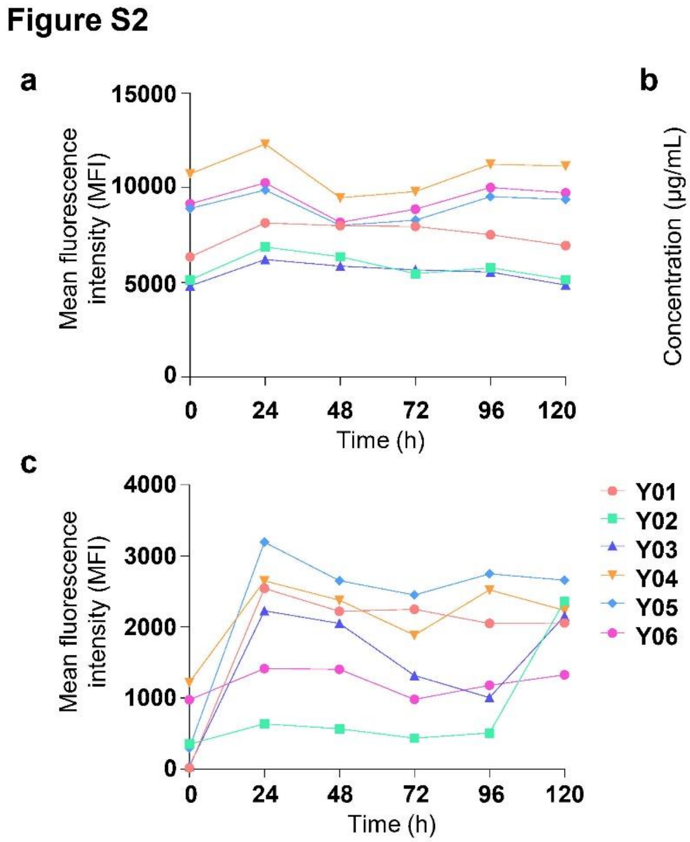

Table S1. Summary of TEAEs by system organ class and preferred term

System organ class preferred term	Single-dose							Multiple-dose	
	0.06 mg/kg (n=6) n(%), m	0.12 mg/kg (n=6) n(%), m	0.2 mg/kg (n=6) n(%), m	0.3 mg/kg (n=6) n(%), m	0.42 mg/kg (n=6) n(%), m	0.56 mg/kg (n=6) n(%), m	Placebo (n=12) n(%), m	0.3 mg/kg (n=6) n(%),m	Placebo (n=2) n(%),m
**TEAEs**	1(16.7), 4	6(100), 44	3(50.0), 12	6(100), 35	3(50.0), 16	6(100), 57	6(50.0), 12	5(83.3), 58	0
**Investigations**	1(16.7), 4	6(100), 43	3(50.0), 11	5(83.3), 33	3(50.0), 15	6(100), 46	4(33.3), 9		
Blood bilirubin increase	1(16.7), 1	5(83.3), 5	2(33.3), 2	4(66.7), 4	3(50.0), 3	4(66.7), 4	2(16.7), 2	4(66.7), 6	0
Blood bilirubin unconjugated increase	1(16.7), 1	5(83.3), 5	2(33.3), 2	4(66.7), 4	3(50.0), 3	4(66.7), 4	2(16.7), 2	4(66.7), 6	0
Bilirubin conjugated increase	1(16.7), 1	5(83.3), 5	1(16.7), 1	4(66.7), 4	2(33.3), 2	4(66.7), 4	1(8.3), 1	4(66.7),7	0
Neutrophil percentage increase	0	4(66.7), 4	0	2(33.3), 2	2(33.3), 2	5(83.3), 5	0	5(83.3), 6	0
Neutrophil count increase	0	4(66.7), 4	0	2(33.3), 2	2(33.3), 2	5(83.3), 5	0	5(83.3), 6	0
Lymphocyte percentage decrease	0	4(66.7), 4	0	2(33.3), 2	0	5(83.3), 5	0	5(83.3), 5	0
Lymphocyte count decrease	0	2(33.3), 2	0	2(33.3), 2	0	4(66.7), 4	0	4(66.7), 4	0
White blood cell count increase	0	3(50.0), 3	0	1(16.7), 1	1(16.7), 1	3(50.0), 3	0	3(50.0), 4	0
Blood lactate dehydrogenase increase	0	2(33.3), 2	1(16.7), 1	2(33.3), 2	1(16.7), 1	2(33.3), 2	0		
Monocyte percentage decrease	0	2(33.3), 2	0	2(33.3), 2	0	3(50.0), 3	0	2(33.3), 2	0
Eosinophil percentage decrease	0	1(16.7), 1	0	1(16.7), 1	0	2(33.3), 2	0	2(33.3), 2	0
Eosinophil count decrease	0	1(16.7), 1	0	1(16.7), 1	0	1(16.7), 1	0	2(33.3), 2	0
Eosinophil percentage increased								1(16.7), 1	0
Blood uric acid increase	0	1(16.7), 1	0	1(16.7), 1	0	0	1(8.3), 1		
Alanine aminotransferase increase	1(16.7), 1	0	1(16.7), 1	0	0	0	0		
Haemoglobin decrease	0	1(16.7), 1	0	0	0	0	1(8.3), 1	1(16.7), 1	0
Gamma-glutamyltransferase increase	0	0	0	0	0	1(16.7), 1	0		
Monocyte count increase	0	1(16.7), 1	0	0	0	0	0		
Monocyte count decrease	0	0	0	0	0	1(16.7), 1	0	2(33.3), 2	0
Eosinophil count increase	0	0	0	0	0	1(16.7), 1	0		
Aspartate aminotransferase increase	0	0	1(16.7), 1	0	0	0	0		
White blood cells urine positive	0	0	0	1(16.7), 2	0	0	0		
Electrocardiogram PR prolongation	0	1(16.7), 1	0	0	0	0	0		

**Abnormal electrocardiogram T wave**	**0**	**0**	**0**	**0**	**0**	**1(16.7), 1**	**0**		
**Electrocardiogram high voltage**	**0**	**1(16.7), 1**	**0**	**0**	**0**	**0**	**0**		
**Abnormal Haematocrit**	**0**	**0**	**0**	**0**	**0**	**0**	**1(8.3), 1**		
**Red blood cell count decrease**	**0**	**0**	**0**	**0**	**0**	**0**	**1(8.3), 1**	**1(16.7), 1**	**0**
**Bacterial test positive**	**0**	**0**	**0**	**1(16.7), 1**	**0**	**0**	**0**		
**Brain natriuretic peptide increase**	**0**	**0**	**0**	**0**	**1(16.7), 1**	**0**	**0**		
**Platelet count decrease**	**0**	**0**	**0**	**1(16.7), 1**	**0**	**0**	**0**		
**Myoglobin blood increase**	**0**	**0**	**1(16.7), 1**	**0**	**0**	**0**	**0**		
**Blood creatine phosphokinase MB increase**	**0**	**0**	**1(16.7), 1**	**0**	**0**	**0**	**0**		
**Blood creatine phosphokinase increase**	**0**	**0**	**1(16.7), 1**	**0**	**0**	**0**	**0**	**2(33.3), 2**	**0**
**Blood lactate dehydrogenase increased**									
**Blood glucose increase**	**0**	**0**	**0**	**1(16.7), 1**	**0**	**0**	**0**		
**General disorders and administration site conditions**	**0**	**1(16.7), 1**	**0**	**1(16.7), 1**	**1(16.7), 1**	**5(83.3), 5**	**0**	**1(16.7), 1**	**0**
**Pyrexia**	**0**	**1(16.7), 1**	**0**	**1(16.7), 1**	**1(16.7), 1**	**5(83.3), 5**	**0**	**1(16.7), 1**	**0**
**Cardiac disorders**	**0**	**0**	**0**	**0**	**0**	**1(16.7), 1**	**2(16.7), 2**		
**Bradycardia**	**0**	**0**	**0**	**0**	**0**	**1(16.7), 1**	**1(8.3), 1**		
**Ventricular extrasystoles**	**0**	**0**	**0**	**0**	**0**	**0**	**1(8.3), 1**		
**Renal and urinary disorders**	**0**	**0**	**0**	**1(16.7), 1**	**0**	**0**	**1(8.3), 1**		
**Haematuria**	**0**	**0**	**0**	**1(16.7), 1**	**0**	**0**	**1(8.3), 1**		
**Gastrointestinal disorders**	**0**	**0**	**0**	**0**	**0**	**2(33.3), 4**	**0**		
**Vomiting**	**0**	**0**	**0**	**0**	**0**	**2(33.3), 2**	**0**		
**Nausea**	**0**	**0**	**0**	**0**	**0**	**2(33.3), 2**	**0**		
**Musculoskeletal and connective tissue disorders**	**0**	**0**	**1(16.7), 1**	**0**	**0**	**0**	**0**		
**Pain in extremity**	**0**	**0**	**1(16.7), 1**	**0**	**0**	**0**	**0**		
**Nervous system disorders**	**0**	**0**	**0**	**0**	**0**	**1(16.7), 1**	**0**		
**Headache**	**0**	**0**	**0**	**0**	**0**	**1(16.7), 1**	**0**	**5(83.3), 57**	**0**

N: The number of safety analysis set subjects in each group;
n (%): The number and percentages of subjects with at least one TEAE;
m: Number of events.

Table S2 Summary of Anti-Drug Antibody (ADA) assay

Cohort		Positive ADA (antibody titer)			
		0	Day 28 (672h)	Day 56 (1344h)	Day 84 (2016h)
Single-dose	0.06 mg/kg	2/6 (1:4–1:16)	2/6 (1:4–1:16)	3/6 (1:2–1:32)	3/6 (1:4–1:32)
	0.12 mg/kg	0/6 (0)	3/6 (1:2–1:64)	4/6 (1:16–1:64)	4/6 (1:8–1:128)
	0.2 mg/kg	0/6 (0)	2/6 (1:1–1:128)	1/6 (1:128)	3/5 (1:32–1:512)
	0.3 mg/kg	0/6 (0)	1/6 (1:4)	1/6 (1:4)	0/6 (0)
	0.42 mg/kg	1/6 (1:4)	4/6 (1:4–1:16)	4/6 (1:4–1:32)	2/6 (1:32–1:128)
	0.56 mg/kg	0/6 (0)	2/6 (1:2–1:4)	1/6 (1:256)	2/6 (1:8–1:2048)
Multiple-dose	0.3 mg/kg	1/6 (1:2)	2/6 (1:2–1:64)	3/6 (1:4)	2/6 (<1–1: 8)

Table S3 Serum pharmacokinetic parameters

		N		$t_{1/2}$ (h)	T_{max} (h)	C_{max} (ng/mL)	AUC_{last} (h*ng/mL)	AUC_{INF_obs} (h*ng/mL)	V_{z_obs} (mL/kg)	C_{l_obs} (mL/h/kg)	MRT_{last} (h)	AF
Single-dose	0.06 mg/kg	6	Mean	85.92	1.25	0.46	0.99	36.93	201392.5	1624.66	..	..
			SD	NA	1.37	0.17	1.82	NA	NA	NA	..	..
	0.12 mg/kg	6	Mean	NA	0.88	1.2	2.54	NA	NA	NA	..	..
			SD	NA	0.31	0.65	3.78	NA	NA	NA	..	..
	0.2 mg/kg	6	Mean	NA	1	3.44	2.57	NA	NA	NA	..	..
			SD	NA	0	2.16	1.78	NA	NA	NA	..	..
	0.3 mg/kg	6	Mean	39.19	1	20.62	12.05	36.64	462930.9	8187.37	..	..
			SD	NA	0	11.05	6.48	NA	NA	NA	..	..
	0.42 mg/kg	6	Mean	1.14	1	51.56	30.89	29.97	27316.11	17257.15	..	..
			SD	0.10	0	20.7	11.31	12.18	14393.16	10750.24	..	..
	0.56 mg/kg	6	Mean	3.25	0.83	26.42	22.55	25.47	101896.5	23483.96	..	..
			SD	3.03	0.26	4.23	7.01	7.48	75180.98	6198.35	..	..
Multiple-dose	D1	6	Mean	92.73	1	32.98	20.38	57.3	506039.6	6888.67	4.39	
			SD	124.68	0	21.69	13.41	38.57	429798.8	3468.62	4.74	
	D7	6	Mean	19.61	0.58	43.72	48.58	56.65	170750.6	7413.35	5.9	1.38
			SD	27.76	0.2	17.96	22.79	28.03	211299.2	5848.66	9.18	

AF: accumulation factor

Table S4 Pharmacokinetic parameters of meplazumab binding to blood cells

		N		$t_{1/2}$ (h)	T_{max} (h)	C_{max} (μg/mL)	AUC_{last} (h·mg/mL)	AUC_{inf} (h·mg/mL)	Vd (mL/kg)	Cl (mL/h/kg)	MRT (h)	$AUC_{0\text{-}168}$ (h·mg/mL)	AF
Single-dose	0.06 mg/kg	n=6	Mean	976	48.5	0.219	214	281	303	0.215	781	··	··
			SD	82.4	62.1	0.0212	22.7	29.8	45	0.0234	10.3	··	··
	0.12 mg/kg	n=6	Mean	685	92.5	0.386	331	409	270	0.313	671	··	··
			SD	397	71.1	0.0195	49.8	116	87	0.084	132	··	··
	0.2 mg/kg	n=6	Mean	755	76.0	0.838	523	664	323	0.316	610	··	··
			SD	229	41.3	0.208	100	155	48.1	0.0764	162	··	··
	0.3 mg/kg	n=6	Mean	736	3.58	3.28	715	844	385	0.382	618	··	··
			SD	179	3.44	1.59	184	229	71.2	0.123	145	··	··
	0.42 mg/kg	n=6	Mean	622	20.8	4.72	823	914	421	0.483	548	··	··
			SD	134	22.8	3.20	177	228	71.8	0.112	74.6	··	··
	0.56 mg/kg	n=6	Mean	615	36.9	4.35	861	954	515	0.621	486	··	··
			SD	198	55.1	2.30	181	250	102	0.157	131	··	··
Multiple-dose	D1	n=6	Mean	128	3.42	2.32	166	299	197	1.09	78.0	166	··
			SD	59.1	4.21	1.19	46.5	105	117	0.295	4.41	46.5	··
	D7		Mean	566	10.1	4.92	798	871	284	0.406	449	287	1.76
			SD	199	18.8	2.55	289	351	28.7	0.205	101	93.1	0.381

AF: accumulation factor

Table S5 Demographics and baseline characteristics of patients with COVID-19

	Meplazumab (n=17)	Control (n=11)	p value
**Characteristics**			
Age, years	51 (49–67)	64 (43–67)	0.981
Sex			0.441
Women	6 (35.3%)	6 (54.5%)	
Men	11 (64.7%)	5 (45.5%)	
Current smoking	1 (5.9%)	1 (9.1%)	1.000
Epidemiological exposure			0.934
No exposure	6 (35.3%)	5 (45.5%)	
Hubei exposure	10 (58.8%)	4 (36.4%)	
Confirmed patient exposure	1 (5.9%)	2 (18.2%)	
Any comorbidity	9 (52.9%)	4 (36.4%)	0.460
Diabetes	3 (17.6%)	0 (0%)	0.258
Hypertension	6 (35.3%)	3 (27.3%)	1.000
Cardiovascular disease	1 (5.9%)	2 (18.2%)	0.543
Chronic obstructive pulmonary disease	1 (5.9%)	0 (0%)	1.000
Parkinson disease	1 (5.9%)	0 (0%)	1.000
**Treatment**			
Antiviral treatment			
Lopinavir and Ritonavir	17 (100%)	11 (100%)	NA
Recombinant human interferon α-2b	17 (100%)	11 (100%)	NA
Glucocorticoid	16 (94.1%)	7 (63.6%)	0.062
Antibiotic	17 (100%)	10 (90.9%)	0.393
**Signs and symptoms**			
Fever	16 (94.1%)	11 (100%)	1.000
Highest temperature, ℃			0.914
<37.3	1 (5.9%)	0 (0%)	
37.3–38.0	9 (52.9%)	7 (63.6%)	
38.1–39.0	5 (29.4%)	2 (18.2%)	
>39.0	2 (11.8%)	2 (18.2%)	
Cough	9 (52.9%)	7 (63.6%)	0.705
Myalgia or fatigue	10 (58.8%)	5 (45.5%)	0.700
Sputum production	1 (5.9%)	2 (18.2%)	0.543
Diarrhoea	2 (11.8%)	1 (9.1%)	1.000
Dyspnoea	10 (58.8%)	7 (63.6%)	1.000
Respiratory rate >24 breaths per min	7 (41.2%)	5 (45.5%)	1.000

**Case severity**			0.388
Common	4 (23.5%)	4 (36.4%)	
Severe	6 (35.3%)	4 (36.4%)	
Critical	7 (41.2%)	3 (27.3%)	
**Mechanical ventilation**	5 (29.4%)	3 (27.3)	1.000

Case severity is categorized according to the Diagnosis and Treatment for 2019 Novel Coronavirus Diseases released by the National Health Commission of China.
Data are median (IQR) or n (%). p values comparing Meplazumab and control are from Fisher's exact test, Mann-Whitney U test, or Ordinal regression. NA = not applicable.

Supplementary Protocol 1 (separate file)

Protocol for a single-center, randomized, double-blinded, placebo-controlled phase 1 trial of Meplazumab for Injection in healthy volunteer.

Supplementary Protocol 2 (separate file)

Protocol for a biodistribution study of ^{131}I-labelled meplazumab in healthy volunteer in the phase 1 trial of Meplazumab for Injection.

Supplementary Protocol 3 (separate file)

Protocol for the exploratory phase 2 trial of Meplazumab for Injection in patient with COVID-19 pneumonia.
