# Supplementary material for: Safety and efficacy of meplazumab in healthy volunteers and COVID-19 patients: a randomized phase 1 and an exploratory phase 2 trial
Source: Signal Transduct Target Ther. 2021 May 17;6:194. doi: 10.1038/s41392-021-00603-6 (PMC8127508; doi:10.1038/s41392-021-00603-6)
Supplement: Supplementary file 2 — Supplementary protocol 1 [file 41392_2021_603_MOESM2_ESM.pdf]

TITLE PAGE

Protocol Title:

A Single-Center, Double-blinded, Placebo-controlled Phase I Clinical Trial in Healthy Volunteer to Evaluate Safety, Tolerance, and Pharmacokinetics of Meplazumab for Injection

Protocol Number: MPZ-I-01

Amendment Number: V3.0

Product: Meplazumab for Injection (Ketantin®)

Short Title:

A Phase I Clinical Trial of Meplazumab in Healthy Volunteer

Study Phase: Phase I

Sponsor Name:

Jiangsu Pacific Meinuoke Biopharmaceutical Co. LTD.

National Translational Science Centre for Molecular Medicine of Fourth Military Medical University

Legal Registered Address:

No. 128 Hehai West Road, Xinbei District, Changzhou, 213022, Jiangsu People's Republic of China

Principal Investigator: Prof. Ping Zhu, MD, Prof. Aidong Wen, MD

Regulatory Agency Identifying Number:

Date of Protocol: 05 June 2020

Sponsor Signatory:

I have read this protocol in its entirety and agree to conduct the study accordingly:

**Xiaochun Chen**	**Date**
**Zhi-Nan Chen**	**Date**

.

Title Page 1
1. Protocol Summary 5
Synopsis 5
Schedule of Activities 9
Assessment Time Windows 16
2. Introduction 17
2.1 Study Rationale 17
2.2 Background 17
2.3 Benefit/Risk Assessment 23
2.4 Meplazumab 23
3 Objectives and Endpoints 25
4 Study Design 26
4.1. Overall Design 26
4.2. Scientific Rationale for Study Design 29
4.3. Study Population 29
4.4. Safety Assessments 29
4.5. Study Design 29
4.6. Justification for Dose of Meplazumab 31
4.7. End of Study Definition 32
4.8. Study Stopping Criteria 32
5 Study Population 33
5.1. Inclusion Criteria 33
5.2. Exclusion Criteria 33
5.3. Enrollment 34
6 Study Treatment 35
6.1. Study Treatment(s) Administered 35
6.2. Preparation/Handling/Storage/Accountability 35
6.3. Measures to Minimize Bias: Randomization and Blinding 36
6.4. Study Treatment Compliance 38
6.5. Prior and Concomitant Therapy 38
6.6. Dose Modification 39
6.7. Treatment After the End of the Study 39
7 Study Assessments and Procedures 40
7.1 Schedule of Assessments 40
7.2 Screening Examinations 40
7.3 Treatment Period 41
7.4 Outpatient Visits and Phone Calls 41
7.5 End of Study/Early Termination Visit 41
7.6 Demographics and Other Baseline Characteristics 41
7.7 Medical and Surgical History 42
7.8 Safety Assessments 42
7.9 Adverse Events 45
7.10 Pregnancy 47
7.11 Pharmacokinetics 47

7.12 Receptor Occupancy Assay **48**
8 Statistical Considerations **49**
8.1 Statistical Hypotheses **49**
8.2 Sample Size Determination **49**
8.3 Populations **49**
8.4 Statistical Analyses **50**
8.5 Study Population Data **50**
8.6 Efficacy Analyses **51**
8.7 Pharmacokinetic and Pharmacodynamic Analyses **51**
8.8 Safety Analyses **52**
8.9 Missing Data **53**
8.10 Interim Analyses **53**
9 References **54**
10 Appendices **55**
Appendix 1 Abbreviations **55**
Appendix 2 Regulatory, Ethical and Study Oversight Considerations **57**
Regulatory and Ethical Considerations 57
Adequate Resources 57
Financial Disclosure 58
Insurance 58
Informed Consent Process 58
Data Protection 59
Dissemination of Clinical Study Data 60
Data Quality Assurance 60
Source Documents 61
Protocol Deviations 61
Study and Study Center Closure 62
Publication Policy 63
Appendix 3 .... Adverse Events: Definitions and Procedures for Recording, Evaluating, Follow-up, and Reporting **65**
Signature of Investigator **71**

1. PROTOCOL SUMMARY

Synopsis

Protocol Title:

A Single-Center, Double-blinded, Placebo-controlled Phase I Clinical Trial in Healthy Volunteer to Evaluate Safety, Tolerance, and Pharmacokinetics of Meplazumab for Injection

Short Title:

A Phase I Clinical Trial of Meplazumab in Healthy Volunteer

Rationale:

This Phase I study will be conducted to explore the dose regimen in humans and to evaluate the safety, tolerability, pharmacokinetics (PK), receptor occupancy (RO) rate on blood cells of meplazumab in healthy subjects, thus providing a new macromolecule antibody drug for the treatment of COVID-19 pneumonia.

Objectives and Endpoints:

The primary and secondary endpoints of the study are presented below.

**Objectives**	**Endpoints**
Primary	
• To evaluate the safety and tolerability of single ascending doses and multiple doses of meplazumab in healthy subjects.	For subjects who have received a single dose and multiple doses of meplazumab or placebo:<br>• Nature, incidence, and severity of AEs/SAEs, including relationship to study treatment, AEs/SAEs leading to discontinuation of the study
Secondary	
• To evaluate meplazumab PK in blood following single-dose and multiple-dose administration.	• Meplazumab blood concentration-time profiles and calculated PK parameters, including but not limited to $AUC_{(0\text{-}tn)}$, $AUC_{(0\text{-}\infty)}$, and $T_{½}$, etc.
• To evaluate receptor occupancy of meplazumab in peripheral blood cells	• Rate of receptor occupancy in peripheral blood cells after the administration of meplazumab
• To evaluate laboratory examination of meplazumab	• Laboratory examination before and after the administration of meplazumab

AE = adverse event; $AUC_{(0\text{-}inf)}$ = area under the concentration-time curve extrapolated to infinity; $AUC_{(0\text{-}last)}$ = area under the concentration-time curve to the last quantifiable concentration; PK = pharmacokinetic; RO = receptor occupancy; SAE = serious adverse event; $T_{½}$ = elimination half-life.

Overall Design:

It is a single-center, randomized, double-blind, placebo-controlled, dose-escalation study. Seven cohorts are planned, and each cohort will consist of 8 or more subjects with 6 or more subjects receiving meplazumab and 2 subjects receiving placebo, for a total of 56 or more subjects.

The dose limited toxicity (DLT) will be observed, and the pharmacokinetic (PK) will be evaluated during the study period.

For single-dose administration, six cohorts (0.06mg / kg, 0.12mg / kg, 0.2mg / kg, 0.3mg / kg, 0.42mg / kg, 0.56mg / kg, respectively) are planned. At least 8 subjects will be enrolled in each cohort, 6 will be administrated meplazumab, and 2 with placebo. If no adverse events were observed in the highest dose group, and the PK results suggest that it has not saturation, the sponsor and the investigator could add an extra dose group to investigate the single-dose PK. Subjects will be screened from Day -14 to Day -2, the information of general condition, medical and medication history, vital signs, examination, laboratory examination (blood routine, urine routine, hepatic and renal function, coagulation function), ECG, drug abuse, and other related examinations of subjects will be collected. Subjects who meet the inclusion criteria will be admitted to the study center on Day -1 and be confined to the study center under medical supervision. On the first day (Day 0), a single dose of meplazumab or placebo will be administered as an intravenous infusion over 60 minutes. Subjects will be confined to the clinic until the morning of Day 7, and blood samples were collected according to the schedule of assessments at multiple time points until 168h after the administration. Subjects will return to the study center for follow-up visits on Days 10 (240h), 14 (336h), 21 (504h), 28 (672h), 38(912h), 48(1152h), 56 (1344h), and 84 (2016h) for safety, tolerability, PK, RO% (receptor occupancy), and immunogenicity assessments. On day 8-9, Day 11-13, Day 15-20, Day 22-27, Day 29-37, Day39-47, Day49-55, Day 57-83, the safety evaluation will be completed by telephone follow-up.

For multiple-dose study: the dosage and frequency of administrations should be adjusted according to the results of the pilot study and single-dose study. The initially set dosage is 0.3mg/kg. Subjects will be screened from Day -14 to Day -2, the information of general condition, medical and medication history, vital signs, physical examination, laboratory examination (blood routine, urine routine, hepatic and renal function, coagulation function), ECG, drug abuse, and other related examinations of subjects will be collected. Subjects who meet the inclusion criteria will be admitted to the study center on Day -1 and be confined to the study center under medical supervision. On the first day (Day 0), the first dose of meplazumab or placebo will be administered as an intravenous infusion over 60 minutes. The second dose of meplazumab or placebo will be administered on Day 7 as an intravenous infusion over 60 minutes. Subjects will be confined to the clinic until the morning of Day 7 after the second administration. Blood samples were collected according to the assessments' schedule at multiple time points until 168h after the second administration. Subjects will return to the study center for follow-up visits on Days 10 (240h), 14 (336h), 21 (504h), 28 (672h), 38(912h), 48(1152h), 56 (1344h), and 84 (2016h) after second administration for safety, tolerability, PK, RO% (receptor occupancy), and immunogenicity assessments. On day 8-9, Day 11-13, Day 15-20, Day 22-27, Day 29-37, Day39-47, Day49-55, Day 57-83 after the second administration, the safety evaluation will be completed by telephone follow-up.

The safety evaluation should be performed during the screening period, baseline period, observation period, and before leaving the group. The investigator can increase the safety check according to the actual situation. All abnormal and clinically significant changes of the subjects need to be followed up until they return to normal, or the investigator believes that there is no clinical significance.

Number of Investigators and Study Centers:

The study will be conducted at a single study center under the supervision of a Principal Investigator.

Number of Subjects:

Approximately 56 or more healthy men and women (18 to 50 years of age) will participate in this study.

Treatment Groups and Duration:

Six cohorts are planned for single-dose study. In each cohort, subjects will receive a single dose of either meplazumab or placebo.

One cohort is planned for multiple-dose study. In this cohort, subjects will receive multiple doses of either meplazumab or placebo.

Criteria for Stopping Dose Escalation and Study:

Dose escalation will be stopped if any of the stopping rules are met:

- If one-third of the subjects have Grade 3 AEs in the same organ system, which are considered at least possibly related to study treatment, the subjects will be unblinded. If both meplazumab-treated and placebo-treated subjects are included, the therapeutic monitoring will be strengthened, the investigator and sponsor will decide whether to conduct the dose escalation. If only meplazumab-treated subjects are included, the study will be terminated immediately.
- If half subjects have a Grade 2 AE in the same organ or body system which is considered at least possibly related to study treatment, the subjects will be unblinded. If both group subjects are included, the therapeutic monitoring will be strengthened, and the dose escalation will continue. If only treatment group subjects are included, the investigator and sponsor will decide whether to conduct the dose escalation or terminate the study.
- If the proportion of subjects who have CTCAE Grade 2 (or higher) AEs in the same organ system are less than 50% for Grade 2 or less than one-third for Grade 3, the investigator and sponsor will decide whether to conduct the dose escalation.
- When the severity or proportion of AEs is higher, the risk control measures should be performed.

Statistical Methods:

Determination of Sample Size

According to the "Technical Guidelines for Clinical Pharmacokinetic Research of Chemical Drugs," 8 to 12 subjects in each dose group are required. In this study, 8 subjects in each dose group are planned. There are 6 dose groups in the single-dose study and 1 dose group in the multiple-dose study. In each dose group, 6 subjects receive study medication, and 2 subjects received placebo. A total of at least 56 patients are required.

Analysis Sets

Full analysis set: define as all subjects after randomization.

Safety analysis set: define as all enrolled subjects who have received a single dose of meplazumab or placebo and have undergone at least one safety assessment after baseline.

PK analysis set: defined as all subjects who have received the study drug and have at least one calculable pharmacokinetic parameter result, and no major protocol violations that affect pharmacokinetics have occurred.

The RO% Analysis Set: All subjects who have received a single dose of meplazumab or placebo and have at least 1 RO% result collected post-dose without significant protocol deviations/violations or events thought to affect the RO% significantly.

Safety Analyses

All safety analyses will be performed on the Safety Analysis Set. Adverse events will be coded using the Medical Dictionary for Regulatory Activities. The overall number and percentage of subjects with at least one AE (and SAE) will be tabulated over the entire study period. Overall, AE data will be summarized by treatment received and study period. For each study treatment and study period, the frequency of all treatment-emergent AEs (TEAEs), TEAEs by maximum severity, TEAEs by relationship to study treatment, SAEs, TEAEs leading to death, and TEAEs leading to discontinuation of the study will be tabulated by preferred term and system organ class for each treatment group.

Pharmacokinetic Analyses

The PK analyses will be performed on the PK Analysis Set. meplazumab blood concentrations and calculated PK parameters will be listed and descriptively summarized separately. Results will be presented by treatment dose level, study day, and scheduled time point, as applicable. Arithmetic mean (±standard deviation) and individual concentration-versus-time curves will be plotted on a linear and semilogarithmic scale, as appropriate.

Schedule of Activities

Table 1 Schedule of Assessments (single-dose administration) :

Assessments	Screening				Treatment Period(day)																						EOS /ET
Day	**14--2D**	-1D	0										1	2	3	4	5	6	7	10	14	21	28	38	48	56	84
**Time relative to dose (h)**			Pre	0 (EOI)	15min	30min	1h	1.5h	2h	4h	8h	12h	24h	48h	72h	96h	120h	144h	168h	240h	336h	**504h**	**672h**	**912h**	**1157h**	**1344h**	**2016h**
Visits [b]	X	X	X	X	X	X	X	X	X	X	X	X	X	X	X	X	X	X	X Dis	X	X	X	X	X	X	X	X
Informed Consent	X																										
Demography	X																										
Eligibility Criteria check / recheck	X	X																									
Medical and Medication history	X																										
Smoking and drinking history, allergy history, blood donation history	X																										
Height and BMI	X																										
Physical examination	X																		X	X	X	X	X			X	X
Vital sign	X	X	X				X		X	X	X		X	X	X	X	X	X	X	X	X	X	X			X	X
Blood alcohol test		X																									
Urine drug abuse screening	X																										
12 lead ECG	X									X			X	X					X				X			X	X
Blood routine test	X		X							X			X	X					X				X			X	X
Urine routine test	X									X			X	X					X				X			X	X
Blood biochemistry	X		X							X			X	X					X				X			X	X
Myocardial enzyme			X							X			X	X					X				X			X	X
Coagulation function	X		X							X			X	X					X				X			X	X
Serology (HIV, HBV, HCV), RPR (syphilis)	X																										
SARS-CoV-2 Specific antibody detection	X																										
Pregnancy Test c/FSH Test (women only)	X																										X
Adm				X																							

Infusion-site reaction Assessment			X										X	X														
PK Sample Collection [i]			X		X	X	X	X	X	X	X	X	X	X	X	X	X	X	X	X	X	X	X	X	X	X	X	X
Receptor occupancy			X		X	X	X	X	X	X	X	X	X	X	X	X	X	X	X	X	X	X	X	X	X	X	X	X
Immunogenicity Assessment			X																					X			X	X
Recording of combination therapy		X	X	X	X	X	X	X	X	X	X	X	X	X	X	X	X	X	X	X	X	X	X	X	X	X	X	X
Recording of AEs/SAEs		X	X	X	X	X	X	X	X	X	X	X	X	X	X	X	X	X	X	X	X	X	X	X	X	X	X	X

Table 2 Schedule of Assessments (multiple-dose administration) :

Assessments	Screening		Treatment Period (1st dose)															
Day	**14--2**	-1	0										1	2	3	4	5	6
**Time relative to dose (h)**			Pre	0 (EOI)	15min	30min	1h	1.5h	2h	4h	8h	12h	24h	48h	72h	96h	120h	144h
Visits [b]	X	X	X	X	X	X	X	X	X	X	X	X	X	X	X	X	X	X
Informed Consent	X																	
Demography	X																	
Eligibility Criteria check / recheck	X	X																
Medical and Medication history	X																	
Smoking and drinking history, allergy history, blood donation history	X																	
Height and BMI	X																	
Physical examination	X																	
Vital sign	X	X	X				X		X	X	X	X	X	X	X	X	X	X
Blood alcohol test		X																
Urine drug abuse screening	X																	
12 lead ECG	X									X			X	X				
Blood routine test	X		X							X			X	X				
Urine routine test	X									X			X	X				
Blood biochemistry	X		X							X			X	X				
Myocardial enzyme			X							X			X	X				
Coagulation function	X		X							X			X	X				
Serology (HIV, HBV, HCV), RPR (syphilis)	X																	
SARS-CoV-2 Specific antibody detection	X																	
Pregnancy Test c/FSH Test (women only)	X																	
Adm				X														
Infusion-site reaction Assessment [e]			X										X	X				
PK Sample Collection [i]			X		X	X	X	X	X	X	X	X	X	X	X	X	X	X
Receptor occupancy			X		X	X	X	X	X	X	X	X	X	X	X	X	X	X
Immunogenicity Assessment			X															
Recording of combination therapy		X	X	X	X	X	X	X	X	X	X	X	X	X	X	X	X	X
Recording of AEs/SAEs		X	X	X	X	X	X	X	X	X	X	X	X	X	X	X	X	X

Assessments	Treatment Period (2nd dose)																
Day	0 (D7 post 1st Adm)										1	2	3	4	5	6	7
**Time relative to dose (h)**	Pre	0 (EOI)	15min	30min	1h	1.5h	2h	4h	8h	12h	24h	48h	72h	96h	120h	144h	168h
Visits [b]	X	X	X	X	X	X	X	X	X	X	X	X	X	X	X	X	X<br>Dis
Informed Consent																	
Demography																	
Eligibility Criteria check / recheck																	
Medical and Medication history																	
Smoking and drinking history, allergy history, blood donation history																	
Height and BMI																	
Physical examination																	X
Vital sign	X				X		X	X	X	X	X	X	X	X	X	X	X
Blood alcohol test																	
Urine drug abuse screening																	
12 lead ECG								X			X	X					X
Blood routine test	X							X			X	X					X
Urine routine test								X			X	X					X
Blood biochemistry	X							X			X	X					X
Myocardial enzyme	X							X			X	X					X
Coagulation function	X							X			X	X					X
Serology (HIV, HBV, HCV), RPR (syphilis)																	
SARS-CoV-2 Specific antibody detection																	
Pregnancy Test c/FSH Test (women only)																	
Adm		X															
Infusion-site reaction Assessment [e]	X										X	X					
PK Sample Collection [i]	X		X	X	X	X	X	X	X	X	X	X	X	X	X	X	X
Receptor occupancy	X		X	X	X	X	X	X	X	X	X	X	X	X	X	X	X
Immunogenicity Assessment																	
Recording of combination therapy	X	X	X	X	X	X	X	X	X	X	X	X	X	X	X	X	X
Recording of AEs/SAEs	X	X	X	X	X	X	X	X	X	X	X	X	X	X	X	X	X

Assessments	Treatment Period (2nd dose)							
Day	10	14	21	28	38	48	56	84
**Time relative to dose (h)**	240h	336h	504h	672h	912h	1152h	1344h	2016h
Visits [b]	X	X	X	X	X	X	X	X
Informed Consent								
Demography								
Eligibility Criteria check / recheck								
Medical and Medication history								
Smoking and drinking history, allergy history, blood donation history								
Height and BMI								
Physical examination	X	X	X	X			X	X
Vital sign	X	X	X	X			X	X
Blood alcohol test								
Urine drug abuse screening								
12 lead ECG				X			X	X
Blood routine test				X			X	X
Urine routine test				X			X	X
Blood biochemistry				X			X	X
Myocardial enzyme				X			X	X
Coagulation function				X			X	X
Serology (HIV, HBV, HCV), RPR (syphilis)								
SARS-CoV-2 Specific antibody detection								
Pregnancy Test c/FSH Test (women only)								
Adm								
Infusion-site reaction Assessment [e]								
PK Sample Collection [i]	X	X	X	X	X	X	X	X
Receptor occupancy	X	X	X	X	X	X	X	X
Immunogenicity Assessment				X			X	X
Recording of combination therapy	X	X	X	X	X	X	X	X
Recording of AEs/SAEs	X	X	X	X	X	X	X	X

Abbreviations: Adm = admission; AE = adverse event; BMI = body mass index; Dis = discharge; ECG = electrocardiogram; EOI = end of infusion; EOS = End of Study; ET = early termination; HIV = human immunodeficiency virus; PK = pharmacokinetic; RO = receptor occupancy; RPR = rapid plasma regain; SAE = serious adverse event.

General: All postdose time points are scheduled relative to the end of infusion (EOI). At visits where assessment time points coincide with each other, the following procedure should be followed: perform ECGs first, followed by vital signs as close as possible to the scheduled time point but prior to PK and RO sampling; perform PK and RO blood sampling at the scheduled time point; perform all other procedures as close as possible to the scheduled time point but may be obtained before or after PK and RO sampling.

Table 3 Content of Assessment

Assessments	Contents
Physical examination	General appearance, examination of skin, neck (including thyroid), eyes, ears, nose, throat, lungs, heart, abdomen, superficial lymph nodes, limbs, and nervous system
Vital sign	Pulse, blood pressure, body temperature, breathing rate;
Urine drug abuse screening	Morphine, ketamine, methamphetamine, marijuana, ecstasy, K powder, amphetamine, etc.
Serology (HIV, HBV, HCV), RPR (syphilis)	HBsAg, HIV-Ab, HCV-Ab, RPR (syphilis)
ECG	12-lead ECG
Blood routine test	Red blood cell count, white blood cell count, neutrophil percentage, lymphocyte percentage, mononuclear cell percentage, platelet count, hemoglobin, etc.
Urine routine test	Urine PH, urine protein, urine glucose, urine ketone body, urine red blood cells, urine white blood cells, etc.
Blood biochemistry	Alanine aminotransferase (ALT), aspartate aminotransferase (AST), total bilirubin (TBIL), straight bilirubin (DBIL), total protein (TP), albumin (ALB), globulin, alkaline phosphate enzymes (ALP), lactate dehydrogenase, creatine kinase (CK), glucose (GLU), urea (BUN), blood creatinine (Cr), etc.
Myocardial enzyme	N-terminal-B-type natriuretic peptide precursor, troponin I, myoglobin, creatine kinase-MB subtype quality
Coagulation function	Prothrombin time (PT), thrombin time (TT), fibrinogen (FIB), activated part thrombin time (APTT)
SARS-CoV-2 Specific antibody detection	Anti-SARS-CoV-2 IgM, anti-SARS-CoV-2 IgG.

Assessment Time Windows

Table 4 Allowed Time Windows for PK (All Parts)/ RO% (Part A) blood samples

No	Time point	Tolerance window	Interval of time point	Time window calculation method (theoretical time deviation)
1	2min 120s)	±30s	< 1h ±10%	120s×0.10=12s
2	5min 300s)	±30s		(300s-120s)×0.10=18s
3	10min 600s)	±30s		(600s-300s)×0.10=30s
4	20min	±1min		(1200s-600s)×0.10=60s
5	40min	±2min		(40min-20min)×0.10=2min
6	1h (60min)	±2min		(60min-40min)×0.10=2min
7	2h (120min)	±3min	≧1h (±5%)	(120min-60min)×0.05=3min
8	3h (180min)	±3min		(180min-120min)×0.05=3min
9	4h (240min)	±3min		(240min-180min)×0.05=3min
10	6h	±5min		(6h-4h)×0.05=0.1h×60=6min
11	8h	±5min		(8h-6h)×0.05=0.1h×60=6min

2. INTRODUCTION

2.1 Study Rationale

This Phase I study will be conducted to explore the dose regimen in humans and to evaluate the safety, tolerability, pharmacokinetics (PK), and receptor occupancy (RO) of meplazumab in healthy subjects, thus providing a new macromolecule antibody drug for the treatment COVID-19 pneumonia.

2.2 Background

Coronavirus is an enveloped positive-stranded RNA virus belonging to the family *Cornonaviridae*, order *Nidovirales*.[1] Over the past 20 years, two human coronaviruses, SARS-CoV and MERS-CoV, were reported to lead to severe and fatal lower respiratory tract infection.[2,3] In Dec 2019, an outbreak of a respiratory syndrome, COVID-19, was detected in China, which is caused by a novel coronavirus named SARS-CoV-2.[4] The genome sequence of SARS-CoV-2 shows it belongs to betacoronavirus genus and has extremely high homology with SARS-CoV at genome and proteome.[5,6]

Illness onset among rapidly increasing numbers of COVID-19 in China and the globe indicates that SARS-CoV-2 is more contagious than both SARS-CoV and MERS-CoV. The infection of SARS-CoV-2 leads to acute viral exudative pneumonia, with multiple organ damages, especially in the lung, presenting bilateral diffuse alveolar damage with cellular fibromyxoid exudates.[7] 80% of cases have mild symptoms (including non-pneumonia and mild pneumonia cases), while about 20% patients have developed severe pneumonia and acute respiratory distress syndrome, which attribute to death.[8] As of April 8th, 2020, 1,282,931 confirmed cases and 72,774 death had been reported globally in over 211 countries and areas.[9]

2.2.1. Meplazumab, a Humanized Anti-CD147 IgG2 Monoclonal Antibody

2.2.1.1 Description

Meplazumab (Ketantin®) is a lyophilized powder for injection of small volume. The main active ingredient of the product, meplazumab, is a humanized immunoglobulin (Ig) IgG_2 mAb, consisting of the complementary-determining regions of anti-CD147 murine antibody and the human framework region. Meplazumab for Injection is a novel humanized mAb, which was developed by the National Translational Science Center for Molecular Medicine and Jiangsu Pacific Meinuoke Biopharmaceutical Co. LTD. Meplazumab is a recombinant human IgG_2 antibody expressed by CHO cells. We use bioinformatics and recombinant technology to replace the FR sequence in the light and

heavy chain variable regions of the antibody with the human FR sequence. More than 2/3 of the whole molecule is a human source, in which the variable region has the function of binding antigen, while the constant region has the function of antibody effect, immunogenicity, and species characteristics. The Fc fragment of the chimeric antibody can prolong the half-life of the antibody in serum and theoretically reduce the immunogen of the heterologous antibody. Compared with its parent non-humanized MAb, the equilibrium dissociation constant of humanized antibody did not change significantly, and the affinity constant (K_D) was 1.7×10^{-10}M, which indicated that meplazumab and its parent mouse MAb 6H8 (affinity constant $K_D = 4.48 \times 10^{-10}$M) had a similar affinity, which ensured the binding ability of MAb to target molecules *in vivo* and *in vitro*.

2.2.1.2 Structure

Meplazumab is produced in Chinese hamster ovary cells using recombinant deoxyribonucleic acid (rDNA) technology. It has a typical antibody structure composed of 2 light chains and 2 heavy chains linked by interchain disulfide bonds. Each light chain comprises 214 amino acids and 2 intrachain disulfide bonds. Each heavy chain comprises 442 amino acids and 4 intrachain disulfide bonds.

2.2.2 Preclinical Data

Preclinical studies and pharmacological studies have indicated that meplazumab for intravenous injection is safe and effective with well-controlled quality. The nonclinical studies of meplazumab include pharmacology, PK, and toxicology studies. Refer to Section 4 of the current IB for meplazumab for further details.

2.2.3 Pharmacology

In pharmacology studies, meplazumab's affinity to human- and nonhuman primate-derived CD147 and the mechanism of action of meplazumab was evaluated through an array of in vitro studies. Meplazumab affinity to non-human primate CD147 antigen was approximately 10-fold lower (1.26×10^{-9} K_D in cynomolgus and 2.16×10^{-9} K_D in rhesus monkey) when compared with affinity to the human CD147 antigen. In vitro studies also support the mechanism of action of meplazumab. The concentrations of meplazumab at human CD147 RO% of 10%, 20%, 50%, and 90% were calculated to be 0.511, 0.820, 1.634, and 4.169 μg/mL, respectively. When the drug exhibited intravascular distribution, the drug dose at receptor occupancy level of 10%, 20%, 50%, and 90% at maximum concentration (C_{max}) was predicted to be 0.039, 0.063, 0.126, and 0.321 mg/kg, respectively. After in vitro incubation of meplazumab with rhesus monkey peripheral blood, the concentration of meplazumab at CD147 RO% of 10%, 20%, 50%, and 90%

were calculated to be 1.280, 3.268, 12.264, and 51.057 μg/mL, respectively. The drug dose at RO% of 10%, 20%, 50%, and 90% at C_{max} were predicted to be 0.079, 0.203, 0.762, and 3.171 mg/kg, respectively.

The binding efficacy of CD147 to SARS-CoV-2 spike (RBD) protein (SP) was evaluated by SPR, Co-Immunoprecipitation (Co-IP), and ELISA. The interaction between CD147 and SP was validated, the affinity constant (K_D) of CD147 to bind to SP is 1.85 $\times 10^{-7}$ M, and half-maximal effect concentration (EC_{50}) was determined to be 68.83 µg/mL. The subcellular localization of CD147 and SP was observed in samples from SARS-CoV-2 virus-infected Vero E6 cells by Colloidal gold particles in double-labeling immunoelectron microscopy.

The binding efficacy of CD147 to CyPA was evaluated by SPR, Co-Immunoprecipitation (Co-IP), and ELISA. The interaction between CD147 and CyPA was validated, the affinity constant (K_D) of CD147 to bind to CyPA was 3.34 $\times 10^{-8}$ M, and half-maximal effect concentration (EC_{50}) was determined to be 135 µg/mL.

meplazumab was found to block the interaction between CD147 and its ligands, SP and CyPA, with a half-maximal inhibitory concentration (IC_{50}) of 16.44 μg/mL and 1.28 μg /mL, respectively.

In vitro, functional studies evaluating the inhibitory effects of meplazumab on Vero E6 cells infected with SARS-CoV-2 (2019-nCoV/Beijing/AMMS01/2020) virus strain demonstrated that meplazumab effectively inhibited the infection of virus strain in a concentration-dependent manner. The median effective concentration (EC_{50}) for cytopathic effect(CPE) was 35.98 μg/mL, and the median inhibitive concentration (IC_{50}) for virus gene copy number was 17.58 μg/mL, respectively.

The effect of meplazumab binding to CD147 and the oxygen-carrying/release properties of erythrocytes were also explored in vitro. meplazumab at concentrations up to 25 µg/mL, did not affect the oxygen-carrying/release capacity of erythrocytes in vitro. However, meplazumab may promote the release of oxygen at concentrations ranging from 125 to 2000 µg/mL.

2.2.4 Pharmacokinetics

Meplazumab PK was evaluated in rhesus monkeys and as part of the 4-week Good Laboratory Practice (GLP) toxicology studies performed in both cynomolgus and rhesus monkeys. Following single intravenous doses of 0.3, 1.0, and 3.0 mg/kg meplazumab in rhesus monkeys, meplazumab exposure increased greater than proportional with increasing dose. The half-life time ($t_{1/2}$) was 143±51.2h, 141±36.0h, and 162±48.6h,

respectively. After repeated intravenous (IV) injections with meplazumab at 1 mg/kg once weekly for 4 weeks, meplazumab did not accumulate. There was no difference between genders in meplazumab PK. Anti-drug antibody (ADA) formation following a single IV dose of meplazumab did not appear to meaningfully affect exposure; however, given the estimated half-life under single-dose conditions, lack of accumulation following once-weekly dosing is likely a result of ADA formation after repeated dosing. Furthermore, tissue distribution and excretion of ^{125}I-meplazumab was studied in Sprague Dawley rats. At 240 hours after administration, the radiation concentration of ^{125}I-meplazumab was mainly distributed in plasma, whole blood, and hemocytes. meplazumab did not pass through the blood-brain barrier and did not accumulate in skeletal muscle tissue. The ^{125}I-meplazumab metabolites were mainly excreted by the urine, and a small portion was eliminated in the feces.

2.2.5 Toxicology

Single-dose GLP (cynomolgus monkey) and 4-week GLP (cynomolgus and rhesus monkey) studies were conducted to evaluate the in vivo safety of meplazumab in nonhuman primates. Treatment with a single dose of meplazumab at up to 100 mg/kg did not cause deaths in cynomolgus monkeys. No obviously drug-related toxic effect was observed. The no observed adverse effect level (NOAEL) was considered to be 100 mg/kg in this study. In the repeat-dose study of meplazumab at doses of 2, 6, and 12 mg/kg, the significant changes of meplazumab on cynomolgus monkeys included: increased percentage reticulocyte count at ≥6 mg/kg doses, considered to be a pharmacodynamic effect. The above alterations were reversible. Under the conditions of this study, the NOAEL was considered to be at 12 mg/kg with corresponding $AUC_{(0-168)}$ of 19 123 g·h/mL in males and 11 830 g·h/mL in females.

Intravenous administration of meplazumab once weekly in rhesus monkeys for 4 weeks at doses of 4 or 20 mg/kg/week was well tolerated. The meplazumab-related changes were present in erythrocyte mass parameters (erythrocyte counts, hemoglobin, and hematocrit [HCT]) decreases, reticulocyte increases, and total bilirubin (TBIL) and direct bilirubin (DBIL) increases. When given 20 mg/kg/week meplazumab, the changes of erythrocyte mass parameters, TBIL and DBIL were considered to be toxicologically significant. The NOAEL was considered to be 4 mg/kg with a corresponding $AUC_{(0-168)}$ of 2894 μg·h/mL in males and 3420 μg·h/mL in females.

Supporting in vitro tissue distribution, hemolysis, and antibody-dependent cell-mediated cytotoxicity assays complement the toxicology program. Meplazumab at the maximal

intended clinical concentration of 2 mg/mL did not cause hemolysis or erythrocyte aggregation in rabbit, cynomolgus monkey, or human erythrocytes in vitro and was deemed suitable for injection in the clinic. Meplazumab over the concentration range of 0.0125 to 1 mg/mL did not induce an antibody-dependent cell-mediated cytotoxicity effect in vitro.

2.2.6 Clinical Data

The therapeutic effects of meplazumab against COVID-19 were investigated in an exploratory clinical study. A prospective, single-center, open-labeled trial at Tangdu Hospital of Fourth Military Medical University in Xi'an, China. The study protocol and consent were approved by the Independent Ethics Committee of Institution for National Drug Clinical Trials at the Tangdu hospital. The study was registered at ClinicalTrials.gov (NCT04275245) before any patient enrollment.

Enrolled patients fulfilled inclusion and exclusion criteria. The inclusion criteria are as follows: men and women aged 18 to 78 years; patients with common, severe, or critical COVID-19 pneumonia were laboratory and clinically diagnosed according to *Diagnosis and Treatment for 2019 Novel Coronavirus Diseases* released by National Health Commission of China;[18] the subjects must understand the study and be willing to participate in the study. The exclusion criteria are as follows: allergic reactions or a history of allergy to any of the ingredients treated in this trial; patients not suitable to participate in this study by the judgment of the investigator. Each patient signed an informed consent form before enrollment.

Case severity of COVID-19 is categorized as common, severe, and critical by the vital signs, oxygenation index (PaO_2/FiO_2), chest radiographic, and vital organ function based on the *Diagnosis and Treatment for 2019 Novel Coronavirus Disease*.[18] Common case was featured by fever, respiratory symptoms, and radiographic pneumonia. A severe case was characterized by any of the following signs: dyspnea, respiratory frequency ≥30/minute, blood oxygen saturation ≤93%, and PaO_2/FiO_2 ratio <300mmHg. The critical case was characterized by any of the following signs: respiratory failure needing mechanical ventilation, shock, and multiple organ dysfunction/failure needing Intensive Care Unit (ICU). The patients who met all the following criteria will be discharged: body temperature recovered and remained normal more than 3 days; respiratory symptoms relief, and two continuous negatives for nasopharyngeal swab test for SARS-CoV-2 (interval more than 24 hours).

Seventeen patients with COVID-19 pneumonia were administrated with meplazumab

treatment. 10mg meplazumab was administered on day 1, day 2, and day 5 by intravenous infusion within 60–90 min. Efficacy and safety were assessed at baseline, every day after day 1 to day 14, and every week thereafter up to day 28 or discharge.

In the same period, hospitalized patients in the same center were observed as concurrent control and were required to follow the inclusion and exclusion criteria mentioned above.

- Recovery and Discharged Meplazumab treatment improved the discharged rate of severe and critical cases significantly compared to the control group (p=0.005). No discharged cases were observed over the follow-up period in the control group. On day 28, 4 severe cases and 1 critical case were improved to common, and no case was discharged in the control group. In the meplazumab group, 9 cases (6 severe and 3 critical) were discharged, 2 critical cases were improved to common, and 1 critical case was improved to severe, demonstrating a significantly beneficial outcome compared to the control group (p=0.021). These results indicated that meplazumab treatment accelerated the improvement and made a rapid recovery from COVID-19 pneumonia, especially for the severe and critical cases.
- The chest radiographic The meplazumab group showed more significant benefit than the control group on days 7, 14, and 21 (p=0.010, p=0.006, and p=0.037, respectively), which was coincident with the improvement of case severity.
- The virus clearance At day 7, the rate of virus nucleic acid negative conversion in the control group was 27.3% (3/11) and reached 54.4% (6/11) on day 14. While the rate was 76.5% (13/17) in the meplazumab group on day 7 and reached 94.1% (16/17) on day 14, which were significantly higher than the control group (p=0.019 and p=0.022, respectively). The analysis of time to virus-negative indicated that meplazumab-treated patients converted to negative in a shorter period than patients in the control group significantly (median 3, 95%CI [1.5–4.5] vs. 13, [6.5–19.5]; p=0.045, HR=0.374, 95%CI [0.143–0.978]).
- Lymphocytopenia count In the observation period (day 7 to 28), the percentages of patients with a normal lymphocyte count ($>0.8\times10^9$/L) were increased in both groups, while the improvement in the meplazumab group was more notable. Compared to the baseline, the percentage in the meplazumab group was improved significantly as early as day 7 (p=0.031), but no

significant difference was detected in the control group over the follow-up period.

- CRP The concentration of CRP was also measured as a predictor of COVID-19 severity. From day 3 to day 28, the percentages of patients with a normal CRP concentration (≤5mg/L) were increased from 52.9% (9/17) to 82.4% (14/17), indicating significant increases compared to baseline (all $p<0.05$ at days 3, 7, 14, 21, and 28). In the control group, significant increases were observed on day 14 and day 21 compared to baseline. The data suggest that meplazumab exhibited an effect on the control of virus-induced acute inflammation at early management.
- Safety and tolerance No other adverse events were reported in meplazumab-treated patients, including irritation around the injection site, rash, nausea, vomit, anemia, neutropenia, thrombocytopenia, total bilirubin, albumin, and creatinine, etc.

All above, meplazumab efficiently improved the recovery of patients with SARS-CoV-2 pneumonia with a favorable safety profile.

2.3 Benefit/Risk Assessment

As this study will be conducted on healthy human subjects, a health benefit to the subject is not anticipated.

The clinical study protocol has been designed such that the risk to subjects in this study will be minimized by adequate selection of eligibility criteria and schedule of clinical monitoring, in-house observation, administration, and treatment duration. The Sponsor will immediately notify the Principal Investigator if any additional safety or toxicology information becomes available during the study.

This study will be performed in compliance with the protocol, International Council for Harmonisation (ICH) Good Clinical Practice, and applicable regulatory requirements.

2.4 Meplazumab

Based on the nonclinical safety profile and the expected high efficacy against COVID-19 pneumonia, it is anticipated that meplazumab will have a positive benefit-risk profile to warrant studies in healthy volunteers if conducted under well-controlled conditions, including in-house confinement periods, extensive monitoring, and protective measures for contraception.

Meplazumab has not yet been evaluated in humans in controlled clinical studies. Thus, information regarding expected adverse events (AEs) from controlled clinical studies is unknown. Based on the available nonclinical data to date, meplazumab did not cause serious toxicity and side-effects on the tested animals. Therefore, the conduct of the study is considered justifiable.

Based on the nonclinical data, possible adverse reactions from meplazumab treatment include:

- Changes in erythrocyte mass parameters
- Increases in TBIL, DBIL, and alanine aminotransferase (ALT)
- Possible off-target cross-reactivity on some of the CD147 on other organs

Potential risks of a foreign protein such as a therapeutic antibody may include the administration or immune reactions, including hypersensitivity, injection-site reactions, and immunogenicity. Subjects could potentially develop ADAs that may be neutralizing and may be associated with allergic or anaphylactic toxicity or induce or enhance meplazumab toxicity. Standard clinical assessments and interventions are recommended for allergic or anaphylactic reactions. Subjects will be monitored for these events and ADA formation in this first-in-human (FIH) study.

More detailed information about the expected benefits and risks and reasonably expected AEs of meplazumab may be found in the IB.

3 OBJECTIVES AND ENDPOINTS

Table 5 Study Objectives and Endpoints

**Objectives**	**Endpoints**
Primary	
• To evaluate the safety, and tolerability of single ascending doses and multiple doses of meplazumab in healthy subjects.	For subjects who have received a single dose and multiple doses of meplazumab or placebo: • Nature, incidence, and severity of AEs/SAEs, including relationship to study treatment, AEs/SAEs leading to discontinuation of the study
Secondary	
• To evaluate meplazumab PK in blood following single-dose and multiple dose administration.	• Meplazumab blood concentration-time profiles and calculated PK parameters including but not limited to $AUC_{(0\text{-}t)}$, $AUC_{(0\text{-}\infty)}$, and $T_{½}$ etc.
• To evaluate receptor occupancy of meplazumab in peripheral blood cells	• Rate of receptor occupancy in peripheral blood cells after the administration of meplazumab
• To evaluate laboratory examination of meplazumab	• Laboratory examination before and after the administration of meplazumab

AE = adverse event; $AUC_{(0\text{-}\infty)}$ = area under the concentration-time curve extrapolated to infinity; $AUC_{(0\text{-}t)}$ = area under the concentration-time curve to the t; PK = pharmacokinetic; RO = receptor occupancy; SAE = serious adverse event; $T_{½}$ = elimination half-life.

4 STUDY DESIGN

4.1. Overall Design

This Phase I FIH study is assessing safety, tolerability, PK, and RO% of meplazumab in healthy subjects following escalating single and multiple intravenous infusion. It is a single-center, randomized, double-blind, placebo-controlled, dose-escalation study. Seven cohorts are planned, and each cohort will consist of 8 or more subjects with 6 or more subjects receiving meplazumab and 2 subjects receiving placebo, for a total of 56 or more subjects. No subject will be a member of more than 1 cohort in the study.

The Treatment Emergent Adverse Events (TEAEs) will be observed, and the pharmacokinetic (PK) will be evaluated during the study period. Blinded safety data including the nature and severity of AEs, infusion-site reactions, ECGs, vital signs, findings from physical examination, as well as hematology and biochemistry results will be reviewed by the investigator, after which dosing in the remaining 6 subjects may commence provided there are no safety or tolerability concerns.

In single-dose study, 6 cohorts, 0.06mg / kg, 0.12mg / kg, 0.2mg / kg, 0.3mg / kg, 0.42mg / kg, 0.56mg / kg, respectively, were planned. At least 8 subjects will be enrolled in each cohort; 6 will be administrated meplazumab, and 2 with placebo. If no adverse events were observed in the highest dose group, and the PK results suggest that it has no saturation, the sponsor and the investigator could add an extra dose group to investigate the single-dose PK. In multiple-dose study: the dosage and frequency of administrations should be adjusted according to the results of the pilot study and single-dose PK study.

In single-dose PK study: subjects will be screened from Day -14 to Day -2, the information of general condition, medical and medication history, vital signs, physical examination, laboratory examination (blood routine, urine routine, hepatic and renal function, coagulation function), ECG, drug abuse, and other related examinations of subjects will be collected. Subjects who meet the inclusion criteria will be admitted to the study center on Day -1 and be confined to the study center under medical supervision. Subjects should light diet at night and fast (no food or drinks other than water) for at least 10 hours (overnight). On the first day (Day 0), a single dose of meplazumab or placebo will be administered as an intravenous infusion over 60 minutes in the morning on an empty stomach. Subjects will be confined to the clinic until the morning of Day 7, and blood samples were collected according to the schedule of assessments at multiple time points until 168h after the administration. Subjects will return to the study center

for follow-up visits on Days 10 (240h), 14 (336h), 21 (504h), 28 (672h), 38(912h), 48(1152h), 56 (1344h), and 84 (2016h) for safety, tolerability, PK, RO%, and immunogenicity assessments. On day 8-9, Day 11-13, Day 15-20, Day 22-27, Day 29-37, Day39-47, Day49-55, Day 57-83, the safety evaluation will be completed by telephone follow-up. (Table 1).

In multiple-dose study: subjects will be screened from Day -14 to Day -2, the information of general condition, medical history, medication history, vital signs, physical examination, laboratory examination (blood routine, urine routine, hepatic and renal function, coagulation function), ECG, drug abuse, and other related examinations of subjects will be collected. Subjects who meet the inclusion criteria will be admitted to the study center on Day -1 and be confined to the study center under medical supervision. Subjects should light diet at night and fast (no food or drinks other than water) for at least 10 hours (overnight). On the first day (Day 0), the first dose of meplazumab or placebo will be administered on Day 1 as an intravenous infusion over 60 minutes in the morning on an empty stomach. The second dose of meplazumab or placebo will be administered on Day 7 as an intravenous infusion over 60 minutes in the morning on an empty stomach. Subjects will be confined to the clinic until the morning of Day 7 after the second administration, and blood samples were collected according to the schedule of assessments at multiple time points until 168h after the second administration. Subjects will return to the study center for follow-up visits on Days 10 (240h), 14 (336h), 21 (504h), 28 (672h), 38(912h), 48(1152h), 56 (1344h), and 84 (2016h) after second administration for safety, tolerability, PK, RO%, and immunogenicity assessments. On day 8 - 9, Day 11 - 13, Day 15 - 20, Day 22 - 27, Day 29-37, Day39-47, Day49-55, Day 57 - 83 after the second administration, the safety evaluation will be completed by telephone follow-up (Table 1).

The safety evaluation should be performed during the screening period, baseline period, observation period, and before out of the group. The investigator can increase the safety check according to the actual situation. All abnormal and clinically significant changes of the subjects need to be followed up until they return to normal, or the investigator believes that there is no clinical significance.

Aspects of study design that may be adapted are presented in Table 6.

Table 6 Adaptive features in study

Category	Adaptive features	Boundaries
Dose levels	Following a starting dose of 0.065 mg/kg, planned doses for Cohorts 2 to 6 are: 0.12 mg/kg, 0.2 mg/kg, 0.3 mg/kg, 0.42mg/kg and 0.56 mg/kg. These dose levels are tentative only and subject to change.<br>If no adverse events were observed in the highest dose group, and the PK results suggest that it has no saturation, the sponsor and the investigator could add an extra dose group to investigate the single-dose PK.<br>For multiple-dose study: the dosage and frequency of administrations should be adjusted according to the results of the pilot study and single-dose study. The initially set dosage is 0.3mg/kg.	Maximum dose will not exceed the predicted exposures equivalent to exposure limits observed at the NOAEL in rhesus monkeys (females): C_{max}: 80 µg/mL; $AUC_{(0-168)}$: 2894 µg·h/mL<br>Dose escalation will be stopped if stopping criteria are met.
Sample size	The number of cohorts may decrease if dose escalation is stopped.	Minimum sample size – 56 subjects
Study schedule	Confinement period may be extended beyond Day 7 at the Investigator's discretion based on emerging safety & PK data<br>The time to EOS (currently Day 84±2) may be extended at the Investigator's discretion to allow for appropriate assessment of safety, PK, and RO data.	Confinement will not exceed Day 7, unless medically indicated.<br>The maximum window to EOS visit will not exceed Day 84 (±2 days), unless medically indicated.
Assessments	Timing of PK and RO blood sampling may be adjusted based on emergent PK and/or RO results.	In accordance with evolving data up to the decision-making time point.
	Additional safety blood and/or urine samples may be taken if it is necessary from a safety/tolerability point for an upcoming dose cohort.<br>At the Investigator's discretion, additional or fewer exploratory assessment samples may be taken in accordance with emerging data.	The maximum volume of blood collected will not exceed 270 mL in a 30-day period.<br>Efforts will be taken to ensure the subject's safety is not compromised.

AUC = area under the concentration-time curve; C_{max} = maximum concentration, obtained directly from the observed concentration-versus-time data; EOS = end of study; NOAEL = no observed adverse effect level; PK = pharmacokinetics.

4.2. Scientific Rationale for Study Design

This is a Single-center, placebo-controlled, dose-escalation phase I clinical trial of meplazumab in healthy volunteers. The rationale for the various aspects of this study design is described below. The justification for the meplazumab dose is provided in Section 4.6.

4.3. Study Population

While the intended patient population for meplazumab is people infected with COVID-19 pneumonia, this FIH study will be performed in healthy subjects to characterize the safety, tolerability, and PK of the compound in humans in the absence of any disease-related and potentially confounding factors. The inclusion criteria were chosen to help ensure the inclusion of only healthy subjects. Additional criteria are in place to ensure the safety and wellbeing of subjects.

4.4. Safety Assessments

All safety analyses will be performed on the Safety Analysis Set. Adverse events will be coded using the Medical Dictionary for Regulatory Activities. The overall number and percentage of subjects with at least one AE (and SAE) will be tabulated over the entire study period. Overall, AE data will be summarized by treatment received and study period. For each study treatment and study period, the frequency of all treatment-emergent AEs (TEAEs), TEAEs by maximum severity, TEAEs by relationship to study treatment, SAEs, TEAEs leading to death, and TEAEs leading to discontinuation of the study will be tabulated by preferred term and system organ class for each treatment group.

4.5. Study Design

In the study, a stepwise dose escalation will be used to allow evaluation of the safety of meplazumab with minimal risk to subjects.

The use of a placebo control will help distinguish any potential effects between meplazumab itself and other effects not related to meplazumab with minimal bias. A 3:1 ratio of meplazumab versus placebo is an accepted standard used in FIH studies.

As per common practice for Phase 1 studies, subjects will remain confined to the study center after dosing with meplazumab or placebo. A 7-day post-dose confinement (up to

Day 7) is expected to cover 1 meplazumab elimination half-life (t½) based on the transformation of nonhuman primate (rhesus monkey and cynomolgus monkey) concentration-time profiles to human concentration-time profiles using the species-invariant time method. Thereafter, subjects will have regular outpatient visits every alternate day up to Day 84 to assess for safety; all efforts will be made by the study site to regularly contact subjects (approximately every 7 days) by phone call or text message in between visits during their ambulant period.

Receptor occupancy assays will be used to explore the relationship between meplazumab concentration and receptor occupancy rate and will support the determination of the dose required to maintain pharmacologically active exposure levels of meplazumab for phase II. Receptor occupancy (optional) assays will also be performed.

4.6. Justification for Dose of Meplazumab

The starting dose in phase I clinical trial was selected considering the results of the receptor occupancy analysis, non-clinical pharmacokinetics, toxicology, and non-registered clinical studies, combined with the administration method and mechanism of the drug, and the safety of human administration.

The mechanism of action of this product is to inhibit virus invasion into host cells by blocking CD147-spike protein interaction and to inhibit inflammatory storm syndrome response by blocking CD147 - CyPA interaction.

In the single-dose GLP toxicology study of cynomolgus monkeys, the no observed adverse effect level (NOAEL) was 100 mg/kg. In repeated toxicity studies, meplazumab was NOAEL 4 mg/kg in rhesus monkeys and 12 mg/kg in cynomolgus monkeys. Combined with the above results, we determined that NOAEL of meplazumab was 4 mg/kg. According to the relevant FDA guidelines for the calculation of Human Equivalent Dose (HED), the tolerated dose for human use should not exceed 4 mg/kg, and the starting dose for human use should be 0.4 mg/kg if the safety factor is set at 10.

The results of peripheral blood receptor occupancy (RO) analysis suggested that when RO was 90%, the estimated antibody dosage was 0.321 mg/kg. The minimum anticipated biologic effect level (MABEL) is about 0.3 mg/kg, assuming that antibodies bind to the virus-host cells only when the blood cells are fully bound, regardless of the shedding of antibodies bound to the blood cells. At this dose, the half-life of meplazumab in rhesus monkeys was 143±51.2 h, and AUC_{INF_obs} was 126±22.6h·μg/mL.

In the exploratory clinical study, the total antibody dosage for the subject was 30 mg, and the bodyweight was 60 to 75 kg, which was about 0.4 to 0.5 mg/kg.

From a safety perspective, a lower Starting Dose of 0.06mg/kg should be used, taking into account the differences between the unregistered clinical study regimen and the phase I clinical study regimen and the long antibody half-life, 0.56 mg/kg was selected as the Maximum Recommended Starting Dose (MRSD) for clinical trials. The proposed 0.15 mg/kg starting dose is 1/26.7 of the NOAEL in rhesus monkeys (4 mg/kg/week), which is the lowest NOAEL determined based on weight-equivalent scaling between two 4-week toxicology studies in rhesus and cynomolgus monkeys. The rhesus monkey NOAEL is associated with a total exposure over a 1-week dosing interval ($AUC_{[0-168]}$) of 2894 μg·h/mL in males and 3420 μg·h/mL in females. The C_{max} at 4 mg/kg following once-weekly dosings was 90 μg/mL in males and 80 μg/mL in females.

The 0.15 mg/kg dose targets a meplazumab C_{max} with a potential CD147 receptor occupancy of ~50%. A difference was observed for the antigen affinity of meplazumab between human and non-human primates (human $K_D = 1.7\times10^{-10}$; rhesus $K_D = 2.16\times10^{-9}$; cynomolgus $K_D = 1.26\times10^{-9}$). The impact of this difference on erythrocyte CD147 receptor occupancy by meplazumab, considered as an indicator of pharmacological activity, was explored.

The tentative planned doses for Cohorts 2 to 5 are: 0.3 (100% increase), 0.5 (67% increase), 0.75 (50% increase), and 1 (33% increase) mg/kg. The highest meplazumab dose will not exceed the predicted exposures equivalent to exposure limits observed at the NOAEL in rhesus monkeys (females): C_{max}: 80 μg/mL; $AUC_{(0-168)}$: 2894 μg·h/mL. If the predefined exposure limits are reached, and there are no safety or tolerability concerns, a lower dose may be investigated.

4.7. End of Study Definition

The EOS is defined as the date of the last visit of the last subject in the study. The sponsor will notify all study units of the end time of the study. After this time, the study must be approved by the sponsor and can be implemented without supplement.

4.8. Study Stopping Criteria

The sponsor reserves the right to terminate the study at any time. The study will be stopped include but not limited to:

- The investigator cannot follow the trial protocol or GCP guidelines;
- Any security concern;
- Evidence to suggest a lack of therapeutic effect;
- The investigator did not recruit enough subjects.

If any of the criteria for stopping dose escalation are met in any cohort of study, the study will be stopped.

5 STUDY POPULATION

5.1.Inclusion Criteria

Subjects are eligible to be included in the study only if ALL of the following criteria apply:

1. 18≤age≤50 years, males or females;
2. body weight ≥50 kg, body mass index (BMI) should be within 19.0 and 26.0 (both ends included);
3. Vital signs, physical examination, laboratory examination (blood routine, urine routine, blood biochemical, coagulation, etc.), and electrocardiogram are within the normal range or beyond the normal range, but the researchers determined that the abnormality has no clinical significance (NCS);
4. No bad habits, including tobacco addiction (>5 cigarettes per day) or drink addiction (>15 g of alcohol in a day, and more than two days a week for female, or >25 g in a day, and more than two days a week for male; 15 g of alcohol is equivalent to 450 mL beer, 150 mL wine or 50 mL light liquor, no history of drug abuse (defined as the use of illegal drugs);
5. No birth plan during the study and within 6 months of completing the test and are willing to use non-hormonal contraceptive measures;
6. Have the ability to normally communicate with medical staff and comply with relevant hospital management regulations;
7. Capable of giving signed informed consent, which includes compliance with the requirements and restrictions listed in the informed consent form (ICF) and in this protocol, prior to any study-specific procedures.

5.2.Exclusion Criteria

Subjects are excluded from the study if ANY of the following criteria apply:

- Allergic reactions or a history of allergy to any of the ingredients treated in this trial.
- Vital signs, physical examination, routine laboratory tests (blood routine, urine routine, blood biochemistry, coagulation, etc.), 12-lead ECG and other abnormalities and clinical significance;
- Positive for SARS-CoV-2 specific IgM and IgG antibodies test;
- Fever within 3 days before medication (body temperature ≥38.0 ℃);
- Pregnant or breastfeeding;

- Have received or are participating in other clinical trials within 3 months before the screening;
- Be diagnosed or suspected to have immunodeficiency or autoimmune diseases; undergo immunosuppressive therapy such as anticancer chemotherapy or radiotherapy before the trial, or have received systemic corticosteroid treatment within the past 6 months;
- With a history of acupuncture syncope reaction;
- Positive for HBV surface antigen, anti-HCV antibody, anti-HIV antibody, and syphilis antibody test;
- Tobacco addiction (>5 cigarettes per day) or drink addiction (>15 g of alcohol in a day, and more than two days a week for female, or >25 g in a day, and more than two days a week for male; 15 g of alcohol is equivalent to 450 mL beer, 150 mL wine or 50 mL light liquor, or can not stop smoking and drinking during the study;
- Participated in blood donation or blood loss ≥400mL within 3 months before screening;
- Patients not suitable to participate in this study by the judgment of the investigator.

5.3.Enrollment

Approximately 56 or more healthy men and women are planned and will assigned to 7 cohorts. Subjects will be recruited from the clinical site database or by a general or study-specific advertisement via print, radio, or poster media to the general community, as approved by the independent ethics committee. No restrictions will apply to ethnic or racial categories.

Subjects will be compensated for the time that they spend participating in the study using a formula determined by the study site.

Screen Failures

Screen failures are defined as subjects who consent to participate in the clinical study but who do not meet the eligibility criteria and are not subsequently entered in the study. A minimal set of screen failure information is required to ensure transparent reporting of screen failure subjects to meet the Consolidated Standards of Reporting Trials publishing requirements and to respond to queries from Regulatory Authorities. Minimal information includes ICF and Screening Visit dates, demography, screen failure details, eligibility criteria, and any SAE.

6 STUDY TREATMENT

Study treatment is defined as any investigational treatment(s), non-investigational treatments, marketed product(s), placebo, or medical device(s) intended to be administered to a subject according to the study protocol. All study treatments will be packaged and labeled in accordance with all applicable regulatory requirements, including the Access to Unapproved Therapeutic Goods.

6.1.Study Treatment(s) Administered

meplazumab for injection will be provided in the form of a 10-mg/vial freeze-dried powder containing 1.60 g histidine, 3.08 g histidine hydrochloride, 50.0 g sucrose, 70.0 g mannitol, and 1.0 g polysorbate 80.

A matching placebo will be provided in the form of a 10-mg/vial freeze-dried powder containing 1.60 g histidine, 3.08 g histidine hydrochloride, 50.0 g sucrose, 70.0 g mannitol, and 1.0 g polysorbate 80 for IV infusion.

Meplazumab and placebo will be provided by the Sponsor.

6.2.Preparation/Handling/Storage/Accountability

6.2.1 Preparation, Dosage, and Administration

Meplazumab

The vial of meplazumab will be reconstituted with 1 mL of water for injection. The required amount of drug solution will be withdrawn and added to 100 mL sterile normal saline (0.9%) for IV infusion. The time between preparation of meplazumab and administration to each subject should be a maximum of 2 hours (maintained at room temperature).

A single dose of meplazumab will be infused over 60 minutes at a constant rate using an infusion pump. The subject’s weight on Day -1 or Day 1 pre-IMP will be used to calculate the dose of meplazumab.

6.2.2 Handling, Storage, and Accountability

1. On receipt of the study treatment, the investigator or designee must confirm appropriate temperature conditions have been maintained during transit for all study treatment received, and any discrepancies are reported and resolved before use of the study treatment.
2. Only subjects enrolled in the study may receive study treatment, and only authorized study center staff may supply or administer study treatment. All study treatments must be stored in a secure, environmentally controlled, and monitored (manual or

automated) area in accordance with the labeled storage conditions with access limited to the Investigator and authorized study center staff.

3. The Investigator, institution, or the head of the medical institution (where applicable) is responsible for study treatment accountability, reconciliation, and record maintenance (i.e., receipt, reconciliation, and final disposition records).
4. All products will be inventoried upon receipt by the clinical study site pharmacist or delegate. The condition of the products at the time of receipt by the pharmacist will be documented. The lot numbers and expiry dates of the challenge agent will be documented. The clinical study site pharmacist or delegate will ensure that the received products are the specified formulation.
5. The storage, handling, and disposal of the agent will be in accordance with approved procedures.
6. Used and unused drug containers must be destroyed at the site once drug accountability is final and has been checked by the Sponsor or its delegate, and written permission for destruction has been obtained from the Sponsor.

The investigator, a member of the study center staff, or a hospital pharmacist must maintain an adequate record of the receipt and distribution of all study treatment using the Drug Accountability Form. These forms must be available for inspection at any time. Meplazumab and placebo should be stored and transported at 5°C ± 3°C, away from light. Once reconstituted, meplazumab is stable at 5°C for up to 24 hours. Once diluted with saline, meplazumab is stable at room temperature for up to 24 hours.

6.3.Measures to Minimize Bias: Randomization and Blinding

Subjects will receive their subject identification number (screening number) as soon as they have signed the informed consent. For each cohort, subjects who meet the eligibility criteria will be randomized in a double-blind fashion to meplazumab or placebo in a ratio of 3:1, respectively.

The computer-generated randomization schedules will be created by an unblinded statistician prior to the study start. On Day -1 or Day 1, subjects will be allocated a randomization number (created as per the Sponsor's standards) in sequential order, immediately before administration of the IMP. A copy of the randomization schedule will be sent to the clinical unit pharmacist and clinical unit project manager.

Investigators will remain blinded to each subject's assigned IMP treatment throughout the course until all final clinical data have been entered into the database and all data queries have been resolved, and the assignment of subjects to the analysis sets has been

completed. In order to maintain this blind, the randomization list is to be kept strictly confidential, accessible only to authorized persons (e.g., randomization statistician, pharmacists, unblinded Clinical Research Associate, and the bioanalytical laboratories that prepare and analyze relevant samples), until the time of unblinding. An unblinded pharmacist at the study center will be responsible for the reconstitution and dispensation of all study treatment and will endeavor to ensure that there are no differences in time taken to dispense following randomization.

The trial drugs, including meplazumab and placebo, were provided by the sponsor, and it was guaranteed that meplazumab injection and placebo were the same in terms of dosage form, size, weight, appearance, etc. For maintaining the blindness of the study, the meplazumab injection solution and the placebo solution cannot be distinguished by naked eyes. Labels for trial drugs and placebos use a uniform format.

Emergency unblinding: In an emergency (when the subject has a serious adverse event or emergency rescue is required), the investigator in charge of the trial institution can decide whether to unblind after consultation with the sponsor urgently. Once the blinding is urgently uncovered, the case will be regarded as a drop-out case and will not be included in the efficacy analysis, but the adverse reactions that occur still need to be included in the safety analysis. If the study drug is unblinded, the investigator must notify the sponsor as soon as possible. The date, time, and reason for the unblinding must be recorded in the source file, and the same information must be recorded in the eCRF.

Unblind: When unblinding, after blind review, the data is locked, and the statistician who keeps the randomization list will unblind. That is, the name of the drug corresponding to each serial number will be notified to the biostatistician so that all data can be statistically analyzed. In the course of clinical trials, if all the blinds are leaked, or the emergency unblind exceeds 20%, this double-blind trial becomes invalid.

Because the major research content of this trial can be completed before Day 56, to speed up the research progress of this trial, the trial can be unblinded in advance if necessary. Unblinding in advance needs to meet the following conditions: (1) All planned subjects have completed Day 56 (56 days after the last dose in the multi-dose group) visit point content; (2) No new treatment-related adverse reactions (TEAE) or clinically significant abnormal was observed or detected in the last visit for all subjects; (3) The sponsor and all major investigators both admit to unblind; (4) the schedule of unblinding were reported to the ethics committee, and the ethics committee agreed to unblind in advance.

6.4.Study Treatment Compliance

The prescribed dosage, timing, and mode of administration of study treatment may not be changed, except as defined in Section 6.6 and in the Schedule of Assessments. Any departures from the intended regimen must be recorded in the eCRF.

meplazumab and placebo will be administered at the clinical unit in the presence of clinical unit staff.

6.4.1 Treatment Strategy

The staff of the study center is responsible for the ongoing safety and wellbeing of the subjects while they are in the study center. There is a paging system to alert the clinical staff to any area in the center where a subject may need medical attention. In the case of an emergency, cardiac resuscitation trolleys are found in the main ward areas of the study center. These trolleys contain drugs, equipment for airway insertion, circulation lines, defibrillation, etc., together with oxygen cylinders and portable suction machines. There is a physician on-site 24 hours a day. In addition, if necessary, the clinical staff can contact further on-call physicians or public emergency services in the event of a serious medical event. Equipment and emergency drugs are available to treat common medical emergencies that might occur in a Phase I study.

6.4.2 Warnings and Precautions

As this is the first administration of meplazumab in humans, all effects cannot be reliably predicted. The preclinical data suggest an acceptable safety margin. Facilities and staff for resuscitation and the treatment of other medical emergencies will be provided.

6.5.Prior and Concomitant Therapy

Prior medications, treatments, and procedures are those occurring prior to IMP dose or inoculation. Concomitant medications from hospitalization to Day 84 will be recorded.

At each visit, subjects will be questioned in relation to their drug intake since their previous visit. Any medication or vaccine (including over the counter or prescription medicines, vitamins, and/or herbal supplements) that the subject is receiving at the time of enrollment or receives during the study must be recorded on the eCRF along with:

- Reason for use.
- Dates of administration including start and end dates.
- Dosage information including exact dose and timing.

The Medical Monitor should be contacted if there are any questions regarding concomitant or prior therapy.

6.5.1 Prohibited Medications

Avoid unnecessary drug treatments that affect the evaluation of study drugs during the study. If serious adverse reactions occur during treatment, they will be dealt with according to relevant clinical diagnosis and treatment guidelines.

6.5.2 Permitted Medications

Based on the premise of protecting the interests and safety of the subjects, the investigator can decide whether to use other concomitant drugs during the study period, which did not affect the evaluation of the study drug. All concomitant medication information and symptomatic treatment received by the subjects were recorded in the eCRF.

6.6.Dose Modification

The details of dose selection are described in Section 4.6, and study stopping criteria in Section 4.8 of this protocol.

6.7.Treatment After the End of the Study

In this study in healthy subjects, no further treatment or medical care is planned or required after the EOS visit.

7 STUDY ASSESSMENTS AND PROCEDURES

7.1 Schedule of Assessments

- For the study, study procedures and their timing are summarized in the Schedule of Assessments.
- Protocol waivers or exemptions are not allowed.
- Immediate safety concerns should be discussed with the Sponsor immediately upon occurrence or awareness to determine if the subject should continue or discontinue study treatment.
- Adherence to the study design requirements, including those specified in the Schedule of Assessments, is essential and required for study conduct.
- At visits where assessment time points coincide with each other, the following procedure should be followed: perform ECGs first, followed by vital signs as close as possible to the scheduled time point but prior to PK and RO% sampling; perform PK and RO% blood sampling at the scheduled time point; perform all other procedures as close as possible to the scheduled time point but may be obtained before or after PK and RO% sampling.
- The maximum amount of blood collected from each subject over the duration of the study, including any extra assessments that may be required, will not exceed 270 mL in any 30-day period. Repeat or unscheduled samples may be taken for safety reasons or for technical issues with the samples.

7.2 Screening Examinations

All subjects will undergo a Screening examination to evaluate their health status. This examination will be conducted not more than 28 days prior to the planned IMP. Only subjects who meet the eligibility criteria will be enrolled in the study.

Written informed consent must be obtained before any study-related procedures and/or assessments are performed. The assessments to be performed at screening are indicated in the Schedule of Assessments. Subjects should have fasted for at least 8 hours prior to the safety laboratory assessments at the screening. Safety laboratory assessments with results outside of the normal laboratory range may be repeated once.

Subjects who fail to meet the protocol-specified inclusion or exclusion criteria or who withdraw their consent in the screening period are considered screening failures. The

investigator will maintain a screening log to record details of all subjects screened and to confirm eligibility or record reasons for screening failure, as applicable.

7.3 Treatment Period

A review and update of the subject's eligibility criteria to ensure the subject remains eligible for participation in the study should be performed on Day -1 on admission to the study site and on Day 1 prior to IMP administration, and will be discharged on the morning of Day 7. Eligible subjects will be randomized to active treatment or placebo.

The confinement periods during the study are subject to change for operational reasons. See Table 4 for details.

The assessments to be performed at specific time points during the treatment period are indicated in the Schedule of Assessment.

7.4 Outpatient Visits and Phone Calls

Ambulatory visits will be performed at regular intervals from Day 12 onwards.

The assessments to be performed at specific time points during outpatient visits are indicated in the Schedule of Assessments.

7.5 End of Study/Early Termination Visit

The EOS visit is to verify that all values tested at screening have remained within a clinically acceptable range. The relevant tests will be performed on Day 84 (± 2 days). The assessments to be performed at this time point are indicated in the Schedule of Assessments. Unacceptable values and AEs will be followed up until they return to baseline/have resolved, or there is an adequate explanation that is not related to the study. The subject may be referred for appropriate counseling or for follow-up tests to a general practitioner or medical specialist as appropriate.

7.6 Demographics and Other Baseline Characteristics

At Screening, the following demographic data will be collected: age, and year of birth, sex (gender), race, and ethnicity.

Furthermore, the following will be documented:

- Social history including recreational drug use, alcohol intake, and tobacco use
- Female status (WOCBP, woman of non-childbearing potential, postmenopausal, sterilization)

7.7 Medical and Surgical History

The medical history will be elicited at Screening as described below. Information regarding any new medical conditions or illnesses will be elicited on Day -1.

Recording of past medical/surgical history will include:

- History of all known allergies
- History of substance abuse and recreational drug use
- History of depression, anxiety, mental illness, emotional problems, use of psychiatric medications, and previous psychotherapy
- Surgical procedures and results
- Any other current or past medical conditions

7.8 Safety Assessments

Planned time points for all safety assessments are provided in the Schedule of Assessments.

7.8.1 Physical Examinations

- A **complete physical examination** will be performed at Screening and the EOS/ET visit and will include, at a minimum, assessments of the general appearance, skin, head, eyes, ears, nose and throat, neck (including thyroid), skin, cardiovascular, respiratory, gastrointestinal, peripheral, musculoskeletal, lymph nodes and nervous systems.
- Pre-IMP dose, an **abbreviated physical examination** will be performed, including, at a minimum, assessments of general appearance, respiratory, cardiovascular, and gastrointestinal systems.
- A **symptom-directed physical examination** will be performed at all other visits if required. Body systems will be reviewed only if clinically indicated at the Investigator’s discretion.
- Investigators should pay special attention to clinical signs related to previous serious illnesses.

7.8.2 Infusion-site Reaction Assessments

Subjects should be carefully observed during IMP infusions for infusion-site reactions as per the Schedule of Assessments. If an infusion-site reaction is evident at the time of discharge from the study center, the subject should be monitored daily (or more frequently) until the reaction is resolved.

Infusion-site reactions will be reported as AEs and will be graded for severity in accordance with the CTCAE Version 5.0.

7.8.3 Vital Signs

Height (only at Screening) and weight will be measured and recorded at the time points specified in the Schedule of Assessments.

Vital signs (temperature [sublingual], pulse rate, respiratory rate, and blood pressure [systolic and diastolic]) will be measured at Screening after the subject has rested in the supine position for at least 5 minutes and in the standing position within 2 to 3 minutes when changing from the supine to standing position (blood pressure and pulse rate only). On Day -1, vital signs will be measured after the subject has rested in the supine position for at least 5 minutes. At all other time points outlined in the Schedule of Assessments, vital signs will be measured after the subject has rested in the seated position for at least 5 minutes.

Vital signs (to be taken before blood collection for laboratory tests) will consist of 1 pulse rate and 3 blood pressure measurements (3 consecutive blood pressure readings will be recorded at intervals of at least 1 minute). The average of the 3 blood pressure readings will be recorded on the eCRF.

The acceptable vital sign ranges for study inclusion (at Screening and pre-inoculation/pre-IMP dosing) are:

**Parameter**	**Range**
Systolic blood pressure	90 to 140 mmHg
Diastolic blood pressure	40 to 90 mmHg
Pulse rate	40 to 100 bpm

The normal ranges for vital signs for subjects on the study are:

**Parameter**	**Range**
Systolic blood pressure	90 to 140 mmHg
Diastolic blood pressure	50 to 90 mmHg
Pulse rate	50 to 100 bpm
Temperature	35.0 to 37.5°C
Respiratory rate	10 to 25 breaths/min

7.8.4 Electrocardiograms

- Triplicate 12-lead ECG will be obtained as outlined in the Schedule of Assessments after at least 5 minutes of rest in the supine position using an ECG machine that automatically calculates the heart rate and measures PR, QRS, QT, and QTc intervals.

The QTc interval will be calculated using the prolonged Fridericia's QTcF and the prolonged Bazett's QTcB.

- At each time point, 3 individual ECG tracings should be obtained as close as possible in succession, but no more than 1 minute apart. The full set of triplicates should be completed in less than 4 minutes. ECG tracings will be retained and labeled as per standard procedures at the clinical unit, and the triplicate values will be recorded in the eCRF.
- Any clinically significant findings will be discussed with the Medical Monitor and Sponsor and documented as AEs. The Investigator will sign and date each ECG as evidence of their review.

7.8.5 Clinical Laboratory Assessments

The clinical laboratory tests to be performed and to the Schedule of Assessments for the timing and frequency. Blood and urine samples for clinical laboratory assessments will be collected according to study site SOPs.

Blood will be collected for clinical laboratory evaluations, including hematology, biochemistry, coagulation, serology. Subjects should have fasted for at least 8 hours prior to the safety laboratory tests at Screening. Blood samples will be collected either by direct venipuncture (any suitable vein) or via an indwelling cannula inserted in a forearm vein (depending on the time point). The blood volume required for these tests will be outlined in the Laboratory Manual. The total volume of blood drawn from each subject will not exceed 450 mL in any given 30-day period. This volume includes an allowance for unscheduled safety laboratory assessments that may be required at the discretion of the Investigator or the Sponsor to ensure subject safety.

Urine will be collected for urinalysis and urine drug screening. A commercially available breathalyzer test will be used to determine the concentration of alcohol in the subject's breath according to the Schedule of Assessments.

Safety Laboratory Assessments

Additional reflex testing may be conducted by the local laboratory (as per their SOPs) if safety laboratory values for a subject fall outside of the normal range/parameters. Unscheduled testing will be performed at the Investigator's discretion.

- The Investigator must review the laboratory report, document this review, and record any clinically relevant changes occurring during the study in the AE section of the eCRF. The laboratory reports must be filed with the source documents. Clinically significant abnormal laboratory findings are those that are not associated with the

subject's health status unless judged by the Investigator to be more severe than expected for the subject's condition.

- All laboratory tests with values considered clinically significantly abnormal during participation in the study should be repeated until the values return to normal or baseline or are no longer considered clinically significant by the Investigator or Medical Monitor, or until the subject is referred to a general practitioner or medical specialist as appropriate. The Investigator will document the clinical significance of all results falling outside of the normal reference ranges. All abnormal laboratory test results judged as being clinically significant will be recorded as AEs.
  - If such values do not return to normal/baseline within a period of time judged reasonable by the Investigator, the etiology should be identified and the Sponsor notified.
  - All protocol-required laboratory assessments must be conducted in accordance with the Laboratory Manual and the Schedule of Assessments.
  - If laboratory values from non-protocol specified laboratory assessments performed at the institution's local laboratory require a change in subject management or are considered clinically significant by the Investigator (e.g., SAE or AE or dose modification), then the results must be recorded in the eCRF.

7.9 Adverse Events

The definitions of an AE or SAE can be found in Appendix 3.

Adverse events can be spontaneously reported by the subject, observed by the Investigator (either directly or by laboratory or other assessments), or elicited by general questioning.

The Investigator and any designees are responsible for detecting, documenting, and recording events that meet the definition of an AE, SAE, or Suspected Unexpected Serious Adverse Reaction (SUSAR), and remain responsible for following up AEs that are serious, considered related to the study treatment or study procedures, or that caused the subject to discontinue the study.

7.9.1 Time Period and Frequency for Collecting AE and SAE Information

For the study, all AEs/SAEs will be recorded from the time of signing of the ICF until the EOS/ET visit (Section "Schedule of Activities"). The method of recording, evaluating, and assessing the severity and causality of AEs and SAEs and the procedures for completing and transmitting SAE reports are provided in Appendix 3.

All SAEs, whether related or unrelated and regardless of expectedness, should be recorded from the time of signing the ICF until study completion on the SAE Form in the eCRF, which will be immediately submitted to the Sponsor within 24 hours of site awareness, as indicated in Appendix 3. The Investigator will take immediate appropriate action in response to SAEs to ensure subject safety and attempt to identify the cause/s of the event.

The Investigator will also notify the Medical Monitor of any SAE within 24 hours of becoming aware of the event. The Investigator will submit any updated SAE data to the same recipients as the initial report within 24 hours of it being available.

Investigators are not obligated to actively seek AE or SAE after the conclusion of the study participation. However, if the Investigator learns of any SAE, including a death, at any time after a subject has been discharged from the study, and the Investigator considers the event to be reasonably related to the study treatment or study participation, the Investigator must promptly notify the Sponsor.

7.9.2 Method of Detecting AEs and SAEs

Care will be taken not to introduce bias when detecting AEs and/or SAEs. Open-ended, and non-leading verbal questioning of the subject is the preferred method to inquire about AE occurrences.

7.9.3 Follow-up of AEs and SAEs

After the initial AE/SAE report, the Investigator is required to follow each subject at subsequent visits/contacts proactively. All SAEs will be followed until resolution, stabilization, the event is otherwise explained, or the subject is lost to follow-up. Further information on follow-up procedures is given in Appendix 3.

7.9.4 Regulatory Reporting Requirements for SAEs

Prompt notification by the Investigator to the Sponsor of an SAE is essential so that legal obligations and ethical responsibilities toward the safety of subjects and the safety of a study treatment under clinical investigation are met.

The Sponsor has a legal responsibility to notify both the local regulatory authority and other regulatory agencies about the safety of a study treatment under clinical investigation. The Sponsor will comply with country-specific regulatory requirements relating to safety reporting to the regulatory authority and Investigators.

Investigator safety reports must be prepared for SUSARs according to local regulatory requirements and Sponsor policy and forwarded to Investigators as necessary.

An Investigator who receives an Investigator's safety report describing an SAE or other specific safety information (e.g., summary or listing of SAEs) from the Sponsor will

review and then file it along with the IB and will notify the HREC, if appropriate according to local requirements.

7.10 Pregnancy

- Details of all pregnancies in female subjects and female partners of male subjects will be collected after the start of study treatment and until 3 months after dosing (or 5 terminal half-lives, whichever is longer).
- If pregnancy is reported, the Investigator should inform the Sponsor within 24 hours of learning of the pregnancy.
- Abnormal pregnancy outcomes (e.g., spontaneous abortion, fetal death, stillbirth, congenital anomalies, ectopic pregnancy) are considered SAEs.

7.11 Pharmacokinetics

Collection of Samples

Venous blood samples will be collected for measurement of serum concentrations of meplazumab at time points specified in the Schedule of Assessments. Additional samples may be collected during the study is warranted and agreed upon between the Investigator and the Sponsor. Instructions for the collection and handling of biological samples will be described in a separate Laboratory Manual. Blood samples will be taken either by direct venipuncture (any suitable vein) or an indwelling cannula inserted in a forearm vein. The actual date and time (24-hour clock time) of each sample will be recorded in the eCRF. Any sampling problems will be documented in the eCRF. The time and date of study treatment administration will also be recorded. Samples will be collected, labeled, stored, and shipped as detailed in the Laboratory Manual.

PK sample collection windows are specified in the Schedule of Assessments (Table 1 and Table 2).

Determination of Drug Concentration

- Samples for the determination of meplazumab in serum will be analyzed using appropriate validated bioanalytical methods. Full details of the bioanalytical methods will be described in a separate Bioanalytical Report.
- All samples still within the known stability of the analyte of interest at the time of receipt by the bioanalytical laboratory will be analyzed.
- Samples collected from subjects who received placebo in study will be analyzed.

- The remaining serum samples may be subjected to further analysis by the Sponsor or designee for the purpose of the development of additional bioanalytical assays. Samples collected for analyses of meplazumab serum concentration may also be used to evaluate safety or efficacy aspects related to concerns arising during or after the study.

Drug concentration information that may unblind the study will not be reported to study centers or blinded personnel until the study has been unblinded. Interim PK analyses will be performed blinded (i.e., utilizing dummy subject numbers that cannot be linked to the subject identifiers used at the study site).

Any changes in the timing or addition of time points for any planned study assessments must be documented and approved by the relevant study team member and then archived in the Sponsor and study center study files but will not constitute a protocol amendment. The HREC will be informed of any safety issues that require alteration of the safety monitoring scheme or amendment of the ICF.

7.12 Receptor Occupancy Assay

Venous blood samples will be collected for measurement of receptor occupancy (RO%) assay variables at time points specified in the Schedule of Assessments.

RO% assays using flow cytometry will be designed to identify, quantitate, and monitor the binding of meplazumab to its target expressing on the erythrocytes after treatment. The RO% assay will be a semiquantitative assay that can be used to evaluate receptor binding, which could be further used for modeling PK/PD relationships.

Peripheral venous blood will be collected from subjects at each sampling point. Blood cells will be saturated in vitro with unlabeled meplazumab or with a human IgG2 isotype control antibody at equivalent concentrations. Cells will be co-stained with fluorophore-labeled anti-human IgG to detect meplazumab bound to cell-surface CD147 molecules and followed by flow cytometric analysis. Therefore, RO% will be estimated as the ratio of differences in mean fluorescent intensity of the cells pre-saturated with hIgG4 control (indicating in vivo binding of meplazumab to CD147) versus cells saturated in vitro with meplazumab (indicating all available CD147 binding sites).

8 STATISTICAL CONSIDERATIONS

8.1 Statistical Hypotheses

Given that this is a Phase I FIH study, no formal statistical hypothesis testing will be performed.

8.2 Sample Size Determination

Given the exploratory nature of this study, the sample size in the phase I clinical trial is not based on formal statistical calculations but is considered adequate to characterize the distribution of the planned endpoints.

The sample size was estimated based on a minimum number of subjects necessary to obtain a preliminary clinical assessment regarding the drug's safety profile and PK over the planned dosage while exposing as few subjects as possible to the investigational product and procedures. A sample size of 5 cohorts with 4-7 subjects per cohort is expected to be sufficient to meet the objectives of this study.

8.3 Populations

For purposes of analysis, the analysis sets in Table 7 are defined.

Table 7 Analysis Sets

Analysis Set	Description
Entered Analysis Set	All subjects who sign the ICF.
Enrolled Analysis Set	All subjects who have met all eligibility criteria. This set will be used for subject disposition.
Safety Analysis Set	All enrolled subjects who have received a single dose of meplazumab or placebo and have undergone at least one safety assessment after baseline.
Pharmacokinetic Analysis Set	All subjects who have received the study drug and have at least one calculable pharmacokinetic parameter result, and no major protocol violations that affect pharmacokinetics have occurred.
RO% Analysis Set	All subjects who have received a single dose of meplazumab or placebo and have at least 1 RO% result collected postdose without important protocol deviations/violations or events thought to significantly affect the RO%.

ICF = informed consent form; PK = pharmacokinetics; RO = receptor occupancy.

8.4 Statistical Analyses

The following sections describe the statistical analysis as it is foreseen when the study is being planned. A detailed Statistical Analysis Plan (SAP) will be developed and finalized before database lock and will describe the subject analysis sets to be included in the analyses, and procedures for accounting for missing, unused, and spurious data. The SAP will also provide the format of listings and tables to be provided for completion of the Clinical Study Report (CSR). Any deviations from the SAP will be described and justified in the final CSR. This section is a summary of the planned statistical analyses of the primary, secondary, and exploratory endpoints.

All statistical analyses, summaries, and listings will be performed using SAS® software (Version 9.4 or higher).

The following descriptive statistics will be used as applicable to summarize the study data unless otherwise specified:

- Continuous variables: sample size [n], mean, standard deviation [SD], median, minimum [min], and maximum [max].
- Categorical variables: frequencies and percentages.

The baseline will be defined as the last variable, valid, non-missing assessment (scheduled or unscheduled) prior to IMP dosing.

Individual subject data will be presented in listings.

8.5 Study Population Data

Disposition including the total number of subjects randomized and treated in the study; the total number of subjects who complete the study; and the number of subjects that prematurely discontinue from the study, along with the reason for premature discontinuation, will be summarized by treatment group and listed for all enrolled subjects. The number and percentage (%) of subjects in each analysis set will also be presented.

Subject demographic data will be summarized by descriptive statistics.

Medical history, current medical conditions, prior and concomitant medications, results of laboratory screening tests, drug and alcohol screening tests, and any other relevant baseline information will be listed by subject and cohort.

The medical history will be listed by subject and coded using the latest version of the Medical Dictionary for Regulatory Activities (MedDRA). Prior and concomitant

medications per the World Health Organization-Drug dictionary Reference List (WHO-DD) will also be recorded on the eCRF.

8.6 Efficacy Analyses

Not applicable.

8.7 Pharmacokinetic and Pharmacodynamic Analyses

8.7.1 Pharmacokinetic Analysis

Pharmacokinetic parameters will be derived using standard noncompartmental methods with Phoenix® WinNonlin® Version 6.4 or higher (Certata, L.P. Princeton, New Jersey, US) and/or SAS Version 9.4 or higher (SAS Institute, Inc., Cary, North Carolina, US). Actual elapsed time from dosing will be used for the final serum PK parameter calculations. Interim PK analyses will be performed by using scheduled time postdose for all calculations.

Serum Pharmacokinetic Parameters

The PK parameters in Table 8 will be determined for serum meplazumab when possible.

Table 8 Pharmacokinetic Parameters

**Pharmacokinetic Parameter**	**Definition**
C_{max}	Maximum concentration, obtained directly from the observed concentration-versus-time data.
T_{max}	Time to C_{max}.
C_{min}	Minimum concentration, obtained directly from the observed concentration-versus-time data.
$AUC_{(0\text{-}inf)}$	Area under the serum concentration-time curve from time zero extrapolated to infinity, calculated by linear up/log down trapezoidal summation.
$AUC_{(0\text{-}last)}$	Area under the serum concentration-time curve from time zero to the time of the last quantifiable concentration, calculated by linear up/log down trapezoidal summation.
CL	Systemic clearance
V_z	Volume of distribution
$V_{z,ss}$	Volume of distribution at steady state
$t_{½}$	Terminal half-life; a minimum of 3 points will be used for estimation.

Additional serum PK parameters may be calculated if deemed appropriate.

meplazumab serum concentrations and calculated PK parameters will be listed and descriptively summarized. Results will be presented by treatment dose level, study day, and scheduled time point, as applicable.

Arithmetic mean (±SD) and individual concentration-versus-time curves will be plotted on a linear and semilogarithmic scale, as appropriate.

8.7.2 Receptor occupancy rate Analysis

Receptor occupancy rate Parameters

The serum PD parameters in Table 9 will be determined when possible.

Table 9 Serum Pharmacodynamic Parameters

**Pharmacodynamic Parameter**	**Description**
RO	Receptor occupancy rate, expressed as a percent, is calculated by dividing background-corrected mean fluorescent intensity (MFI) in the collected blood sample by the background-corrected MFI in a meplazumab-saturated blood sample

8.8 Safety Analyses

All safety analyses will be performed on the Safety Analysis Set.

Safety data will be summarized using applicable descriptive statistics and listed separately for each cohort of the study. All placebo subjects will be combined into a treatment group (Placebo). All AEs occurring over the study period (including pre-IMP or pre-inoculation AEs) and treatment-emergent AEs (TEAEs) will be summarized by treatment (dose cohort), severity, and relationship as well as for all meplazumab doses combined versus placebo.

Adverse events will be coded using the Medical Dictionary for Regulatory Activities. The overall number and percentage of subjects with at least one AE (and SAE) will be tabulated over the entire study period. All AE data will be summarized by treatment received and study period. For each study treatment and study period, the frequency of TEAEs will be tabulated by preferred term and system organ class. Treatment-emergent AEs by maximum severity, TEAEs by relationship to study treatment, SAEs, TEAEs leading to death, and TEAEs leading to discontinuation of the study will be tabulated for each treatment group.

Summary statistics, including the change from baseline, for vital signs, laboratory parameters, ECG intervals, and infusion-site reactions, will be provided by treatment, dose, visit, and time point, as appropriate.

8.9 Missing Data

Data from subjects who withdraw from the study, including AEs and any follow-up, will be included in the analyses of primary, secondary, and exploratory outcomes.
Missing data (including those due to early discontinuations) will be not be imputed.

8.10 Interim Analyses

No formal interim analysis will be planned in this study.
Furthermore, there will be safety, PK, and PD data reviews planned in the study after subjects in each dose cohort complete the study up to 1 week postdose. The data reviews will be conducted to decide whether the dose level for the next cohort can be escalated.

9 REFERENCES

1. Knipe DM, Howley PM. Fields Virology. sixth edition ed. Two Commerce Square, 2001 Market Street, Philadelphia, PA 19103 USA: LIPPINCOTT WILLIAMS & WILKINS; 2013.

2. Peiris JS, Chu CM, Cheng VC, et al. Clinical progression and viral load in a community outbreak of coronavirus-associated SARS pneumonia: a prospective study. *Lancet* 2003; **361**(9371): 1767–72.

3. Memish ZA, Perlman S, Van Kerkhove MD, Zumla A. Middle East respiratory syndrome. *Lancet* 2020.

4. Huang C, Wang Y, Li X, et al. Clinical features of patients infected with 2019 novel coronavirus in Wuhan, China. *Lancet* 2020.

5. Lu R, Zhao X, Li J, et al. Genomic characterization and epidemiology of 2019 novel coronavirus: implications for virus origins and receptor binding. *Lancet* 2020.

6. Zhu N, Zhang D, Wang W, et al. A Novel Coronavirus from Patients with Pneumonia in China, 2019. *N Engl J Med* 2020.

7. Xu Z, Shi L, Wang Y, et al. Pathological findings of COVID-19 associated with acute respiratory distress syndrome. *Lancet Respir Med* 2020.

8. Novel Coronavirus Pneumonia Emergency Response Epidemiology T. [The epidemiological characteristics of an outbreak of 2019 novel coronavirus diseases (COVID-19) in China]. *Zhonghua Liu Xing Bing Xue Za Zhi* 2020; **41**(2): 145–51.

9. https://www.who.int/emergencies/diseases/novel-coronavirus-2019

10 APPENDICES

Appendix 1 Abbreviations

Abbreviation	Definition
ADA	Anti-drug antibody
AE	Adverse event
ALT	Alanine aminotransferase
AST	Aspartate aminotransferase
$AUC_{(0\text{-inf})}$	Area under the concentration-time curve from time zero extrapolated to infinity, calculated by linear up/log down trapezoidal summation
$AUC_{(0\text{-last})}$	Area under the concentration-time curve from time zero to the time of the last quantifiable concentration, calculated by linear up/log down trapezoidal summation
BMI	Body mass index
CAPA	Corrective and preventative action
C_{max}	Maximum concentration, obtained directly from the observed concentration-versus-time data
CMI	Consumer medicine information
CRO	Contract research organization
CSR	Clinical study report
CTCAE	Common Terminology Criteria for Adverse Events
DBIL	Direct bilirubin
DNA	Deoxyribonucleic acid
EC_{50}	50% effective concentration
EC_{90}	90% effective concentration
ECG	Electrocardiogram
eCRF	Electronic case report form
ELISA	Enzyme-linked immunosorbent assay
EOS	End of study
ET	Early termination
FDA	Food and Drug Administration
FIH	First-in-human
GLP	Good Laboratory Practice
HREC	Human research ethics committee

Abbreviation	Definition
HRT	Hormone replacement therapy
IB	Investigator’s brochure
ICF	Informed consent form
ICH	International Council for Harmonisation
IgG2	Immunoglobulin G2
IMP	Investigational medicinal product
INR	International normalized ratio
LDH	Lactate dehydrogenase
LFT	Liver function test
MCB	Master cell bank
MedDRA	Medical Dictionary for Regulatory Activities
MIC	Minimum inhibitory concentration
MPC	Minimum parasiticidal concentration
NOAEL	No observed adverse effect level
NTF	Note to file
PCR	Polymerase chain reaction
PD	Pharmacodynamic(s)
PK	Pharmacokinetic(s)
QTcB	Bazett’s QT correction formula
QTcF	Fridericia’s QT correction formula
RO%	Receptor occupancy rate
SAE	Serious adverse event
SAP	Statistical analysis plan
SOP	Standard operating procedures
SUSAR	Suspected unexpected serious adverse reaction
$t_{½}$	Terminal half-life
TBIL	Total bilirubin
TEAE	Treatment-emergent adverse event
WOCBP	Woman of childbearing potential

Appendix 2 Regulatory, Ethical and Study Oversight Considerations

Regulatory and Ethical Considerations

- This study will be conducted in accordance with the protocol and with the following:
    - Consensus ethical principles derived from international guidelines including the Declaration of Helsinki and Council for International Organizations of Medical Sciences International Ethical Guidelines.
    - Applicable International Council on Harmonisation (ICH) Good Clinical Practice (GCP) Guidelines.
    - Therapeutic Goods Administration (2000) (NHMRC National Statement on Ethical Conduct in Human Research – 2007, updated 2018).
    - Applicable laws and regulations.
- The protocol, protocol amendments, Informed Consent Form (ICF), Investigator Brochure, and other relevant documents (e.g., advertisements) must be submitted to the Human Research Ethics Committee (HREC) by the Investigator and reviewed and approved by the HREC before the study is initiated.
- Any amendments to the protocol will require HREC and regulatory authority approval, when applicable, before implementation of changes made to the study design, except for changes necessary to eliminate an immediate hazard to subjects.
- The Investigator will be responsible for the following:
    - Providing written summaries of the status of the study to the HREC annually or more frequently in accordance with the requirements, policies, and procedures established by the HREC.
    - Notifying the HREC of serious adverse events (SAEs) or other significant safety findings as required by HREC procedures.
    - Providing oversight of the conduct of the study at the study center and adherence to requirements of the Therapeutics Goods Administration, ICH guidelines, the HREC, and all other applicable local regulations.
- After reading the protocol, each Investigator will sign the protocol signature page and send a copy of the signed page to the Sponsor or representative. The study will not start at any study center at which the Investigator has not signed the protocol.

Adequate Resources

The Investigator is responsible for supervising any individual or party to whom the Investigator delegates study-related duties and functions conducted at the study center.

If the Investigator/institution retains the services of any individual or party to perform study-related duties and functions, the Investigator/institution should ensure this individual or party is qualified to perform those study-related duties and functions and should implement procedures to ensure the integrity of the study-related duties and functions performed and any data generated.

Financial Disclosure

Investigators and sub-Investigators will provide the Sponsor with sufficient, accurate financial information as requested to allow the Sponsor to submit a complete and accurate financial certification or disclosure statements to the appropriate Regulatory Authorities. Investigators are responsible for providing information on financial interests during the course of the study and for 1 year after completion of the study.

Insurance

The Sponsor will ensure sufficient insurance is available to enable it to indemnify and hold the Investigator(s) and relevant staff as well as any hospital, institution, ethics committee, or the like, harmless from any claims for damages for unexpected injuries, including death, that may be caused by the subject's participation in the study but only to the extent that the claim is not caused by the fault or negligence of the subject(s) or Investigator(s). The Sponsor adheres to the guidelines of Medicines Australia for injury resulting from participation in a company-sponsored study, including the provision of 'No-fault clinical study insurance'.

Informed Consent Process

- The Participant Information Sheet and ICF describe in detail the study interventions, procedures, and risks.
- The Investigator or their representative will explain the nature of the study to the subject and answer all questions regarding the study.
- Subjects must be informed that their participation is voluntary. Subjects will be required to sign a statement of informed consent that meets the requirements of the Therapeutic Goods Administration, ICH guidelines, all other applicable local laws, and the HREC or study center.
- The medical record must include a statement that written informed consent was obtained before the subject was entered in the study and the date the written consent was obtained. The authorized person obtaining the informed consent must also sign the ICF.

- Subjects must be re-consented to the most current version of the ICF(s) during their participation in the study.
- A copy of the ICF(s) must be provided to the subject or the subject's legally authorized representative.
- For Parts B and C, subjects will also receive an Informed Consent for Blood Storage and an option to grant permission to be contacted about future study involvement.
- A subject who is rescreened is not required to sign another ICF if the rescreening occurs within 28 days from the previous ICF signature date.

The ICF will contain a separate section that addresses the use of remaining mandatory samples for optional exploratory research. The Investigator or authorized designee will explain to each subject the objectives of the exploratory research. Subjects will be told that they are free to refuse to participate and may withdraw their consent at any time and for any reason during the storage period. A separate signature will be required to document a subject's agreement to allow any remaining specimens to be used for exploratory research. Subjects who decline to participate in this optional research will not provide this separate signature.

Data Protection

- Subjects will be assigned a unique identifier by the Sponsor. Any subject records or datasets that are transferred to the Sponsor will contain the identifier only; subject names or any information which would make the subject identifiable will not be transferred.
- The subject must be informed that their personal study-related data will be used by the Sponsor in accordance with local data protection law. Subjects will be informed that their data will be held on file by the clinical study site and that the data may be viewed by the staff of the clinical study site (including, where necessary, clinical study site staff other than the Investigator). The level of disclosure must also be explained to the subject.
- Upon request, the Investigator/institution(s) will permit direct access to source data and documents for study-related monitoring, audits, ethics committee review, and regulatory inspection(s) by the Sponsor (or their appropriately qualified delegates) and Regulatory Authorities. The subject must be informed that their medical records may be examined by Clinical Quality Assurance auditors or other authorized personnel appointed by the Sponsor, by appropriate HREC members, and by inspectors from Regulatory Authorities.

- Subjects will also be informed that a report of the study will be submitted to the Sponsor and may also be submitted to government agencies and perhaps for publication, but that they will only be identified in such reports by their study identification number, initials and perhaps their gender and age. The Investigators undertake to hold all personal information in confidence.
- Subjects will be informed that samples collected for the purposes described in the protocol will be sent to the Sponsor's nominated national or international laboratory for assessment.

Dissemination of Clinical Study Data

After completion of the study, a Clinical Study Report (CSR) will be written by the Sponsors/designees in consultation with the Investigator following the guidance in ICH E3.

Data Quality Assurance

- All subject data relating to the study will be recorded on printed or electronic case report forms (eCRFs) unless transmitted to the Sponsor or designee electronically (e.g., laboratory data). The Investigator is responsible for verifying that data entries are accurate and correct by physically or electronically signing the eCRF.
- The Investigator must maintain accurate documentation (source data) that supports the information entered in the eCRF.
- The Investigator must permit study-related monitoring, audits, HREC review, and regulatory agency inspections and provide direct access to source data documents. The Sponsor will require full verification for the presence of informed consent, adherence to the eligibility criteria, documentation of SAEs, and the recording of data that was used for all primary and safety variables. Additional checks of the consistency of the source data with the eCRFs will be performed according to the study-specific monitoring plan. No information in source documents about the identity of the subjects will be disclosed.
- The CRO is responsible for the data management of this study including quality checking of the data.
- Study monitors will perform ongoing source data verification to confirm that data entered into the eCRF by authorized study center personnel are accurate, complete, and verifiable from source documents; that the safety and rights of subjects are being protected; and that the study is being conducted in accordance with the currently approved protocol and any other study agreements, ICH GCP, and all applicable

regulatory requirements. Key study personnel is required to be available to assist the field monitor during these visits.

- The study will be monitored according to the SOPs of the monitoring CRO appointed this task by the Sponsor, and all protocol deviations will be reported to the Sponsor. Serious breaches that impact subject safety or data integrity will also be reported to the Therapeutic Goods Administration and QIMR Berghofer HREC.
- Records and documents, including signed ICFs, pertaining to the conduct of this study must be retained by the Investigator for 15 years after study completion unless local regulations or institutional policies require a longer retention period. No records may be destroyed during the retention period without the written approval of the Sponsor. No records may be transferred to another location or party without written notification to the Sponsor.

Source Documents

The Investigator/institution should maintain adequate and accurate source documents and study records that include all pertinent observations on each of the study center's subjects. Source data should be attributable, legible, contemporaneous, original, accurate, and complete. Changes to source data should be traceable, should not obscure the original entry, and should be explained if necessary (e.g., via an audit trail).

- Source documents provide evidence for the existence of the subject and substantiate the integrity of the data collected. Source documents are filed at the Investigator's study center.
- Data reported entered in the eCRF that are transcribed from source documents must be consistent with the source documents, or the discrepancies must be explained. The Investigator may need to request previous medical records or transfer records, depending on the study. Also, current medical records must be available.

Protocol Deviations

Protocol Deviation: a protocol deviation is any departure, change, and/or addition from the study design or procedures defined in the approved protocol.

Suspected serious breach: a report that is judged by the reporter as a possible serious breach of GCP but has yet to be formally confirmed as a serious breach by the Sponsor.

Serious breach: A breach of GCP or the protocol that is likely to affect to a significant degree: a) the safety or rights of a study subject, or b) the reliability and robustness of the data generated in the clinical study.

Note to File (NTF): a note to file is a record that documents in detail actions taken, important decisions made, or explains a sequence of events where no other detailed record exists to enable the conduct of the study to be reconstructed. Note – an NTF is an unacceptable way to recode deviations during the study.

Corrective and Preventative Action Plans (CAPA): a CAPA plan will be developed if requested by the Sponsor, HREC, or other authorized parties as outlined in the Sponsor's SOP.

Reporting requirements:

- All protocol deviations will be documented in the source documents and included in the CSR.
- All NTFs, protocol deviations, suspected serious breaches and serious breaches are to be viewed by the Investigator's delegate and signed by the Investigator.
- All NTFs, protocol deviations, suspected serious breaches and serious breaches will be assessed and assigned significance at the end of each cohort by the study team.
- All suspected serious breaches are to be reported by the clinical study site to the Sponsor within 72 hours. If the Sponsor identifies a serious breach, assessment should be made and include:
  - If the incident is isolated or persistent.
  - Impact on safety and data.
  - Assessment of cause.
  - Assessment on reporting requirements.
  - If a CAPA plan is required.
- Serious Breaches should be reported by the Sponsor to the clinical study site and HREC within 7 days as well as the TGA and other relevant Regulatory Authorities if the breach has led to a clinical study site closure or involve a defective product that has a wider supply implication.
- All protocol deviations will be reported by the clinical study site to the Investigator as early as possible but within 7 days and to the Sponsor at the end of each cohort.
- Protocol deviation logs will be submitted by the clinical study site to the Sponsor and QIMR Berghofer HREC via inclusion with the annual report.

Study and Study Center Closure

The Sponsor, Principal Investigator, FMD, and Regulatory Authorities independently reserve the right to discontinue the study at any time for safety or other reasons. This will be done in consultation with the Sponsor where practical.

Study centers will be closed upon study completion. A study center is considered closed when all required documents and study supplies have been collected, and a study center closure visit has been performed.

The Investigator may initiate study center closure at any time, provided there is reasonable cause, and sufficient notice is given in advance of the intended termination.

Reasons for the early closure of a study center by the Sponsor or Investigator may include but are not limited to:

- Failure of the Investigator to comply with the protocol, the requirements of the HREC or local health authorities, the Sponsor’s procedures, or GCP guidelines.
- Inadequate recruitment of subjects by the Investigator.
- Discontinuation of further study treatment development.

In the event of premature study termination or suspension, the above-mentioned parties will be notified in writing by the terminator/suspender stating the reasons for early termination or suspension (with the exception of the Sponsor’s responsibility for notifying the Regulatory Authorities). After such a decision, the Sponsor and the Investigator will ensure that adequate consideration is given to the protection of the subjects’ interest and safety. The Investigator must review all subjects as soon as practical and complete all required records.

Publication Policy

The data management, statistical, and medical writing team appointed by the Sponsor will collaborate to provide a detailed CSR upon the conclusion of the study. This will include appendices of all tables and listings generated during the analyses of data. The tables, figures, and listings will be provided by the Sponsor. The Sponsor undertakes to ensure that all safety observations made during the conduct of the study are documented in this report.

Publication and reporting of results and outcomes of this study will be accurate and honest and undertaken with integrity and transparency. The Sponsor recognizes that the Principal Investigator has a responsibility to ensure that results of scientific interest arising from the study are appropriately published and disseminated. Publication of results will be subjected to fair peer-review. Authorship will be given to all persons providing significant input into the conception, design, and execution or reporting of the research. No person who is an author, consistent with this definition, will be excluded as an author without their permission in writing. Authorship will be discussed between researchers prior to study commencement (or as soon as possible thereafter) and

reviewed whenever there are changes in participation. The acknowledgment will be given to collaborating institutions and hospitals, and other individuals and organizations providing finance or facilities.

In any press releases, publications or presentations, the financial contribution from the Sponsor to the study and its participation in the collaboration shall be expressly acknowledged. Data will not be released publicly until the manuscript is accepted for publication. In the case of no publication, the information will only be released to the public and media in accordance with the sponsor. However, the Investigator undertakes not to make any publication or release pertaining to the study and/or results of the study without the Sponsor's prior written consent, being understood that the Sponsor will not unreasonably withhold its approval. The Sponsor has the right to publish the results of the study at any time.

The Investigator shall not use the name of the Sponsor and/or of its employees in advertising or promotional material or publication without the prior written consent of the Sponsor. The Sponsor shall not use the name of the Investigator and/or the collaborators in advertising or promotional material or publication without having received their prior written consent(s).

The Sponsor will ensure that the key design elements of this protocol are posted in a publicly accessible database such as Clinical Trials Registry or Clinicaltrials.gov. In addition, upon study completion and finalization of the study report, the results of this study will be either submitted for publication in an open-access journal and/or posted in a publicly accessible database of clinical study results.

Appendix 3 Adverse Events: Definitions and Procedures for Recording, Evaluating, Follow-up, and Reporting

Definition of Adverse Event

AE Definition
• An adverse event (AE) is any untoward medical occurrence in a subject, temporally associated with the use of study treatment, whether or not considered related to the study treatment.<br>• NOTE: An AE can therefore be any unfavorable and unintended sign (including an abnormal laboratory finding), symptom, or disease (new or exacerbated) temporally associated with the use of study treatment.

Events <u>Meeting</u> the AE Definition
• Any abnormal laboratory test results (hematology, biochemistry, or urinalysis) or other safety assessments (e.g., electrocardiogram, radiological scans, vital signs measurements), including those that worsen from baseline, considered clinically significant in the medical and scientific judgment of the Investigator (i.e., not related to the progression of underlying disease).<br>• Exacerbation of a chronic or intermittent pre-existing condition, including either an increase in frequency and/or intensity of the condition.<br>• New conditions detected or diagnosed after study treatment administration even though it may have been present before the start of the study.<br>• Signs, symptoms, or the clinical sequelae of a suspected drug-drug interaction.<br>• Signs, symptoms, or the clinical sequelae of a suspected overdose of either study treatment or a concomitant medication. Overdose per se will not be reported as an AE/serious adverse event (SAE) unless it is an intentional overdose taken with possible suicidal/self-harming intent. Such overdoses should be reported regardless of sequelae.

Events NOT Meeting the AE Definition
• Medical or surgical procedure (e.g., endoscopy, appendectomy): the condition that leads to the procedure is the AE.<br>• Situations in which an untoward medical occurrence did not occur (social and/or convenience admission to a hospital).<br>• Anticipated day-to-day fluctuations of pre-existing disease(s) or condition(s) present or detected at the start of the study that does not worsen.

Definition of Treatment-Emergent Adverse Event

A treatment-emergent AE is an event that commences or a pre-existing event that worsens in severity, any time on or after initiation of investigational medicinal product (IMP) administration.

Definition of Serious Adverse Event

If an event is not an AE per the definition above, then it cannot be an SAE even if serious conditions are met (e.g., hospitalization for signs/symptoms of the disease under study, death due to progression of disease).

An SAE is defined as any untoward medical occurrence that, at any dose:
**a) Results in death**
**b) Is life-threatening**<br>The term 'life-threatening' in the definition of "serious" refers to an event in which the subject was at risk of death at the time of the event. It does not refer to an event, which hypothetically might have caused death if it were more severe.
**c) Requires inpatient hospitalization or prolongation of existing hospitalization**<br>In general, hospitalization signifies that the subject has been detained (usually involving at least an overnight stay) at the hospital or emergency ward for observation and/or treatment that would not have been appropriate in the physician's office or outpatient setting. Complications that occur during hospitalization are AEs. If a complication prolongs hospitalization or fulfills any other serious criteria, the event is serious. When in doubt as to whether "hospitalization" occurred or was necessary, the AE should be considered serious.<br>Hospitalization for elective treatment of a pre-existing condition that did not worsen from baseline is not considered an AE.
**d) Results in persistent disability/incapacity**<br>• The term disability means a substantial disruption of a person's ability to conduct normal life functions.

•	This definition is not intended to include experiences of relatively minor medical significance such as uncomplicated headache, nausea, vomiting, diarrhea, influenza, and accidental trauma (e.g., sprained ankle), which may interfere with or prevent everyday life functions but do not constitute a substantial disruption.
**e)**	**Is a congenital anomaly/birth defect**
**f)**	**Other situations:**
•	Medical or scientific judgment should be exercised in deciding whether SAE reporting is appropriate in other situations such as important medical events that may not be immediately life-threatening or result in death or hospitalization but may jeopardize the subject or may require medical or surgical intervention to prevent 1 of the other outcomes listed in the above definition. These events should usually be considered serious.<br>Examples of such events include invasive or malignant cancers, intensive treatment in an emergency room or at home for allergic bronchospasm, blood dyscrasias or convulsions that do not result in hospitalization, or the development of drug dependency or drug abuse.

Expectedness of an AE or SAE

**Unexpected**
• An AE is regarded as an unexpected event if its nature or severity is not consistent with the applicable reference safety information (Investigator Brochure's [IBs] or approved manufacturer's prescribing information for marketed drugs).<br>• Events that add significant information on the specificity, severity, or frequency of previously described reactions are also regarded as unexpected.<br>• A suspected unexpected serious adverse reaction is any SAE where a causal relationship with a study intervention is at least a reasonable possibility, and the event is not listed in the IBs or product information.
**Expected**
• Expected AEs from meplazumab dosing are listed in and the meplazumab IB.

Recording and Follow-up of AE and/or SAE

**AE and SAE Recording**
• When an AE/SAE occurs, it is the responsibility of the Investigator to review all documentation (e.g., hospital progress notes, laboratory reports, and diagnostics reports) related to the event.<br>• The Investigator will then record all relevant AE/SAE information in the electronic case report form (eCRF). Each event must be recorded separately with the following information:<br>o Description of the event.<br>o Dates and times of onset and resolution.

- Duration in hours.
- Seriousness (SAE or not).
- Severity.
  - Only one AE will be reported if an AE changes in severity over time (the maximum severity will be recorded in the eCRF).
  - Changes in the severity of an AE will be documented in the source documents to allow assessment of the duration of the event at each level of severity.
  - AEs characterized as intermittent require documentation of onset and duration at each episode.
  - If the AE resolves and then reoccurs at a later date, then 2 AEs are reported.
- Action was taken in response to the AE (including treatment required)
- Outcome (recovered/resolved, recovered/resolved with sequelae, not recovered/nor resolved, fatal, unknown).

- It is **not** acceptable for the Investigator to send photocopies of the subject's medical records in lieu of completion of the AE/SAE eCRF page.
- There may be instances when copies of medical records for certain cases are requested by the Sponsor. In this case, all subject identifiers, with the exception of the subject number, will be redacted on the copies of the medical records before submission to the Sponsor.
- The Investigator will attempt to establish a diagnosis of the event based on signs, symptoms, and/or other clinical information. Whenever possible, the diagnosis (not the individual signs/symptoms) will be documented as the AE/SAE.

Severity of Event

In addition to determining whether an AE fulfills the criteria for an SAE or not, the severity of AEs experienced by subjects will be recorded in accordance with the Common Terminology Criteria for Adverse Events Version 5.0, published 27 November 2017.

The severity of AEs will be graded as follows:

- Grade 1: Mild; asymptomatic or mild symptoms; clinical or diagnostic observations only; intervention not indicated.
- Grade 2: Moderate; minimal, local or noninvasive intervention indicated; limiting age-appropriate instrumental activities of daily living.
- Grade 3: Severe or medically significant but not immediately life-threatening; hospitalization or prolongation of hospitalization indicated; disabling; limiting self-care activities of daily living.
- Grade 4: Life-threatening consequences; urgent intervention indicated.
- Grade 5: Death related to AE.

A mild, moderate, or severe AE may or may not be serious. These terms are used to describe the intensity of a specific event. Medical judgment should be used on a case-by-case basis.<br>Seriousness, rather than severity assessment, determine the regulatory reporting obligations.
**Assessment of Causality**
• The Investigator is obligated to assess the relationship between study treatment and each occurrence of each AE/SAE. The AE/SAE must be characterized as **related/suspected** and **not related/not suspected**.<br>○ "Related/Suspected": The temporal relationship between the event and the administration of the study interventions is compelling and/or follows a known or suspected response pattern to that study intervention, and the event cannot be explained by the subject's medical condition, other therapies or accident.<br>○ "Not related/Not suspected": The event can be readily explained by other factors such as the subject's underlying medical condition, concomitant therapy or accident and no plausible temporal or biologic relationship exists between any of the study interventions and the event.<br>• Alternative causes, such as underlying disease(s), concomitant therapy, and other risk factors, as well as the temporal relationship of the event to study treatment administration, will be considered and investigated.<br>• The Investigator will also consult the IB and/or Product Information, for marketed products, in their assessment.<br>• For each AE/SAE, the Investigator must document in the medical notes that they have reviewed the AE/SAE and has provided an assessment of causality.<br>• There may be situations in which an SAE has occurred, and the Investigator has minimal information to include in the initial report to the Sponsor. However, it is very important that the Investigator always make an assessment of causality for every event before the initial transmission of the SAE data to the Sponsor.<br>• The Investigator may change their opinion of causality in light of follow-up information and send a SAE follow-up report with the updated causality assessment.<br>• The causality assessment is one of the criteria used when determining regulatory reporting requirements.

**Follow-up of AEs, and SAEs**
• The Investigator is obligated to perform or arrange for the conduct of supplemental measurements and/or evaluations as medically indicated or as requested by the Sponsor to elucidate the nature

and/or causality of the AE or SAE as fully as possible. This may include additional laboratory tests or investigations, histopathological examinations, or consultation with other health care professionals.

- New or updated information will be recorded in the originally completed eCRF..

Reporting of SAEs

SAE Reporting via an Electronic Data Collection Tool
• The primary mechanism for reporting an SAE will be the electronic data collection tool.<br>• If the electronic system is unavailable for more than 24 hours, then the study center will use the paper SAE data collection tool (see next section).<br>• The study center will enter the SAE data into the electronic system as soon as it becomes available.<br>• After the study is completed at a given study center, the electronic data collection tool will be taken off-line to prevent the entry of new data or changes to existing data.<br>• If a study center receives a report of a new SAE from a subject or receives updated data on a previously reported SAE after the electronic data collection tool has been taken off-line, then the study center can report this information on a paper SAE Form (see next section) or by telephone.

SAE Reporting via Paper CRF
• Facsimile transmission of the SAE paper CRF is the preferred method to transmit this information.<br>• In rare circumstances and in the absence of facsimile equipment, notification by telephone is acceptable with a copy of the SAE data collection tool sent by overnight mail or courier service.<br>• Initial notification via telephone does not replace the need for the Investigator to complete and sign the SAE CRF pages within the designated reporting time frames.

Signature of Investigator

PROTOCOL TITLE: A Single-Center, Double-blinded, Placebo-controlled Phase I Clinical Trial in Healthy Volunteer to Evaluate Safety, Tolerance, and Pharmacokinetics of Meplazumab for Injection

PROTOCOL No.: MPZ-I-01

VERSION: V3.0

This protocol is a confidential communication of Jiangsu Pacific Meinuoke Biopharmaceutical Co. Ltd and National Translational Science Centre for Molecular Medicine of Fourth Military Medical University. I confirm that I have read this protocol, I understand it, and I will work according to this protocol. I will also work consistently with the ethical principles that have their origin in the Declaration of Helsinki and that are consistent with Good Clinical Practice and the applicable laws and regulations. Acceptance of this document constitutes my agreement that no unpublished information contained herein will be published or disclosed without prior written approval from the Sponsor.

Instructions to the Investigator: Please SIGN and DATE this signature page. PRINT your name, title, and the name of the study center in which the study will be conducted. Return the signed copy to the Sponsor and CRO.

I have read this protocol in its entirety and agree to conduct the study accordingly:

Signature of Investigator: ____________________________

Date: ____________________________

Printed Name: ____________________________

Investigator Title: ____________________________

Name/Address of Center: ____________________________
