# Supplementary material for: Safety and efficacy of meplazumab in healthy volunteers and COVID-19 patients: a randomized phase 1 and an exploratory phase 2 trial
Source: Signal Transduct Target Ther. 2021 May 17;6:194. doi: 10.1038/s41392-021-00603-6 (PMC8127508; doi:10.1038/s41392-021-00603-6)
Supplement: Supplementary file 3 — Supplementary protocol 2 [file 41392_2021_603_MOESM3_ESM.pdf]

TITLE PAGE

Protocol Title:

A Single Center Clinical Trial in Healthy Volunteer to Evaluate Biodistribution of ^{131}I-labelled Meplazumab

Protocol Number: MPZ-I-03

Amendment Number: V2.1

Product: Meplazumab for Injection (Ketantin®)

Short Title:

A Phase I Clinical Trial of ^{131}I-labelled Meplazumab in Healthy Volunteer

Study Phase: Phase I

Sponsor Name:

Jiangsu Pacific Meinuoke Biopharmaceutical Co. LTD.

Legal Registered Address:

No. 128 Hehai West Road, Xinbei District, Changzhou, 213022, Jiangsu People's Republic of China

Principal Investigator: Prof. Ping Zhu, MD, Prof. Aidong Wen, MD, Prof. Jing Wang, MD

Regulatory Agency Identifying Number:

Date of Protocol: 24 May 2020

Sponsor Signatory:

I have read this protocol in its entirety and agree to conduct the study accordingly:

**Xiaochun Chen**	**Date**
**Zhi-Nan Chen**	**Date**

.

Title Page **1**
1. Summary **5**
Schedule of Activities **7**
Table 1 Schedule of Assessments (biodistribution study) : **7**
2. Introduction **11**
2.1 Study Rationale **11**
2.2 Background **11**
2.3 Benefit/Risk Assessment **18**
2.4 Meplazumab **18**
3 Objectives and Endpoints **20**
4 Study Design **21**
4.1. Overall Design **21**
4.2. Scientific Rationale for Study Design **22**
4.3. Study Population **23**
4.4. Study Design **23**
4.5. Justification for Dose of Meplazumab **23**
4.6. End of Study Definition **24**
4.7. Study Stopping Criteria **24**
5 Study Population **26**
5.1. Inclusion Criteria **26**
5.2. Exclusion Criteria **26**
5.3. Enrollment **28**
6 Study Treatment **29**
6.1. Study Treatment(s) Administered **29**
6.2. Preparation/Handling/Storage/Accountability **30**
6.3. Measures to Minimize Bias: Randomization and Blinding **31**
6.4. Study Treatment Compliance **31**
6.5. Prior and Concomitant Therapy **32**
6.6. Dose Modification **33**
6.7. Treatment After the End of the Study **33**
7 Study Assessments and Procedures **34**
7.1 Schedule of Assessments **34**
7.2 Screening Examinations **34**
7.3 Treatment Period **35**
7.4 Outpatient Visits and Phone Calls **35**
7.5 End of Study/Early Termination Visit **35**
7.6 Demographics and Other Baseline Characteristics **35**
7.7 Medical and Surgical History **36**
7.8 Efficacy Assessments **36**
7.9 Biodistribution Assessments **36**
7.10 Pharmacokinetics **37**
7.11 Adverse Events **38**
7.12 Pregnancy **40**

8 Statistical Considerations 41
8.1 Statistical Hypotheses 41
8.2 Sample Size Determination 41
8.3 Populations 41
8.4 Statistical Analyses 41
8.5 Study Population Data 42
8.6 Biodistribution Analyses 42
8.7 Safety analyses 43
8.8 Missing Data 43
8.9 Interim Analyses 44
9 References 45
10 Appendices 46
Appendix 1 Abbreviations 46
Appendix 2 Regulatory, Ethical, and Study Oversight Considerations 49
Regulatory and Ethical Considerations 49
Adequate Resources 50
Financial Disclosure 50
Insurance 50
Informed Consent Process 50
Data Protection 51
Dissemination of Clinical Study Data 52
Data Quality Assurance 52
Source Documents 53
Protocol Deviations 54
Study and Study Center Closure 55
Publication Policy 56
Appendix 3 Adverse Events: Definitions and Procedures for Recording, Evaluating, Follow-up, and Reporting 58
Signature of Investigator 65

1. SUMMARY

Protocol Title:

A Single-Center Clinical Trial in Healthy Volunteer to Evaluate biodistribution of ^{131}I-labelled Meplazumab

Short Title:

A Clinical Trial of ^{131}I-labelled Meplazumab in Healthy Volunteer

Rationale:

This clinical trial will be conducted to evaluate the biodistribution and pharmacokinetics of ^{131}I -labeled meplazumab in healthy subjects, thus providing a new macromolecule antibody drug for the treatment of COVID-19 pneumonia.

Objectives and Endpoints:

The primary and secondary endpoints of the study are presented below.

**Objectives**	**Endpoints**
Primary	
• To evaluate the biodistribution of ^{131}I-Meplazumab in tissues	• The radiation ratio of ROI of tissues to cardiac blood-pool (T/C)
Secondary	
• To evaluate the pharmacokinetics of ^{131}I-Meplazumab	• The pharmacokinetics parameters of ^{131}I-Meplazumab in blood and urine

Overall Design:

To investigate the biodistribution of meplazumab, a single-center study was designed. In this study, 3 healthy volunteers, both male and female, will be enrolled and the dose is 5 or 10mCi ^{131}I-meplazumab by single intravenous infusion. The subjects will be conducted general condition, medical history, medication history, physical examination, vital sign measurement, drug abuse screening, blood, urine, liver and kidney function, blood coagulation function, electrocardiogram and other related tests during the screening period (-14～-2d). If the subjects met the inclusion criteria, Lugol's solution would take orally 48 hours before treatment (Day-2), 0.5mL/time, 3 times a day (meal at the same time) until 14 days post-dose. On the third day (Day0), the subjects will examine the iodine uptake rate on an empty stomach in the morning. If the iodine uptake rate results show that the ^{131}I uptake is between 8.5% and 17%, then 5mCi^{131}I-meplazumab is injected; if the iodine uptake rate shows that the ^{131}I uptake is less than 8.5%, the injection dose could be increased to 10mCi. The mode of administration is a single intravenous drip, which was finished at 60 ±5min.

Number of Investigators and Study Centers:

The study will be conducted at a single study center under the supervision of a Principal Investigator.

Number of Subjects:

Approximately 3 or more healthy men and women (18 to 50 years of age) will participate in this study.

Treatment Groups and Duration:

One cohort (n=3) is planned for biodistribution. In this cohort, subjects will receive a single dose of ^{131}I-labeled meplazumab.

Criteria for Stopping Study:

The study will be stopped if any of the stopping rules are met:

- One-third of the subjects have Grade 3 AEs in the same organ system, which are considered at least possibly related to study treatment.
- Half subjects have a Grade 2 AE in the same organ or body system, which are considered at least possibly related to study treatment.
- If the proportion of subjects who have Grade 2 (or higher) AEs in the same organ system are less than 50% or less than one-third for Grad 3, the investigator and sponsor will decide whether to stop the study.

Statistical Methods:

<u>Biodistribution analysis set</u>

The biodistribution analysis set will include all subjects who have received a single dose of ^{131}I-meplazumab and have at least 1 SPECT scanning result collected post-dose without critical protocol deviations/violations or events thought to affect the scanning significantly.

Safety Review Committee: No.

Schedule of Activities

Table 1 Schedule of Assessments (biodistribution study) :

Assessments	Screening [d]		Treatment Period																									
Day	-14~-2D	-1	0											1		2		3		4	5	6	7	8	10	14	30	90
Time relative to dose (h)			Pre	0h	15min	30min	1h	1.5h	2h	4h	8h	12h	16h	24h	36h	48h	60h	72h	84h	96h	120h	144h	168h	192h	240h	336h	720h	2160h
Visits [b]	√	√	√	√	√	√	√	√	√	√	√	√	√	√	√	√	√	√	√	√	√	√	√	√	√	√	√	√
Informed Consent	√																											
Demography	√																											
Eligibility Criteria check/recheck	√	√																										
Medical and Medication history	√																											
Smoking and drinking history, allergy history, blood donation history	√																											
Height and BMI	√																											
Physical examination	√																									√	√	
Vital sign	√	√	√				√		√	√	√	√		√		√		√		√	√	√	√	√			√	
Blood alcohol test	√																											
Urine drug abuse screening	√																											
12 lead ECG	√									√				√		√											√	√

Blood routine test	√		√																								√	√
Urine routine test	√		√																								√	√
Urine sample collection				√							√		√	√		√		√		√	√	√	√	√	√	√		
Blood biochemistry	√		√																								√	√
Thyroid function test	√																										√	√
Myocardial enzyme			√																								√	
Coagulation function	√		√																								√	
Serology (HIV, HBV, HCV), RPR (syphilis)	√																											
SARS-CoV-2 Specific antibody detection	√																											
Pregnancy Test c/FSH Test (women only)	√																										√	
Seal thyroid gland [c]	√	√	√											√		√		√		√	√	√	√	√	√	√		
Detection of the iodine absorption rate			√																									
Adm				√																								
SPECT scanning[a]							√		√		√	√		√	√	√	√	√	√	√	√	√	√	√				
Infusion-site reaction Assessment			√											√		√												
PK Sample Collection			√		√	√	√	√	√	√	√	√		√		√		√		√	√	√	√	√	√	√		
Recording of AEs/SAEs/ combination therapy		√	√	√	√	√	√	√	√	√	√	√	√	√		√		√		√	√	√	√	√	√	√		

Abbreviations: AE = adverse event; BMI = body mass index; ECG = electrocardiogram; HIV = human immunodeficiency virus; SAE = serious adverse event.

General: All post-dose time points are scheduled relative to the end of infusion (EOI). At visits where assessment time points coincide with each other, the following procedure should be followed: perform ECGs first, followed by vital signs as close as possible to the scheduled time point but prior to PK sampling; perform PK sampling at the scheduled time point; perform all other procedures as close as possible to the scheduled time point but may be obtained before or after PK sampling.

For biodistribution study, if two consecutive measuring points are not detected, the acquisition is terminated and, if there is still a heartbeat on Day 8, additional scanning per 2 or 3 days till Day 14.

For biodistribution study, the alcohol concentration test is conducted on the day of admission.

The sealing time of Lugol's solution was from 2 Days before treatment (Day-2) to 14 Days after treatment (Day14).

Results of all SPECT screening tests are valid within 7 days of screening and should be re-examined if the administration has not started for more than 7 days.

Table 2 Content of Assessment

Assessments	Contents
Physical examination	General appearance, the examination of the skin, neck (including thyroid), eyes, ears, nose, throat, lungs, heart, abdomen, superficial lymph nodes, limbs, and nervous system
Vital sign	Pulse, blood pressure, body temperature, breathing rate;
Urine drug abuse screening	Morphine, ketamine, methamphetamine, marijuana, ecstasy, K powder, amphetamine, etc.
Serology (HIV, HBV, HCV), RPR (syphilis)	HBsAg, HIV-Ab, HCV-Ab, RPR (syphilis)
Blood routine test	Red blood cell count, white blood cell count, neutrophil percentage, lymphocyte percentage, mononuclear cell percentage, platelet count, hemoglobin, etc.
Urine routine test	Urine PH, urine protein, urine glucose, urine ketone body, urine red blood cells, urine white blood cells, etc.
Blood biochemistry	Alanine aminotransferase (ALT), aspartate aminotransferase (AST), total bilirubin (TBIL), straight bilirubin (DBIL), total protein (TP), albumin (ALB), globulin, alkaline phosphate enzymes (ALP), lactate dehydrogenase, creatine kinase (CK), glucose (GLU), urea (BUN), blood creatinine (Cr), etc.
Myocardial enzyme	N-terminal-B-type natriuretic peptide precursor, troponin I, myoglobin, creatine kinase-MB subtype quality
Coagulation function	Prothrombin time (PT), thrombin time (TT), fibrinogen (FIB), activated part thrombin time (APTT)
SARS-CoV-2 Specific antibody detection	Anti-SARS-CoV-2 IgM, anti-SARS-CoV-2 IgG.

2. INTRODUCTION

2.1 Study Rationale

This clinical study will be conducted to evaluate the biodistribution of meplazumab in healthy subjects, thus providing a new macromolecule antibody drug for the treatment of COVID-19 pneumonia.

2.2 Background

Coronavirus is an enveloped positive-stranded RNA virus belonging to the family *Cornonaviridae*, order *Nidovirales*.[1] Over the past 20 years, two human coronaviruses, SARS-CoV and MERS-CoV, were reported to lead to severe and fatal lower respiratory tract infection.[2,3] In Dec 2019, an outbreak of a respiratory syndrome, COVID-19, was detected in China, which is caused by a novel coronavirus named SARS-CoV-2.[4] The genome sequence of SARS-CoV-2 shows it belongs to betacoronavirus genus and has extremely high homology with SARS-CoV at genome and proteome.[5,6]

Illness onset among rapidly increasing numbers of COVID-19 in China and the globe indicates that SARS-CoV-2 is more contagious than both SARS-CoV and MERS-CoV. The infection of SARS-CoV-2 leads to acute viral exudative pneumonia, with multiple organ damages, especially in the lung, presenting bilateral diffuse alveolar damage with cellular fibromyxoid exudates.[7] 80% of cases have mild symptoms (including non-pneumonia and mild pneumonia cases), while about 20% patients have developed severe pneumonia and acute respiratory distress syndrome, which attribute to death.[8] As of April 8th, 2020, 1,282,931 confirmed cases and 72,774 death had been reported globally in over 211 countries and areas.[9]

2.2.1. Meplazumab, a Humanized Anti-CD147 IgG2 Monoclonal Antibody

2.2.1.1 Description

Meplazumab (Ketantin®) is a lyophilized powder for injection of small volume. The main active ingredient of the product, meplazumab, is a humanized immunoglobulin (Ig) G2 mAb, consisting of the complementary-determining regions of anti-CD147 murine antibody and the human framework region. Meplazumab Injection is a novel humanized mAb, which was developed by the National Translational Science Center

for Molecular Medicine and Jiangsu Pacific Meinuoke Biopharmaceutical Co. LTD. Meplazumab is a recombinant human IgG_2 antibody expressed by CHO cells. We use bioinformatics and recombinant technology to replace the FR sequence in the light and heavy chain variable regions of the antibody with the human FR sequence. More than 2/3 of the whole molecule is the human source, in which the variable region has the function of binding antigen, while the constant region has the function of antibody effect, immunogenicity, and species characteristics. The Fc fragment of the chimeric antibody can prolong the half-life of the antibody in serum and theoretically reduce the immunogen of the heterologous antibody. Compared with its parent non-humanized MAb, the equilibrium dissociation constant of humanized antibody did not change significantly, and the affinity constant (K_D) was 1.7×10^{-10}M, which indicated that meplazumab and its parent mouse MAb 6H8 (affinity constant $K_D = 4.48 \times 10^{-10}$M) had a similar affinity, which ensured the binding ability of MAb to target molecules *in vivo* and *in vitro*.

2.2.1.2 Structure

Meplazumab is produced in Chinese hamster ovary cells using recombinant deoxyribonucleic acid (rDNA) technology. It has a typical antibody structure composed of 2 light chains and 2 heavy chains linked by interchain disulfide bonds. Each light chain comprises 214 amino acids and 2 intrachain disulfide bonds. Each heavy chain comprises 442 amino acids and 4 intrachain disulfide bonds (Figure 1 of the current Investigator's Brochure [IB] for meplazumab [5])

2.2.2 Preclinical Data

Preclinical studies and pharmacological studies have indicated that meplazumab for intravenous injection is safe and effective with well-controlled quality. The nonclinical studies of meplazumab include pharmacology, PK, and toxicology studies. Refer to Section 4 of the current IB for meplazumab for further details.

2.2.3 Pharmacology

In pharmacology studies, meplazumab's affinity to human- and nonhuman primate-derived CD147 and the mechanism of action of meplazumab was evaluated through an array of in vitro studies. Meplazumab affinity to non-human primate CD147 antigen

was approximately 10-fold lower (1.26×10^{-9} K_D in cynomolgus and 2.16×10^{-9} K_D in rhesus monkey) when compared with affinity to the human CD147 antigen. In vitro studies also support the mechanism of action of meplazumab. The concentrations of meplazumab at human CD147 RO% of 10%, 20%, 50%, and 90% were calculated to be 0.511, 0.820, 1.634, and 4.169 µg/mL, respectively. When the drug exhibited intravascular distribution, the drug dose at receptor occupancy level of 10%, 20%, 50%, and 90% at maximum concentration (C_{max}) was predicted to be 0.039, 0.063, 0.126, and 0.321 mg/kg, respectively. After in vitro incubation of meplazumab with rhesus monkey peripheral blood, the concentration of meplazumab at CD147 RO% of 10%, 20%, 50%, and 90% were calculated to be 1.280, 3.268, 12.264, and 51.057 µg/mL, respectively. The drug dose at RO% of 10%, 20%, 50%, and 90% at C_{max} were predicted to be 0.079, 0.203, 0.762, and 3.171 mg/kg, respectively.

The binding efficacy of CD147 to SARS-CoV-2 spike (RBD) protein (SP) was evaluated by SPR, Co-Immunoprecipitation (Co-IP), and ELISA. The interaction between CD147 and SP was validated, the affinity constant (K_D) of CD147 to bind to SP is 1.85×10^{-7} M, and half-maximal effect concentration (EC_{50}) was determined to be 68.83 µg/mL. The subcellular localization of CD147 and SP was observed in samples from SARS-CoV-2 virus-infected Vero E6 cells by Colloidal gold particles in double-labeling immunoelectron microscopy.

The binding efficacy of CD147 to CyPA was evaluated by SPR, Co-Immunoprecipitation (Co-IP), and ELISA. The interaction between CD147 and CyPA was validated, the affinity constant (K_D) of CD147 to bind to CyPA was 3.34×10^{-8} M, and half-maximal effect concentration (EC_{50}) was determined to be 135 µg/mL.

Meplazumab was found to block the interaction between CD147 and its ligands, SP and CyPA, with a half-maximal inhibitory concentration (IC_{50}) of 16.44 µg/mL and 1.28 µg /mL, respectively.

In vitro, functional studies evaluating the inhibitory effects of meplazumab on Vero E6 cells infected with SARS-CoV-2 (2019-nCoV/Beijing/AMMS01/2020) virus strain demonstrated that meplazumab effectively inhibited the infection of virus strain in a concentration-dependent manner. The median effective concentration (EC_{50}) for

cytopathic effect(CPE) was 35.98 μg/mL, and the median inhibitive concentration (IC_{50}) for virus gene copy number was 17.58 μg/mL, respectively.

The effect of meplazumab binding to CD147 and the oxygen-carrying/release properties of erythrocytes were also explored in vitro. Meplazumab at concentrations up to 25 μg/mL, did not affect the oxygen-carrying/release capacity of erythrocytes in vitro. However, meplazumab may promote the release of oxygen at concentrations ranging from 125 to 2000 μg/mL.

2.2.4 Pharmacokinetics

Tissue distribution and excretion of ^{125}I-meplazumab was studied in Sprague Dawley rats. At 240 hours after administration, the radiation concentration of ^{125}I-meplazumab was mainly distributed in plasma, whole blood, and hemocytes. Meplazumab did not pass through the blood-brain barrier and did not accumulate in skeletal muscle tissue. The ^{125}I-meplazumab metabolites were mainly excreted by the urine, and a small portion was eliminated in the feces.

2.2.5 Toxicology

Single-dose GLP (cynomolgus monkey) and 4-week GLP (cynomolgus and rhesus monkey) studies were conducted to evaluate the in vivo safety of meplazumab in nonhuman primates. Treatment with a single dose of meplazumab at up to 100 mg/kg did not cause deaths in cynomolgus monkeys. No obviously drug-related toxic effect was observed. The no observed adverse effect level (NOAEL) was considered to be 100 mg/kg in this study. In the repeat-dose study of meplazumab at doses of 2, 6, and 12 mg/kg, the significant changes of meplazumab on cynomolgus monkeys included: increased percentage reticulocyte count at ≥6 mg/kg doses, considered to be a pharmacodynamic effect. The above alterations were reversible. Under the conditions of this study, the NOAEL was considered to be at 12 mg/kg with corresponding $AUC_{(0-168)}$ of 19 123 g·h/mL in males and 11 830 g·h/mL in females.

Intravenous administration of meplazumab once weekly in rhesus monkeys for 4 weeks at doses of 4 or 20 mg/kg/week was well tolerated. The meplazumab-related changes were present in erythrocyte mass parameters (erythrocyte counts, hemoglobin, and hematocrit [HCT]) decreases, reticulocyte increases, and total bilirubin (TBIL) and

direct bilirubin (DBIL) increases. When given 20 mg/kg/week meplazumab, the changes of erythrocyte mass parameters, TBIL and DBIL were considered to be toxicologically significant. The NOAEL was considered to be 4 mg/kg with a corresponding $AUC_{(0\text{-}168)}$ of 2894 μg·h/mL in males and 3420 μg·h/mL in females.

Supporting in vitro tissue distribution, hemolysis, and antibody-dependent cell-mediated cytotoxicity assays complement the toxicology program. Meplazumab at the maximal intended clinical concentration of 2 mg/mL did not cause hemolysis or erythrocyte aggregation in rabbit, cynomolgus monkey, or human erythrocytes in vitro and was deemed suitable for injection in the clinic. Meplazumab over the concentration range of 0.0125 to 1 mg/mL did not induce an antibody-dependent cell-mediated cytotoxicity effect in vitro.

2.2.6 Clinical Data

The therapeutic effects of meplazumab against COVID-19 was investigated in an Exploratory clinical study. A prospective, single-center, open-labeled trial at Tangdu Hospital of Fourth Military Medical University in Xi'an, China. The study protocol and consent were approved by the Independent Ethics Committee of Institution for National Drug Clinical Trials at the Tangdu hospital. The study was registered at ClinicalTrials.gov (NCT04275245) before any patient enrollment.

Enrolled patients fulfilled inclusion and exclusion criteria. The inclusion criteria are as follows: men and women aged 18 to 78 years; patients with common, severe, or critical COVID-19 pneumonia were laboratory and clinically diagnosed according to *Diagnosis and Treatment for 2019 Novel Coronavirus Diseases* released by National Health Commission of China;[18] the subjects must understand the study and be willing to participate in the study. The exclusion criteria are as follows: allergic reactions or a history of allergy to any of the ingredients treated in this trial; patients not suitable to participate in this study by the judgment of the investigator. Each patient signed an informed consent form before enrollment.

Case severity of COVID-19 is categorized as common, severe, and critical by the vital signs, oxygenation index (PaO_2/FiO_2), chest radiographic, and vital organ function based on the *Diagnosis and Treatment for 2019 Novel Coronavirus Disease*.[18] Common

case was featured by fever, respiratory symptoms, and radiographic pneumonia. A severe case was characterized by any of the following signs: dyspnea, respiratory frequency ≥30/minute, blood oxygen saturation ≤93%, and PaO_2/FiO_2 ratio <300mmHg. The critical case was characterized by any of the following signs: respiratory failure needing mechanical ventilation, shock, and multiple organ dysfunction/failure needing Intensive Care Unit (ICU). The patients who met all the following criteria will be discharged: body temperature recovered and remained normal more than three days; respiratory symptoms relief, and two continuous negatives for nasopharyngeal swab test for SARS-CoV-2 (interval more than 24 hours).

Seventeen patients with COVID-19 pneumonia were administrated with meplazumab treatment. 10mg meplazumab was administered on day 1, day 2, and day 5 by intravenous infusion within 60–90 min. Efficacy and safety were assessed at baseline, every day after day 1 to day 14, and every week thereafter up to day 28 or discharge.

In the same period, hospitalized patients in the same center were observed as concurrent control and were required to follow the inclusion and exclusion criteria mentioned above.

- Recovery and Discharged Meplazumab treatment improved the discharged rate of severe and critical cases significantly compared to the control group (p=0.005). No discharged cases were observed over the follow-up period in the control group. On day 28, 4 severe cases and 1 critical case were improved to common, and no case was discharged in the control group. In the meplazumab group, 9 cases (6 severe and 3 critical) were discharged, 2 critical cases were improved to common, and 1 critical case was improved to severe, demonstrating a significantly beneficial outcome compared to the control group (p=0.021). These results indicated that meplazumab treatment accelerated the improvement and made a rapid recovery from COVID-19 pneumonia, especially for the severe and critical cases.
- The chest radiographic The meplazumab group showed more significant benefit than the control group on days 7, 14, and 21 (p=0.010, p=0.006, and

p=0.037, respectively), which was coincident with the improvement of case severity.

- The virus clearance At day 7, the rate of virus nucleic acid negative conversion in the control group was 27.3% (3/11) and reached 54.4% (6/11) on day 14. While, the rate was 76.5% (13/17) in the meplazumab group on day 7, and reached 94.1% (16/17) on day 14, which were significantly higher than the control group (p=0.019 and p=0.022, respectively). The analysis of time to virus-negative indicated that meplazumab-treated patients converted to negative in a shorter period than patients in the control group significantly (median 3, 95%CI [1.5–4.5] vs. 13, [6.5–19.5]; p=0.045, HR=0.374, 95%CI [0.143–0.978])).
- Lymphocytopenia count In the observation period (day 7 to 28), the percentages of patients with a normal lymphocyte count (>0.8×10^9/L) were increased in both groups, while the improvement in the meplazumab group was more notable. Compared to the baseline, the percentage in the meplazumab group was improved significantly as early as day 7 (p=0.031), but no significant difference was detected in the control group over the follow-up period.
- CRP The concentration of CRP was also measured as a predictor of COVID-19 severity. From day 3 to day 28, the percentages of patients with a normal CRP concentration (≤5mg/L) were increased from 52.9% (9/17) to 82.4% (14/17), indicating significant increases compared to baseline (all p<0.05 at days 3, 7, 14, 21, and 28). In the control group, significant increases were observed at day 14 and day 21 compared to baseline. The data suggest that meplazumab exhibited an effect on the control of virus-induced acute inflammation at early management.
- Safety and tolerance No other adverse event was reported in meplazumab-treated patients, including irritation around the injection site, rash, nausea, vomit, anemia, neutropenia, thrombocytopenia, total bilirubin, albumin, and creatinine, etc.

All above, Meplazumab efficiently improved the recovery of patients with SARS-CoV-2 pneumonia with a favorable safety profile.

2.3 Benefit/Risk Assessment

As this study will be conducted on healthy human subjects, a health benefit to the subject is not anticipated.

The clinical study protocol has been designed such that the risk to subjects in this study will be minimized by adequate selection of eligibility criteria and schedule of clinical monitoring, in-house observation, administration, and treatment duration. The Sponsor will immediately notify the Principal Investigator if any additional safety or toxicology information becomes available during the study.

This study will be performed in compliance with the protocol, International Council for Harmonisation (ICH) Good Clinical Practice, and applicable regulatory requirements.

2.4 Meplazumab

Based on the nonclinical safety profile and the expected high efficacy against COVID-19 pneumonia, it is anticipated that meplazumab will have a positive benefit-risk profile to warrant studies in healthy volunteers if conducted under well-controlled conditions, including in-house confinement periods, extensive monitoring, and protective measures for contraception.

Meplazumab has not yet been evaluated in humans in controlled clinical studies. Thus, information regarding expected adverse events (AEs) from controlled clinical studies is unknown. Based on the available nonclinical data to date, meplazumab did not cause serious toxicity and side-effects on the tested animals. Therefore, the conduct of the study is considered justifiable.

Based on the nonclinical data, possible adverse reactions from meplazumab treatment include:

- Changes in erythrocyte mass parameters
- Increases in TBIL, DBIL, and alanine aminotransferase (ALT)
- Possible off-target cross-reactivity on some of the CD147 on other organs

Potential risks of a foreign protein such as a therapeutic antibody may include the administration or immune reactions, including hypersensitivity, injection-site reactions, and immunogenicity. Subjects could potentially develop ADAs that may be neutralizing and may be associated with allergic or anaphylactic toxicity or induce or enhance meplazumab toxicity. Standard clinical assessments and interventions are recommended for allergic or anaphylactic reactions. Subjects will be monitored for these events and ADA formation in this first-in-human (FIH) study.

More detailed information about the expected benefits and risks and reasonably expected AEs of meplazumab may be found in the IB.

3 OBJECTIVES AND ENDPOINTS

Table 3 Study Objectives and Endpoints

**Objectives**	**Endpoints**
Primary	
• To evaluate the biodistribution of ^{131}I-Meplazumab in tissues	• The radiation ratio of ROI of tissues to cardiac blood-pool (T/C)
Secondary	
• To evaluate the pharmacokinetics of ^{131}I-Meplazumab	• The pharmacokinetics parameters of ^{131}I-Meplazumab in blood and urine

4 STUDY DESIGN

4.1. Overall Design

The biodistribution study is a single-center study. One cohort is planned, 3 or more subjects receiving 5 mCi or 10 mCi ^{131}I-mepolizumab by single intravenous infusion. The subjects will be conducted general condition, medical history, medication history, physical examination, vital sign measurement, drug abuse screening, blood, urine, liver and kidney function, blood coagulation function, electrocardiogram, and other related tests during the screening period (-14～-2d). If the subjects met the inclusion criteria, Lugol's solution was taken orally 48 hours before treatment (Day-2), 0.5mL/time, 3 times a day (meal at the same time) until 14 days after treatment. On the third day (Day0), the subjects examined the iodine uptake rate on an empty stomach in the morning. If the iodine uptake rate results showed that the ^{131}I uptake was between 8.5% and 17%, then 5 mCi^{131}I-meplazumab was injected; if the iodine uptake rate showed that the ^{131}I uptake was less than 8.5%, the injection dose could be increased to 10 mCi. The mode of administration was a single intravenous drip, which was finished at 60 ±5min.

Standard meals were provided 4 hours after administration. According to the test flow chart, SPECT scans (including heart, lungs, spleen, liver, kidney) were performed at different time points, and blood and urine samples were collected at the same time. 336 hours after administration (Day14), the subjects left the research center temporarily after collecting blood and urine samples, and one month after administration (Day30), they returned to the research center to collect blood samples and complete the related tests to complete the pharmacokinetic experiment. At 1 and 3 months after administration, the subjects were required to draw blood to check eight items of thyroid function to determine whether hypothyroidism occurred.

Aspects of study design that may be adapted are presented in. Table

Table 4 Adaptive features in the study

Category	Adaptive features	Boundaries
Dose levels	The dose of ^{131}I-meplazumab for biodistribution study would be determined by the results of single-dose study.	The preset dose for administration is 0.3mg/kg (5 mCi or 10 mCi)
Study schedule	Confinement period may be extended beyond Day 7 at the investigator's discretion based on emerging AE and the results of biodistribution.<br>The time to EOS (currently Day 90±2) may be extended at the investigator's discretion to allow for appropriate assessment of biodistribution and PK data.	Confinement in study center will not exceed Day 7, unless medically indicated.<br>The maximum window to EOS visit will not exceed Day 90 (±2 days), unless medically indicated.
Assessments	Timing of SPECT scanning and PK blood sampling may be adjusted based on emergent biodistribution and PK results. This will be decided by the principal investigator based on available data.	In accordance with evolving data up to the decision-making time point.
	Additional safety blood and/or urine samples may be taken if the principal investigator considers it necessary from a safety/tolerability point for an upcoming dose cohort.<br>At the investigator's discretion, additional or fewer exploratory assessment samples may be taken in accordance with emerging data.	The maximum volume of blood collected will not exceed 270 mL in a 30-day period.<br>Efforts will be taken to ensure the subject's safety is not compromised.

PK = pharmacokinetics.

4.2. Scientific Rationale for Study Design

The biodistribution of meplazumab in healthy subjects is a single-center phase I clinical trial of ^{131}I-meplazumab in healthy volunteers. The rationale for the various aspects of this study design is described below. The ^{131}I-meplazumab dose is provided in Section 4.6.

4.3. Study Population

While the intended patient population for meplazumab is people infected with COVID-19 pneumonia, this clinical study will be performed in healthy subjects to characterize the biodistribution of the compound in humans in the absence of any disease-related and potentially confounding factors. The inclusion criteria were chosen to help ensure the inclusion of only healthy subjects. Additional criteria are in place to ensure the safety and wellbeing of subjects.

4.4. Study Design

Biodistribution study will explore the distribution of ^{131}I-meplazumab in vital organs or tissues, including lung, liver, heart, spleen, kidney, whole blood, and urine, at multiple time points post-dose. For minimizing the potential risk, Lugol's solution will take orally 48 hours before treatment (Day-2), 0.5mL/time, 3 times a day (meal at the same time) until 14 days post-dose. Three healthy volunteers were enrolled in this study. The subjects will determine the activity of drug administration according to the results of iodine uptake rate. If the iodine uptake rate shows that ^{131}I uptake is between 8.5% and 17%, then inject 5 mCi ^{131}I-meplazumab the iodine uptake rate shows that the ^{131}I intake is less than 8.5%, the injection dose can be increased to 10 mCi. The mode of administration was a single intravenous drip, which was finished at 60 ±5 min. SPECT planar imaging was performed at 15 or more time points post-dose to investigate the distribution of meplazumab in vital organs. Blood and urine samples will be collected, and the radioactivity-time curve and calculate pharmacokinetic parameters with DASver1.0 (Drug and Statistics for Windows) pharmacokinetic program.

4.5. Justification for Dose of Meplazumab

The starting dose in the trial was selected considering the results of safety, tolerability PK and RO% analysis in phase I FIH clinical trial, non-clinical pharmacokinetics, and exploratory phase II clinical studies, combined with the administration method and mechanism of the drug.

The mechanism of action of this product is to inhibit virus invasion into host cells by blocking CD147-spike protein interaction and inhhibiting inflammatory storm syndrome response by blocking CD147 - CyPA interaction

The study of biodistribution, meplazumab for ^{131}I labeled injection can diiodide physiologically, and free iodine [^{131}I] will concentrate in the thyroid gland in the body, which may cause hypothyroidism. The incidence and extent of hypothyroidism were associated with an individual's sensitivity to radiation. For reducing the potential risk of hypothyroidism, volunteers were given Lugol's solution two days before and two weeks after treatment to maximize thyroid protection.

According to *Radiation oncology* (fourth edition, editor-in-chief of Xianzhi Gu), the maximum tolerated dose of ^{131}I in the thyroid is 45 Gy. Jianhua Geng, a researcher from the Department of Nuclear Medicine, Cancer Hospital, Chinese Academy of Medical Sciences, estimated that when the thyroid was blocked by 10% and the dosage was 10 mCi, and 5 mCi, the radiation doses of ^{131}I to the thyroid were 53.28 Gy and 26.64 Gy, respectively. Thus, through calculation, we obtained the safety limit of 10 mCi and 5 mCi, and the thyroid closure rate was less than 8.5% and 17%, respectively. The iodine absorption rate will be measured after subjects take Lugol's solution for 48 h. If the iodine absorption rate shows uptake of ^{131}I between 8.5-17%, 5 mCi ^{131}I-meplazumab (0.3 mg/kg) will be injected. If the iodine absorption rate shows uptake of ^{131}I of less than 8.5%, the injection dose can be increased to 10 mCi.

4.6. End of Study Definition

The EOS is defined as the date of the last visit of the last subject in the study. The sponsor will notify all study units of the end time of the study. After this time, the study must be approved by the sponsor and can be implemented without supplement.

4.7. Study Stopping Criteria

The sponsor reserves the right to terminate the study at any time. The study will be stopped include but not limited to:

- The investigator cannot follow the trial protocol or GCP guidelines;

- Any security concern;
- Evidence to suggest a lack of therapeutic effect;
- The investigator did not recruit enough subjects.

If any of the criteria for stopping dose escalation are met in any cohort of study, the study will be stopped. This decision will be made by the sponsor.

5 STUDY POPULATION

Prospective approval of protocol deviations to recruitment and enrollment criteria, also known as protocol waivers or exemptions, is not permitted.

5.1.Inclusion Criteria

Subjects are eligible to be included in the study only if ALL of the following criteria apply:

1. 18≤ age ≤50, regardless of gender;
2. Weight: normal subjects should generally be≥50 kg, body mass index (BMI= weight/height 2 (kg/m^2)) should be in the range of 19.0~26.0 (including both ends), avoid overweight or underweight;
3. When screening, the results of vital signs, physical examination, laboratory examination (blood routine, urine routine, blood biochemistry, coagulation, thyroid function, etc.), and ECG examination should be within the normal range, or beyond the normal range, but was judged as "no clinical significance (NCS)" by researchers.
4. No tobacco, alcohol, and other bad habits (drinking is referred to the amount of alcohol drinking women one day more than 15 g, men over 25 g (15 g of alcohol which is equivalent to 150 mL wine or 450 mL beer, 50 mL low-alcohol liquor)) more than twice a week, a heavy smoker refers to smoke five cigarettes a day or more), and there is no history of drug abuse (defined as the use of illicit drugs);
5. Have no family planning during the trial and within 6 months after the completion of the trial, and are willing to use nonhormonal contraceptives;
6. Have the ability to normal communication with medical staff and abide by the relevant management regulations of the hospital;

Understand the research procedures and methods, participate in the test voluntarily, sign the informed consent in written form, and be willing and able to comply with all tests' requirements.

5.2.Exclusion Criteria

Subjects are excluded from the study if ANY of the following criteria apply:

- Allergic to or having a history of allergy to drugs or any of its components, or having a history of more serious allergic reactions to drugs, food, proteins (asthma, rubella, eczema dermatitis, etc.);
- Vital signs, physical examination, routine laboratory examination (blood routine, urine routine, blood biochemistry, coagulation, thyroid function, etc.), 12 lead ECG and other examination abnormal and clinically significant;
- The novel coronavirus specific IgM and IgG antibodies were positive;
- Fever (body temperature ≥ 38.0 ℃ occurred within 3 days before medication;
- Pregnant or lactating subjects;
- Has received or is participating in other clinical trials within 3 months prior to the evaluation;
- Those who have been diagnosed or suspected to have immune deficiency or autoimmune diseases, who have received immunosuppressive therapy such as anticancer chemotherapy or radiotherapy, or who have received systemic corticosteroid therapy in the past 6 months.
- Those who have a history of dizzy needles；
- Those who are positive for hepatitis B surface antigen, hepatitis C antibody, HIV antibody, and syphilis antibody；
- The subjects drank more than 25g (male) / 15g (female) in one day. 15g equivalent to 450mL beer, 150mL wine or 50mL low spirits) or excessive caffeinated beverages (> 4 cups/day) more than twice a week, excessive smoking (> 5 cigarettes/day), or those who cannot stop smoking and drinking during the trial；
- Those who had participated in blood donation or blood loss ≥ 400mL in the previous 3 months were screened.；
- The researchers judged that there were other reasons that the subjects were not suitable to participate in this study.

5.3.Enrollment

Subjects will be recruited from the clinical site database or by a general or study-specific advertisement via print, radio or poster media to the general community, as approved by the independent ethics committee. No restrictions will apply to ethnic or racial categories.

Subjects will be compensated for the time that they spend participating in the study using a formula determined by the study site.

Screen Failures

Screen failures are defined as subjects who consent to participate in the clinical study but who do not meet the eligibility criteria and are not subsequently entered into the study. A minimal set of screen failure information is required to ensure transparent reporting of screen failure subjects to meet the Consolidated Standards of Reporting Trials publishing requirements and to respond to queries from Regulatory Authorities. Minimal information includes ICF and screening visit dates, demography, screen failure details, eligibility criteria, and any SAE.

6 STUDY TREATMENT

Study treatment is defined as an investigational treatment(s), non-investigational treatments, marketed product(s), placebo, or medical device(s) intended to be administered to a subject according to the study protocol. All study treatments will be packaged and labeled in accordance with all applicable regulatory requirements, including the Access to Unapproved Therapeutic Goods.

6.1.Study Treatment(s) Administered

Meplazumab for injection will be provided in the form of a 10-mg/vial freeze-dried powder containing 1.60 g histidine, 3.08 g histidine hydrochloride, 50.0 g sucrose, 70.0 g mannitol, and 1.0 g polysorbate 80.

For biodistribution study, ^{131}I-meplazumab should be prepared as follows:

- ***Composition of iodine [^{131}I] meplazumab labeling kit***

  No. I: phosphate buffer

  No.II: freeze-dried meplazumab

  No. III: N-bromosuccinimide (NBS)

  No. IV: human serum albumin

  No.V: PD-10 purification column

  No. VI: 0.2 μ m disposable needle filter

  Note: iodine [^{131}I] sodium solution for labeling, no reducing agent, radioactive concentration 500～2000mCi/mL, ordered before use.

- ***Marking***

  Under aseptic operating conditions, proceed in the following order.

  (1) Reliable shielding protection is used in operation.

  (2) The 0.1m phosphate buffer (bottle I) 0.5ml was added to the monoclonal antibody (bottle II) to completely dissolve it.

  (3) Add 0.1m phosphate buffer (bottle I) 1ml to NBS (bottle III) to make it completely dissolve.

  (4) The 0.1m phosphate buffer (bottle I) 0.5ml was added to human serum albumin (bottle IV) to completely dissolve it.

(5) Extract the given dose of $Na^{131}I$ and add it to the monoclonal antibody (bottle II) that has been dissolved in phosphate buffer.

(6) Extract 60-100μl of NBS (bottle III), which has been dissolved in phosphate buffer, and add it to bottle II and mix well for 60 seconds.

(7) The 0.5ml of human serum albumin (bottle IV), which has been dissolved in phosphate buffer, is added to bottle II.

- ***Purification method***

  Proceed in the following order

  (1) Balancing PD-10 purification column with 20ml normal saline.

  (2) Put the 2ml marker on the column, after the marker has completely entered the column, connect the purification column (V), the needle filter (IV), and the 7 gauge needle (self-provided), and tighten it.

  (3) Add 5ml saline to the column.

  (4) Insert the connector into the vacuum bottle (99mTc elution bottle). When the liquid in the bottle reaches 5ml, the liquid in the vacuum bottle is the finished product.

  *The eluent in this process is discarded (non-radioactive).

- ***Radioactive waste treatment***

  Dispose of discarded vials, needles, and syringes in accordance with the National Health Protection Standard for Medical radioactive waste Management (GBZ133-2002).

6.2.Preparation/Handling/Storage/Accountability

6.2.1 Preparation, Dosage, and Administration

^{131}I-Meplazumab

Seal thyroid gland: Lugol's solution was taken orally 2 days before treatment, 0.5ml/ times, 3 times a day (take it at the same time after eating), to 14 days after treatment (Day-2 to Day14).

The mode of administration is intravenous infusion. Before administration, first,

dissolve the drug with 1mL aseptic injection water, extract the required amount of solution, add 0.9% sterile saline 100mL. Use within 2 hours after preparation. The drug was injected slowly intravenously, and the administration was completed in 60 ±5min. The process of administration should be kept away from light.

6.2.2 Handling, Storage, and Accountability

^{131}I-Meplazumab

In order to ensure the safety of drugs, the experimental drugs shall be taken care of by special personnel, kept in a unified manner according to the drug storage conditions, kept away from light and sealed at 2~8°C, and fill in the records of drug use and the temperature and humidity records of the drug storage environment. And make detailed records of its receipt, preservation, collection, and return.

6.3.Measures to Minimize Bias: Randomization and Blinding

No randomization or blinding in this study.

6.4.Study Treatment Compliance

The prescribed dosage, timing, and mode of administration of study treatment may not be changed, except as defined in Section 6.6 and the Schedule of Assessments. Any departures from the intended regimen must be recorded in the eCRF.

The ^{131}I-Meplazumab will be administered at the clinical unit in the presence of clinical unit staff.

6.4.1 Treatment Strategy

The clinical staff is responsible for the ongoing safety and wellbeing of the subjects while they are in the study center. There is a paging system to alert the clinical staff to any area in the center where a subject may need medical attention. In the case of an emergency, cardiac resuscitation trolleys are found in the main ward areas of the study center. These trolleys contain drugs, equipment for airway insertion, circulation lines, defibrillation, etc., together with oxygen cylinders and portable suction machines. There is a physician on-site 24 hours a day. In addition, if necessary, the clinical staff can contact further on-call physicians or public emergency services in the event of a

serious medical event. Equipment and emergency drugs are available to treat common medical emergencies that might occur in a Phase I study.

6.4.2 Warnings and Precautions

All effects cannot be reliably predicted. The preclinical data suggest an acceptable safety margin. Facilities and staff for resuscitation and the treatment of other medical emergencies will be provided.

6.5.Prior and Concomitant Therapy

Prior medications, treatments, and procedures are those occurring prior to IMP dose or inoculation. Concomitant medications from hospitalization to Day 90 will be recorded.

At each visit, subjects will be questioned in relation to their drug intake since their previous visit. Any medication or vaccine (including over-the-counter or prescription medicines, vitamins, and/or herbal supplements) that the subject is receiving at the time of enrollment or receives during the study must be recorded on the eCRF along with:

- Reason for use.
- Dates of administration, including start and end dates.
- Dosage information, including exact dose and timing.

The Medical Monitor should be contacted if there are any questions regarding concomitant or prior therapy.

6.5.1 Prohibited Medications

Subjects must abstain from taking any unnecessary drugs, including prescription or nonprescription drugs, before the start of study treatment until completion of the study. Unless, in the opinion of the Investigator and Sponsor, the medication will not interfere with the study.

6.5.2 Permitted Medications

Based on the premise of protecting the subjects' interests and safety, the investigator can decide whether to use other concomitant drugs during the study period, which did not affect the study drug's evaluation. All concomitant medication

information and symptomatic treatment received by the subjects should be recorded in the eCRF.

6.6.Dose Modification

The details of dose selection are described in Section 4.5, and study stopping criteria in Section 4.7 of this protocol.

6.7.Treatment After the End of the Study

In this study in healthy subjects, no further treatment or medical care is planned or required after the EOS visit.

7 STUDY ASSESSMENTS AND PROCEDURES

7.1 Schedule of Assessments

- For the study, study procedures and their timing are summarized in the Schedule of Assessments.
- Protocol waivers or exemptions are not allowed.
- Immediate safety concerns should be discussed with the Sponsor immediately upon occurrence or awareness to determine if the subject should continue or discontinue study treatment.
- Adherence to the study design requirements, including those specified in the Schedule of Assessments, is essential and required for study conduct.
- The maximum amount of blood collected from each subject over the duration of the study, including any extra assessments that may be required, will not exceed 270 mL in any 30-day period. Repeat or unscheduled samples may be taken for safety reasons or for technical issues with the samples.

7.2 Screening Examinations

All subjects will undergo a screening examination to evaluate their health status. This examination will be conducted not more than 28 days prior to the planned IMP. Only subjects who meet the eligibility criteria will be enrolled in the study.

Written informed consent must be obtained before any study-related procedures and/or assessments are performed. The assessments to be performed at screening are indicated in the Schedule of Assessments. Subjects should have fasted for at least 8 hours prior to the safety laboratory assessments at the screening. Safety laboratory assessments with results outside of the normal laboratory range may be repeated once.

Subjects who fail to meet the protocol-specified inclusion- or exclusion criteria or who withdraw their consent in the Screening period are considered screening failures. The investigator will maintain a screening log to record details of all subjects screened and to confirm eligibility or record reasons for screening failure, as applicable.

7.3 Treatment Period

A review and update of the subject's eligibility criteria to ensure the subject remains eligible for participation in the study. Screening should be performed on Day -1 on admission to the study site and Day 1 before IMP administration and will be discharged on the morning of Day 14. The confinement periods during the study are subject to change for operational reasons. The assessments to be performed at specific time points during the treatment period are indicated in the Schedule of Assessment.

7.4 Outpatient Visits and Phone Calls

Ambulatory visits will be performed at regular intervals from Day 12 onwards. The assessments to be performed at specific time points during outpatient visits are indicated in the Schedule of Assessments.

7.5 End of Study/Early Termination Visit

The EOS visit is to verify that all values tested at screening have remained within a clinically acceptable range. The relevant tests will be performed on Day 90 (± 2 days). The assessments to be performed at this time point are indicated in the Schedule of Assessments. Unacceptable values and AEs will be followed up until they return to baseline/have resolved, or there is an adequate explanation that is not related to the study. The subject may be referred for appropriate counseling or for follow-up tests to a general practitioner or medical specialist as appropriate.

7.6 Demographics and Other Baseline Characteristics

At Screening, the following demographic data will be collected: age, and year of birth, sex (gender), race, and ethnicity.

Furthermore, the following will be documented:

- Social history including recreational drug use, alcohol intake, and tobacco use
- Female status (WOCBP, woman of non-childbearing potential, postmenopausal, sterilization)

7.7 Medical and Surgical History

The medical history will be elicited at Screening as described below. Information regarding any new medical conditions or illnesses will be elicited on Day -1.

Recording of past medical/surgical history will include:

- History of all known allergies
- History of substance abuse and recreational drug use
- History of depression, anxiety, mental illness, emotional problems, use of psychiatric medications, and previous psychotherapy
- Surgical procedures and results
- Any other current or past medical conditions

7.8 Efficacy Assessments

Not applicable.

7.9 Biodistribution Assessments

The biodistribution of ^{131}I-Meplazumab were assessed based on SPECT scanning. The parameters of SPECT scanning as follows:

Acquisition parameters: high-energy general-purpose collimator, acquisition speed 13cm/min, automatic tracking technology of body surface contour;

Collection time points: 1h, 2h, 8h, 12h (Day 0), 24h (Day 1), 36h, 48h (Day 2), 60h, 72h (Day 3), 84h, 96h (Day 4), 120h (Day 5), 144h (Day 6), 168h (Day 7), 192h (Day 8) after injection of ^{131}I-Meplazumab for whole-body SPECT scanning, a total of 15 acquisition time points (If the signal of two consecutive measurement points is not detected, it will be terminated collection; if the signal is still detection on Day 8, the investigator will make a plan and continue to monitor until Day 14).

Draw the regions of interest (ROI) of the heart, liver, lungs, spleen, kidneys, and thyroid on the imaging chart, and measure the radioactive count rate of each ROI.

The tissue uptake rate in the subject is calculated according to the subject's dose and the detection efficiency of the SPECT instrument used, and the result is expressed as the percentage (%ID) of the tissue uptake radioactivity in the dose.

The inspection time should be recorded accurately. Check at the time point specified in the Schedule of Assessments.

7.10 Pharmacokinetics

Collection of Blood Samples

Venous blood samples will be collected for measurement of serum concentrations of meplazumab at time points specified in the Schedule of Assessments. Collection of blood samples: before administration and 15min, 30min, 1h, 1.5h, 2h, 4h, 8h, 12h, 24h, 48h, 72h, 96h, 120h, 144h, 168h, 192h, 240h, 336h after administration respectively

The subject's cubital venous blood was 2 mL, of which 1 mL of whole blood was transferred to blood collection tubes containing anticoagulant. After the sample was collected, 1 mL was mixed, and the radioactivity count rates (min-1) in whole blood, plasma, and blood cells were measured.

Draw the blood radioactivity-time curve, and use the DAS ver1.0 (Drug And Statistics for Windows) pharmacokinetic program to calculate the pharmacokinetic parameters. Keep the samples away from light during sample collection and processing.

Collection of Urine Samples

Urine sample collection: Urine was collected every 8 hours within 24 hours after administration, and then collected every day, until the 8th day, and then on the 10th and 14th day. The radiochemical purity of iodine [^{131}I] was used to identify the components of radioactive substances in the urine. After quantification of each time period, the urine was mixed, and 1ml was taken to measure the radioactivity count rate (min-1). Calculate the percentage of urine radioactivity in the injected dose (ID%) at each time period to analyze the kinetic characteristics of radioactive material clearance in the urine.

All sample processing procedures, including the collection time of each sample and the date when the blood sample was shipped to the analyst, must be recorded in

detail. Any changes in the timing or addition of time points for any planned study assessments must be documented and approved by the relevant study team member and then archived in the Sponsor and study center study files but will not constitute a protocol amendment. The HREC will be informed of any safety issues that require alteration of the safety monitoring scheme or amendment of the ICF.

7.11 Adverse Events

Adverse events can be spontaneously reported by the subject, observed by the Investigator (either directly or by laboratory or other assessments), or elicited by general questioning.

The Investigator and any designees are responsible for detecting, documenting, and recording events that meet the definition of an AE, SAE, or Suspected Unexpected Serious Adverse Reaction (SUSAR), and remain responsible for following up AEs that are serious, considered related to the study treatment or study procedures, or that caused the subject to discontinue the study.

7.11.1 Time Period and Frequency for Collecting AE and SAE Information

For the study, all AEs/SAEs will be recorded from the time of signing of the ICF until the EOS/ET visit (Section “Schedule of Activities”). The method of recording, evaluating, and assessing the severity and causality of AEs and SAEs and the procedures for completing and transmitting SAE reports are provided in Appendix 3.

All SAEs should be recorded since signing the ICF to study completion on the SAE Form in the eCRF. All SAEs should be submitted to the Sponsor within 24 hours of site awareness. The Investigator will take immediate appropriate action in response to SAEs to ensure subject safety and attempt to identify the cause/s of the event.

The Investigator will also notify the Medical Monitor of any SAE within 24 hours of becoming aware of the event. The Investigator will submit any updated SAE/ data to the same recipients as the initial report within 24 hours of it being available.

Investigators are not obligated to actively seek AE, SAE after the conclusion of the study participation. However, if the Investigator learns of any SAE, including death, at

any time after a subject has been discharged from the study, and the Investigator considers the event to be reasonably related to the study treatment or study participation, the Investigator must promptly notify the Sponsor.

7.10.2 Method of Detecting AEs and SAEs

Care will be taken not to introduce bias when detecting AEs and/or SAEs. Open-ended and non-leading verbal questioning of the subject is the preferred method to inquire about AE occurrences.

7.10.3 Follow-up of AEs and SAEs

After the initial AE/SAE report, the investigator must follow each subject at subsequent visits/contacts proactively. All SAEs should be followed until resolution, stabilization, explained otherwise, or lost to follow-up. Further information on follow-up procedures is given in Appendix 3.

7.10.4 Regulatory Reporting Requirements for SAEs

Prompt notification by the investigator to the sponsor of an SAE is essential so that legal obligations and ethical responsibilities toward the safety of subjects and the safety of a study treatment under clinical investigation are met.

The sponsor has a legal responsibility to notify both the local regulatory authority and other regulatory agencies about the safety of a study treatment under clinical investigation. The sponsor will comply with country-specific regulatory requirements relating to safety reporting to the regulatory authority and Investigators.

Investigator safety reports must be prepared for SUSARs according to local regulatory requirements and Sponsor policy and forwarded to Investigators as necessary.

An Investigator who receives an Investigator's safety report describing an SAE or other specific safety information (e.g., summary or listing of SAEs) from the Sponsor will review and then file it along with the IB and will notify the HREC, if appropriate according to local requirements.

7.12 Pregnancy

- Details of all pregnancies in female subjects and female partners of male subjects will be collected after the start of study treatment and until 3 months after dosing (or 5 terminal half-lives, whichever is longer).
- If pregnancy is reported, the Investigator should inform the Sponsor within 24 hours of learning of the pregnancy.
- Abnormal pregnancy outcomes (e.g., spontaneous abortion, fetal death, stillbirth, congenital anomalies, ectopic pregnancy) are considered SAEs.

8 STATISTICAL CONSIDERATIONS

8.1 Statistical Hypotheses

No formal statistical hypothesis testing will be performed.

8.2 Sample Size Determination

Given the exploratory nature of this study, the sample size in this clinical trial is not based on formal statistical calculations but is considered adequate to characterize the distribution of the planned endpoints. In the biodistribution study, one cohort with 3 or more subjects is expected to be sufficient to meet the objectives of this study.

8.3 Populations

For purposes of analysis, the analysis sets in are defined as follows.

**Analysis Set**	**Description**
Entered Analysis Set	All subjects who sign the ICF.
Enrolled Analysis Set	All subjects who have met all eligibility criteria. This set will be used for subject disposition.
Biodistribution Analysis set	All subjects who have received a single dose of ^{131}I-meplazumab and have at least 1 SPECT scanning result collected post-dose without significant protocol deviations/violations or events thought to affect the scanning significantly.

ICF = informed consent form.

8.4 Statistical Analyses

The following sections describe the statistical analysis as it is foreseen when the study is being planned. A detailed Statistical Analysis Plan (SAP) will be developed and finalized before database lock and will describe the subject analysis sets to be included in the analyses and procedures for accounting for missing, unused, and spurious data. The SAP will also provide the format of listings and tables to be provided for completion of the Clinical Study Report (CSR). Any deviations from the SAP will be described and justified in the final CSR. This section is a summary of the planned statistical analyses of the primary, secondary, and exploratory endpoints.

All statistical analyses, summaries, and listings will be performed using SAS® software (Version 9.4 or higher).

The following descriptive statistics will be used as applicable to summarize the study data unless otherwise specified:

- Continuous variables: sample size [n], mean, standard deviation [SD], median, minimum [min], and maximum [max].
- Categorical variables: frequencies and percentages.

The baseline will be defined as the last variable, valid, non-missing assessment (scheduled or unscheduled) prior to IMP dosing.

Individual subject data will be presented in listings.

8.5 Study Population Data

Disposition including the total number of subjects randomized and treated in the study; the total number of subjects who complete the study; and the number of subjects that prematurely discontinue from the study, along with the reason for premature discontinuation, will be summarized by treatment group and listed for all enrolled subjects. The number and percentage (%) of subjects in each analysis set will also be presented.

Subject demographic data will be summarized by descriptive statistics. Medical history, current medical conditions, prior and concomitant medications, results of laboratory screening tests, drug and alcohol screening tests, and any other relevant baseline information will be listed by subject and cohort. The medical history will be listed by subject and coded using the latest version of the Medical Dictionary for Regulatory Activities (MedDRA). Prior and concomitant medications per the World Health Organization-Drug dictionary Reference List (WHO-DD) will also be recorded on the eCRF.

8.6 Biodistribution Analyses

All biodistribution analyses will be performed on the Biodistribution Analysis Set.

Biodistribution data will be summarized using applicable descriptive statistics and listed separately for each cohort of the study. Doctors should record in detail whether

they take action on adverse events, including no action, temporary interruption of research, taking accompanying drugs, use of non-drug treatment, and hospitalization. This product is a biological agent, and anaphylactic shock may occur in the study. In the event of a similar adverse event, our rescuers will, according to the emergency treatment plan for anaphylactic shock, rescue and treatment of the subjects, the corresponding treatment costs and compensation costs borne by the applicant. In addition, this product is a radioactive ^{131}I drug, may cause hypothyroidism. Subjects need to be given 1 month and 3 months after testing thyroid function indicators. In the event of hypothyroidism, the subject shall take Eugenol® (Levothyroxine sodium tablets) according to the condition, and the corresponding examination fee and the drug fee shall be borne by the applicant.

8.7 Safety analyses

All AEs occurring over the study period (including pre-IMP or pre-inoculation AEs) and treatment-emergent AEs (TEAEs) will be summarized by treatment (dose cohort), severity.

Adverse events will be coded using the Medical Dictionary for Regulatory Activities. The overall number and percentage of subjects with at least one AE (and SAE) will be tabulated over the entire study period. All AE data will be summarized by treatment received and study period. For each study treatment and study period, the frequency of TEAEs will be tabulated by preferred term and system organ class. Treatment-emergent AEs by maximum severity, TEAEs by relationship to study treatment, SAEs, TEAEs leading to death, and TEAEs leading to discontinuation of the study will be tabulated for each treatment group.

Summary statistics, including the change from baseline, for vital signs, laboratory parameters, ECG intervals, and infusion-site reactions, will be provided by treatment, dose, visit, and time point, as appropriate.

8.8 Missing Data

Data from subjects who withdraw from the study, including AEs and any follow-up, will be included in analyzing primary, secondary, and exploratory outcomes.

Missing data (including those due to early discontinuations) will be not be imputed.

8.9 Interim Analyses

No formal interim analysis will be planned in this study.

9 REFERENCES

1. Knipe DM, Howley PM. Fields Virology. sixth edition ed. Two Commerce Square, 2001 Market Street, Philadelphia, PA 19103 USA: LIPPINCOTT WILLIAMS & WILKINS; 2013.
2. Peiris JS, Chu CM, Cheng VC, et al. Clinical progression and viral load in a community outbreak of coronavirus-associated SARS pneumonia: a prospective study. *Lancet* 2003; **361**(9371): 1767–72.
3. Memish ZA, Perlman S, Van Kerkhove MD, Zumla A. Middle East respiratory syndrome. *Lancet* 2020.
4. Huang C, Wang Y, Li X, et al. Clinical features of patients infected with 2019 novel coronavirus in Wuhan, China. *Lancet* 2020.
5. Lu R, Zhao X, Li J, et al. Genomic characterisation and epidemiology of 2019 novel coronavirus: implications for virus origins and receptor binding. *Lancet* 2020.
6. Zhu N, Zhang D, Wang W, et al. A Novel Coronavirus from Patients with Pneumonia in China, 2019. *N Engl J Med* 2020.
7. Xu Z, Shi L, Wang Y, et al. Pathological findings of COVID-19 associated with acute respiratory distress syndrome. *Lancet Respir Med* 2020.
8. Novel Coronavirus Pneumonia Emergency Response Epidemiology T. [The epidemiological characteristics of an outbreak of 2019 novel coronavirus diseases (COVID-19) in China]. *Zhonghua Liu Xing Bing Xue Za Zhi* 2020; **41**(2): 145–51.
9. https://www.who.int/emergencies/diseases/novel-coronavirus-2019

10 APPENDICES

Appendix 1 Abbreviations

Abbreviation	Definition
ADA	Anti-drug antibody
AE	Adverse event
ALT	Alanine aminotransferase
AST	Aspartate aminotransferase
$AUC_{(0\text{-}inf)}$	Area under the concentration-time curve from time zero extrapolated to infinity, calculated by linear up/log down trapezoidal summation
$AUC_{(0\text{-}last)}$	Area under the concentration-time curve from time zero to the time of the last quantifiable concentration, calculated by linear up/log down trapezoidal summation
BMI	Body mass index
CAPA	Corrective and preventative action
C_{max}	Maximum concentration, obtained directly from the observed concentration-versus-time data
CMI	Consumer medicine information
CRO	Contract research organization
CSR	Clinical study report
CTCAE	Common Terminology Criteria for Adverse Events
DBIL	Direct bilirubin
DNA	Deoxyribonucleic acid
EC_{50}	50% effective concentration
EC_{90}	90% effective concentration
ECG	Electrocardiogram
eCRF	Electronic case report form
ELISA	Enzyme-linked immunosorbent assay
EOS	End of study

Abbreviation	Definition
ET	Early termination
FDA	Food and Drug Administration
FIH	First-in-human
GLP	Good Laboratory Practice
HREC	Human research ethics committee
HRT	Hormone replacement therapy
IB	Investigator’s brochure
ICF	Informed consent form
ICH	International Council for Harmonisation
IgG2	Immunoglobulin G2
IMP	Investigational medicinal product
INR	International normalized ratio
LDH	Lactate dehydrogenase
LFT	Liver function test
MCB	Master cell bank
MedDRA	Medical Dictionary for Regulatory Activities
MIC	Minimum inhibitory concentration
MPC	Minimum parasiticidal concentration
NOAEL	No observed adverse effect level
NTF	Note to file
PCR	Polymerase chain reaction
PD	Pharmacodynamic(s)
PK	Pharmacokinetic(s)
QTcB	Bazett’s QT correction formula
QTcF	Fridericia’s QT correction formula
RO%	Receptor occupancy rate
SAE	Serious adverse event
SAP	Statistical analysis plan

**Abbreviation**	**Definition**
SOP	Standard operating procedures
SRC	Safety Review Committee
SUSAR	Suspected unexpected serious adverse reaction
$t_{½}$	Terminal half-life
TBIL	Total bilirubin
TEAE	Treatment-emergent adverse event
WOCBP	Woman of childbearing potential

Appendix 2 Regulatory, Ethical, and Study Oversight Considerations

Regulatory and Ethical Considerations

- This study will be conducted in accordance with the protocol and with the following:
  - Consensus ethical principles derived from international guidelines including the Declaration of Helsinki and Council for International Organizations of Medical Sciences International Ethical Guidelines.
  - Applicable International Council on Harmonisation (ICH) Good Clinical Practice (GCP) Guidelines.
  - Therapeutic Goods Administration (2000) (NHMRC National Statement on Ethical Conduct in Human Research – 2007, updated 2018).
  - Applicable laws and regulations.
- The protocol, protocol amendments, Informed Consent Form (ICF), Investigator Brochure, and other relevant documents (e.g., advertisements) must be submitted to the Human Research Ethics Committee (HREC) by the Investigator and reviewed and approved by the HREC before the study is initiated.
- Any amendments to the protocol will require HREC and regulatory authority approval, when applicable, before the implementation of changes made to the study design, except for changes necessary to eliminate an immediate hazard to subjects.
- The Investigator will be responsible for the following:
  - Providing written summaries of the status of the study to the HREC annually or more frequently in accordance with the requirements, policies, and procedures established by the HREC.
  - Notifying the HREC of serious adverse events (SAEs) or other significant safety findings as required by HREC procedures.
  - Providing oversight of the conduct of the study at the study center and adherence to requirements of the Therapeutics Goods Administration, ICH guidelines, the HREC, and all other applicable local regulations.

- After reading the protocol, each Investigator will sign the protocol signature page and send a copy of the signed page to the Sponsor or representative. The study will not start at any study center at which the Investigator has not signed the protocol.

Adequate Resources

The Investigator is responsible for supervising any individual or party to whom the Investigator delegates study-related duties and functions conducted at the study center.

If the Investigator/institution retains the services of any individual or party to perform study-related duties and functions, the Investigator/institution should ensure this individual or party is qualified to perform those study-related duties and functions and should implement procedures to ensure the integrity of the study-related duties and functions performed and any data generated.

Financial Disclosure

Investigators and sub-Investigators will provide the Sponsor with sufficient, accurate financial information as requested to allow the Sponsor to submit a complete and accurate financial certification or disclosure statements to the appropriate Regulatory Authorities. Investigators are responsible for providing information on financial interests during the course of the study and for 1 year after completion of the study.

Insurance

The Sponsor will ensure sufficient insurance is available to enable it to indemnify and hold the Investigator(s) and relevant staff as well as any hospital, institution, ethics committee, or the like, harmless from any claims for damages for unexpected injuries, including death, that may be caused by the subject's participation in the study but only to the extent that the claim is not caused by the fault or negligence of the subject(s) or Investigator(s).

Informed Consent Process

- The Participant Information Sheet and ICF describe in detail the study interventions, procedures, and risks.
- The Investigator or their representative will explain the nature of the study to the subject and answer all questions regarding the study.

- Subjects must be informed that their participation is voluntary. Subjects will be required to sign a statement of informed consent that meets the requirements of the Therapeutic Goods Administration, ICH guidelines, all other applicable local laws, and the HREC or study center.
- The medical record must include a statement that written informed consent was obtained before the subject was entered into the study and the date the written consent was obtained. The authorized person obtaining the informed consent must also sign the ICF.
- Subjects must be re-consented to the most current version of the ICF(s) during their participation in the study.
- A copy of the ICF(s) must be provided to the subject or the subject's legally authorized representative.
- For Parts B and C, subjects will also receive an Informed Consent for Blood Storage and an option to grant permission to be contacted about future study involvement.
- A subject who is rescreened is not required to sign another ICF if the rescreening occurs within 28 days from the previous ICF signature date.

The ICF will contain a separate section that addresses the use of remaining mandatory samples for optional exploratory research. The Investigator or authorized designee will explain to each subject the objectives of the exploratory study. Subjects will be told that they are free to refuse to participate and may withdraw their consent at any time and for any reason during the storage period. A separate signature will be required to document a subject's agreement to allow any remaining specimens to be used for exploratory research. Subjects who decline to participate in this optional research will not provide this separate signature.

Data Protection

- Subjects will be assigned a unique identifier by the Sponsor. Any subject records or datasets that are transferred to the Sponsor will contain the identifier only; subject names or any information which would make the subject identifiable will not be transferred.
- The subject must be informed that their personal study-related data will be used by the Sponsor in accordance with local data protection law. Subjects will be informed that their data will be held on file by the clinical study site and that the data may be viewed by the staff of the clinical study site (including, where necessary, clinical

study site staff other than the Investigator). The level of disclosure must also be explained to the subject.

- Upon request, the Investigator/institution(s) will permit direct access to source data and documents for study-related monitoring, audits, ethics committee review, and regulatory inspection(s) by the Sponsor (or their appropriately qualified delegates) and Regulatory Authorities. The subject must be informed that their medical records may be examined by Clinical Quality Assurance auditors or other authorized personnel appointed by the Sponsor, by appropriate HREC members, and by inspectors from Regulatory Authorities.
- Subjects will also be informed that a report of the study will be submitted to the Sponsor and may also be submitted to government agencies and perhaps for publication, but that they will only be identified in such reports by their study identification number, initials, and possibly their gender and age. The Investigators undertake to hold all personal information in confidence.
- Subjects will be informed that samples collected for the purposes described in the protocol will be sent to the Sponsor's nominated national or international laboratory for assessment.

Dissemination of Clinical Study Data

After completion of the study, a Clinical Study Report (CSR) will be written by the Sponsors/designees in consultation with the Investigator following the guidance in ICH E3.

Data Quality Assurance

- All subject data relating to the study will be recorded on printed or electronic case report forms (eCRFs) unless transmitted to the Sponsor or designee electronically (e.g., laboratory data). The Investigator is responsible for verifying that data entries are accurate and correct by physically or electronically signing the eCRF.
- The Investigator must maintain accurate documentation (source data) that supports the information entered in the eCRF.
- The Investigator must permit study-related monitoring, audits, HREC review, and regulatory agency inspections and provide direct access to source data documents. The Sponsor will require full verification for the presence of informed consent, adherence to the eligibility criteria, documentation of SAEs, and the recording of

data that was used for all primary and safety variables. Additional checks of the consistency of the source data with the eCRFs will be performed according to the study-specific monitoring plan. No information in source documents about the identity of the subjects will be disclosed.

- The CRO is responsible for the data management of this study, including quality checking of the data.
- Study monitors will perform ongoing source data verification to confirm that data entered into the eCRF by authorized study center personnel are accurate, complete, and verifiable from source documents; that the safety and rights of subjects are being protected; and that the study is being conducted in accordance with the currently approved protocol and any other study agreements, ICH GCP, and all applicable regulatory requirements. Key study personnel is required to be available to assist the field monitor during these visits.
- The study will be monitored according to the SOPs of the monitoring CRO appointed this task by the Sponsor, and all protocol deviations will be reported to the Sponsor. Serious breaches that impact subject safety or data integrity will also be reported to the Therapeutic Goods Administration and QIMR Berghofer HREC.
- Records and documents, including signed ICFs, pertaining to the conduct of this study must be retained by the Investigator for 15 years after study completion unless local regulations or institutional policies require a longer retention period. No records may be destroyed during the retention period without the written approval of the Sponsor. No records may be transferred to another location or party without written notification to the Sponsor.

Source Documents

The Investigator/institution should maintain adequate and accurate source documents and study records that include all pertinent observations on each of the study center’s subjects. Source data should be attributable, legible, contemporaneous, original, accurate, and complete. Changes to source data should be traceable, should not obscure the original entry, and should be explained if necessary (e.g., via an audit trail).

- Source documents provide evidence for the existence of the subject and substantiate the integrity of the data collected. Source documents are filed at the Investigator’s study center.

- Data reported entered in the eCRF that are transcribed from source documents must be consistent with the source documents, or the discrepancies must be explained. The Investigator may need to request previous medical records or transfer records, depending on the study. Also, current medical records must be available.

Protocol Deviations

Protocol Deviation: a protocol deviation is any departure, change, and/or addition from the study design or procedures defined in the approved protocol.

Suspected serious breach: a report that is judged by the reporter as a possible serious breach of GCP but has yet to be formally confirmed as a serious breach by the Sponsor.

Serious breach: A breach of GCP or the protocol that is likely to affect to a significant degree: a) the safety or rights of a study subject, or b) the reliability and robustness of the data generated in the clinical study.

Note to File (NTF): a note to file is a record that documents in detail actions taken, important decisions made, or explains a sequence of events where no other detailed record exists to enable the conduct of the study to be reconstructed. Note – an NTF is an unacceptable way to recode deviations during the study.

Corrective and Preventative Action Plans (CAPA): a CAPA plan will be developed if requested by the Sponsor, HREC, or other authorized parties as outlined in the Sponsor’s SOP.

Reporting requirements:

- All protocol deviations will be documented in the source documents and included in the CSR.
- All NTFs, protocol deviations, suspected serious breaches, and serious breaches are to be viewed by the Investigator’s delegate and signed by the Investigator.
- All NTFs, protocol deviations, suspected serious breaches, and serious breaches will be assessed and assigned significance at the end of each cohort by the study team.
- All suspected serious breaches are to be reported by the clinical study site to the Sponsor within 72 hours. If the Sponsor identifies a serious breach, assessment should be made and include:

  - If the incident is isolated or persistent.
  - Impact on safety and data.
  - Assessment of cause.
  - Assessment on reporting requirements.
  - If a CAPA plan is required.

- Serious Breaches should be reported by the Sponsor to the clinical study site and HREC within 7 days as well as the TGA and other relevant Regulatory Authorities if the breach has led to a clinical study site closure or involve a defective product that has a wider supply implication.
- All protocol deviations will be reported by the clinical study site to the Investigator as early as possible but within 7 days, and to the Sponsor at the end of each cohort.
- Protocol deviation logs will be submitted by the clinical study site to the Sponsor and QIMR Berghofer HREC via inclusion with the annual report.

Study and Study Center Closure

The Sponsor, Principal Investigator, FMD, and Regulatory Authorities independently reserve the right to discontinue the study at any time for safety or other reasons. This will be done in consultation with the Sponsor where practical.

Study centers will be closed upon study completion. A study center is considered closed when all required documents and study supplies have been collected and a study center closure visit has been performed.

The Investigator may initiate study center closure at any time, provided there is reasonable cause and sufficient notice is given in advance of the intended termination.

Reasons for the early closure of a study center by the Sponsor or Investigator may include but are not limited to:

- Failure of the Investigator to comply with the protocol, the requirements of the HREC or local health authorities, the Sponsor's procedures, or GCP guidelines.
- Inadequate recruitment of subjects by the Investigator.
- Discontinuation of further study treatment development.

In the event of premature study termination or suspension, the above-mentioned parties will be notified in writing by the terminator/suspender stating the reasons for early termination or suspension (with the exception of the Sponsor's responsibility for

notifying the Regulatory Authorities). After such a decision, the Sponsor and the Investigator will ensure that adequate consideration is given to the protection of the subjects' interest and safety. The Investigator must review all subjects as soon as practical and complete all required records.

Publication Policy

The data management, statistical, and medical writing team appointed by the Sponsor will collaborate to provide a detailed CSR upon conclusion of the study. This will include appendices of all tables and listings generated during the analyses of data. The tables, figures, and listings will be provided by the Sponsor. The Sponsor undertakes to ensure that all safety observations made during the conduct of the study are documented in this report.

Publication and reporting of results and outcomes of this study will be accurate and honest, and undertaken with integrity and transparency. The Sponsor recognizes that the Principal Investigator have a responsibility to ensure that results of scientific interest arising from the study are appropriately published and disseminated. Publication of results will be subjected to fair peer-review. Authorship will be given to all persons providing significant input into the conception, design, and execution or reporting of the research. No person who is an author, consistent with this definition, will be excluded as an author without their permission in writing. Authorship will be discussed between researchers prior to study commencement (or as soon as possible thereafter) and reviewed whenever there are changes in participation. The acknowledgment will be given to collaborating institutions and hospitals, and other individuals and organizations providing finance or facilities.

In any press releases, publications or presentations, the financial contribution from the Sponsor to the study and its participation in the collaboration shall be expressly acknowledged. Data will not be released publicly until the manuscript is accepted for publication. In the case of no publication, the information will only be released to the public and media in accordance with the sponsor. However, the Investigator undertakes not to make any publication or release pertaining to the study and/or results of the study without the sponsor's prior written consent, being understood that the Sponsor will not unreasonably withhold its approval. The Sponsor has the right to publish the results of the study at any time.

The investigator shall not use the name of the Sponsor and/or of its employees in advertising or promotional material or publication without the prior written consent of the Sponsor. The Sponsor shall not use the name of the Investigator and/or the collaborators in advertising or promotional material or publication without having received their prior written consent(s).

The sponsor will ensure that the key design elements of this protocol are posted in a publicly accessible database such as Clinical Trials Registry or Clinicaltrials.gov. In addition, upon study completion and finalization of the study report, the results of this study will be either submitted for publication in an open-access journal and/or posted in a publicly accessible database of clinical study results.

Appendix 3 Adverse Events: Definitions and Procedures for Recording, Evaluating, Follow-up, and Reporting

Definition of Adverse Event

**AE Definition**
• An adverse event (AE) is any untoward medical occurrence in a subject, temporally associated with the use of study treatment, whether or not considered related to the study treatment.<br>• NOTE: An AE can therefore be any unfavorable and unintended sign (including an abnormal laboratory finding), symptom, or disease (new or exacerbated) temporally associated with the use of study treatment.

**Events Meeting the AE Definition**
• Any abnormal laboratory test results (hematology, biochemistry, or urinalysis) or other safety assessments (e.g., electrocardiogram, radiological scans, vital signs measurements), including those that worsen from baseline, considered clinically significant in the medical and scientific judgment of the Investigator (i.e., not related to the progression of underlying disease).<br>• Exacerbation of a chronic or intermittent pre-existing condition, including either an increase in frequency and/or intensity of the condition.<br>• New conditions detected or diagnosed after study treatment administration even though it may have been present before the start of the study.<br>• Signs, symptoms, or the clinical sequelae of a suspected drug-drug interaction.<br>• Signs, symptoms, or the clinical sequelae of a suspected overdose of either study treatment or concomitant medication. Overdose per se will not be reported as an AE/serious adverse event (SAE) unless it is an intentional overdose taken with possible suicidal/self-harming intent. Such overdoses should be reported regardless of sequelae.

**Events NOT Meeting the AE Definition**
• Medical or surgical procedure (e.g., endoscopy, appendectomy): the condition that leads to the procedure is the AE.<br>• Situations in which an untoward medical occurrence did not occur (social and/or convenience admission to a hospital).<br>• Anticipated day-to-day fluctuations of pre-existing disease(s) or condition(s) present or detected at the start of the study that does not worsen.

Definition of Treatment-Emergent Adverse Event

A treatment-emergent AE is an event that commences or a pre-existing event that worsens in severity, any time on or after initiation of investigational medicinal product (IMP) administration.

Definition of Serious Adverse Event

If an event is not an AE per the definition above, then it cannot be an SAE even if serious conditions are met (e.g., hospitalization for signs/symptoms of the disease under study, death due to progression of the disease).

**An SAE is defined as any untoward medical occurrence that, at any dose:**
**a) Results in death**
**b) Is life-threatening**<br>The term 'life-threatening' in the definition of "serious" refers to an event in which the subject was at risk of death at the time of the event. It does not refer to an event, which hypothetically might have caused death if it were more severe.
**c) Requires inpatient hospitalization or prolongation of existing hospitalization**<br>In general, hospitalization signifies that the subject has been detained (usually involving at least an overnight stay) at the hospital or emergency ward for observation and/or treatment that would not have been appropriate in the physician's office or outpatient setting. Complications that occur during hospitalization are AEs. If a complication prolongs hospitalization or fulfills any other serious criteria, the event is serious. When in doubt as to whether "hospitalization" occurred or was necessary, the AE should be considered serious.

Hospitalization for elective treatment of a pre-existing condition that did not worsen from baseline is not considered an AE.
**d) Results in persistent disability/incapacity**<br>• The term disability means a substantial disruption of a person's ability to conduct normal life functions.<br>• This definition is not intended to include experiences of relatively minor medical significance such as uncomplicated headache, nausea, vomiting, diarrhea, influenza, and accidental trauma (e.g., sprained ankle), which may interfere with or prevent everyday life functions but do not constitute a substantial disruption.
**e) Is a congenital anomaly/birth defect**
**f) Other situations:**<br>• Medical or scientific judgment should be exercised in deciding whether SAE reporting is appropriate in other situations such as important medical events that may not be immediately life-threatening or result in death or hospitalization but may jeopardize the subject or may require medical or surgical intervention to prevent 1 of the other outcomes listed in the above definition. These events should usually be considered serious.<br>Examples of such events include invasive or malignant cancers, intensive treatment in an emergency room or at home for allergic bronchospasm, blood dyscrasias or convulsions that do not result in hospitalization, or the development of drug dependency or drug abuse.

Expectedness of an AE or SAE

**Unexpected**
• An AE is regarded as an unexpected event if its nature or severity is not consistent with the applicable reference safety information (Investigator Brochure's [IBs] or approved manufacturer's prescribing information for marketed drugs).<br>• Events that add significant information on the specificity, severity, or frequency of previously described reactions are also regarded as unexpected.<br>• A suspected unexpected serious adverse reaction is any SAE where a causal relationship with a study intervention is at least a reasonable possibility. The event is not listed in the IBs or product information.
**Expected**
• Expected AEs from meplazumab dosing are listed in and the meplazumab IB.

Recording and Follow-up of AE and/or SAE

**AE and SAE Recording**
• When an AE/SAE occurs, it is the responsibility of the Investigator to review all documentation (e.g., hospital progress notes, laboratory reports, and diagnostics reports) related to the event.<br>• The Investigator will then record all relevant AE/SAE information in the electronic case report form (eCRF). Each event must be recorded separately with the following information:<br>○ Description of the event.<br>○ Dates and times of onset and resolution.<br>○ Duration in hours.<br>○ Seriousness (SAE or not).<br>○ Severity.<br>▪ Only one AE will be reported if an AE changes in severity over time (the maximum severity will be recorded in the eCRF).<br>▪ Changes in the severity of an AE will be documented in the source documents to allow assessment of the duration of the event at each level of severity.<br>▪ AEs characterized as intermittently require documentation of onset and duration at each episode.

- If the AE resolves and then reoccurs at a later date, then 2 AEs are reported.

  - The action was taken in response to the AE (including treatment required)
  - Outcome (recovered/resolved, recovered/resolved with sequelae, not recovered/nor resolved, fatal, unknown).

- It is **not** acceptable for the Investigator to send photocopies of the subject's medical records in lieu of completion of the AE/SAE eCRF page.
- There may be instances when copies of medical records for certain cases are requested by the Sponsor. In this case, all subject identifiers, with the exception of the subject number, will be redacted on the copies of the medical records before submission to the Sponsor.
- The Investigator will attempt to establish a diagnosis of the event based on signs, symptoms, and/or other clinical information. Whenever possible, the diagnosis (not the individual signs/symptoms) will be documented as the AE/SAE.

Severity of Event

In addition to determining whether an AE fulfills the criteria for an SAE or not, the severity of AEs experienced by subjects will be recorded in accordance with the Common Terminology Criteria for Adverse Events Version 5.0, published 27 November 2017.

The severity of AEs will be graded as follows:

- Grade 1: Mild; asymptomatic or mild symptoms; clinical or diagnostic observations only; intervention not indicated.
- Grade 2: Moderate; minimal, local or noninvasive intervention indicated; limiting age-appropriate instrumental activities of daily living.
- Grade 3: Severe or medically significant but not immediately life-threatening; hospitalization or prolongation of hospitalization indicated; disabling; limiting self-care activities of daily living.
- Grade 4: Life-threatening consequences; urgent intervention indicated.
- Grade 5: Death related to AE.

A mild, moderate, or severe AE may or may not be serious. These terms are used to describe the intensity of a specific event. Medical judgment should be used on a case-by-case basis.

Seriousness, rather than severity assessment, determine the regulatory reporting obligations.

**Assessment of Causality**
• The Investigator is obligated to assess the relationship between study treatment and each occurrence of each AE/SAE. The AE/SAE must be characterized as **related/suspected** and **not related/not suspected**.<br>○ "Related/Suspected": The temporal relationship between the event and the administration of the study interventions is compelling and/or follows a known or suspected response pattern to that study intervention, and the event cannot be explained by the subject's medical condition, other therapies or accident.<br>○ "Not related/Not suspected": The event can be readily explained by other factors such as the subject's underlying medical condition, concomitant therapy, or accident and no plausible temporal or biologic relationship exists between any of the study interventions and the event.<br>• Alternative causes, such as underlying disease(s), concomitant therapy, and other risk factors, as well as the temporal relationship of the event to study treatment administration, will be considered and investigated.<br>• The Investigator will also consult the IB and/or Product Information, for marketed products, in their assessment.<br>• For each AE/SAE, the Investigator must document in the medical notes that they have reviewed the AE/SAE and has provided an assessment of causality.<br>• There may be situations in which an SAE has occurred, and the Investigator has minimal information to include in the initial report to the Sponsor. However, it is very important that the Investigator always make an assessment of causality for every event before the initial transmission of the SAE data to the Sponsor.<br>• The Investigator may change their opinion of causality in light of follow-up information and send an SAE follow-up report with the updated causality assessment.<br>• The causality assessment is one of the criteria used when determining regulatory reporting requirements.

**Follow-up of AEs, and SAEs**
• The Investigator is obligated to perform or arrange for the conduct of additional measurements and/or evaluations as medically indicated or as requested by the Sponsor to elucidate the nature and/or causality of the AE or SAE as fully as possible. This may include additional laboratory

tests or investigations, histopathological examinations, or consultation with other health care professionals.<br>• New or updated information will be recorded in the completed initially eCRF.<br>• The Investigator will submit any updated SAE data to the Sponsor within 24 hours of receipt of the information.

Reporting of SAEs

**SAE Reporting via an Electronic Data Collection Tool**
• The primary mechanism for reporting an SAE will be the electronic data collection tool.<br>• If the electronic system is unavailable for more than 24 hours, then the study center will use the paper SAE data collection tool (see next section).<br>• The study center will enter the SAE data into the electronic system as soon as it becomes available.<br>• After the study is completed at a given study center, the electronic data collection tool will be taken off-line to prevent the entry of new data or changes to existing data.<br>• If a study center receives a report of a new SAE from a subject or receives updated data on a previously reported SAE after the electronic data collection tool has been taken off-line, then the study center can report this information on a paper SAE Form (see next section) or by telephone.

**SAE Reporting via Paper CRF**
• Facsimile transmission of the SAE paper CRF is the preferred method to transmit this information.<br>• In rare circumstances and in the absence of facsimile equipment, notification by telephone is acceptable with a copy of the SAE data collection tool sent by overnight mail or courier service.<br>• Initial notification via telephone does not replace the need for the Investigator to complete and sign the SAE CRF pages within the designated reporting time frames.

Signature of Investigator

PROTOCOL TITLE: A Single-Center Clinical Trial in Healthy Volunteer to Evaluate Biodistribution of 131I-labelled Meplazumab

PROTOCOL NO: MPZ-I-03

VERSION: Original Protocol

This protocol is a confidential communication of [Sponsor]. I confirm that I have read this protocol, I understand it, and I will work according to this protocol. I will also work consistently with the ethical principles that have their origin in the Declaration of Helsinki and that are consistent with Good Clinical Practice and the applicable laws and regulations. Acceptance of this document constitutes my agreement that no unpublished information contained herein will be published or disclosed without prior written approval from the Sponsor.

Instructions to the Investigator: Please SIGN and DATE this signature page. PRINT your name, title, and the name of the study center in which the study will be conducted. Return the signed copy to the Sponsor and CRO.

I have read this protocol in its entirety and agree to conduct the study accordingly:

Signature of Investigator: ____________________________ Date: ________

Printed Name: ____________________________

Investigator Title: ____________________________

Name/Address of Center: ____________________________

____

____
