# Supplementary material for: Safety and efficacy of meplazumab in healthy volunteers and COVID-19 patients: a randomized phase 1 and an exploratory phase 2 trial
Source: Signal Transduct Target Ther. 2021 May 17;6:194. doi: 10.1038/s41392-021-00603-6 (PMC8127508; doi:10.1038/s41392-021-00603-6)
Supplement: Supplementary file 4 — Supplementary protocol 3 [file 41392_2021_603_MOESM4_ESM.pdf]

Humanized Meplazumab for Injection in the Treatment of Patient with COVID-19 Pneumonia: A Single-Center, Single-Arm, Open-labelled Add on Clinical Trial

**Study leader:**	Zhi-Nan Chen, Dr./Prof./Academician
**Chief investigators:**	Jian-Qi Lian, Dr./Prof./ Chief Physician
**Sponsor:**	Fourth Military Medical University
**Monitors:**	Huijie Bian, Dr./Prof.
**Trial site:**	Center for Infectious Diseases, Tangdu Hospital, Fourth Military Medical University
**Technical departments:**	National Translational Science Center for Molecular Medicine & Department of Cell Biology, Fourth Military Medical University<br>Department of Clinical Immunology, Xijing Hospital, Fourth Military Medical University<br>Department of Clinical Diagnosis, Tangdu Hospital, Fourth Military Medical University
**Version number:**	Version 1.2
**Draft date:**	February 1, 2020
**Final date:**	February 3, 2020

Contents

1. Introduction 7
1.1 SARS-CoV-2 7
1.2 CD147 7
1.3 Humanized Meplazumab Injection 9
1.4 Therapeutic mechanisms of Meplazumab for COVID-19 9
2. Summary of preclinical findings 10
2.1 Interaction between CD147 and Coronavirus 10
2.2 Non-clinical toxicological study of humanized meplazumab injection 12
3. Research purpose 13
4. Study the design and principles 13
4.1 Overall design 13
4.2 overview of research design 13
4.3 Study design principles 13
5. Subject selection 14
5.1 Inclusion criteria 14
5.2 Exclusion criteria 14
6. Allocation and blinding method 15
7. Dosage and administration 15
7.1 Dose escalation 15
7.2 Treatment scheme 16
8. Concomitant therapy 18
9. Concomitant medication 18
9.1 Medications allowed during the study period 18
9.2 Drug therapy that subjects were taboo to use during the study 18
10. Research and evaluation 19
10.1 Evaluation indexes of antiviral efficacy 19
10.2 Therapeutic evaluation 19
10.3 Safety evaluation 19
11. Termination of the study 20
12. Completion and exit of subjects 20
12.1 Completion 20
12.2 Termination and exit 20
12.3 Case report form (CRF) 22
12.4 Record retention 22
13. Statistical analysis 22
13.1 Statistics and analysis scheme 22
13.2 Analysis data set 22
13.3 General principles of statistical analysis 23

13.4 Curative effect analysis 23
13.5 Safety analysis 23
13.6 Interim analysis 24
13.7 Determine the sample size 24
14 Adverse event report 24
14.1 Related definition 24
14.2 Other points to consider for PTE and AE 26
14.3 Definition of causation 29
14.4 Criteria for judging severity 30
14.5 The measure related to the investigational product 30
14.6 Outcome of AE 31
14.7 Special report 31
14.8 Collection and reporting procedures 32
14.9 Death 34
14.10 Hospitalized 34
14.11 Pregnancy 35
14.12 Overdose 35
15 Research Drug Information 35
15.1 The name and specifications of the treatment 35
15.2 Formulation 37
15.3 Drug management 38
16. Ethics 38
16.1 Responsibility of the investigator 38
16.2 Independent ethics cast (IEC) /institutional review board (IRB) 38
16.3 Informed consent 39
16.4 Protection of subject data 39
16.5 Data monitoring committee (DMC) 39
17. Management requirements 40
17.1 Modify the program 40
17.2 Data management 40
17.3 Inspection 41
17.4 Audit and Inspection 41
17.5 Original Records for Verification 42
17.6 The End/Termination of the Research 42
17.7 Confidentiality Agreement and Patient’s Privacy 43
17.8 The Use and Publication of the Information 43
18. References 44

Schedule of Assessments

	Screening	Baseline[1]	Treatment Period						Short Follow-Up Period											
Visit Point	-3~-2d	-1d	0d	1d	2d	3d	4d	5d	6d	7d	8d	9d	10d	11d	12d	13d	20d	27d
Visit No.	V1	V2	V3	V4	V5	V6	V7	V8	V9	V10	V11	V12	V13	V14	V15	V16	V17	V18
Informed Consent	X																	
Inclusion/Exclusion Criteria	X																	
Demography [2]	X																	
Medical/Medication History	X																	
Vital signs [3]	X	X	X	X	X	X	X	X	X	X	X	X	X	X	X	X	X	X
Physical examination	X	X	X	X	X				X			X				X	X	X
Blood routine [4]	X	X	X	X		X		X		X		X		X		X	X	X
Blood biochemistry [5]	X	X	X	X		X		X		X		X		X		X	X	X
Blood coagulation function [6]		X				X				X								
Partial arterial oxygen pressure		X			X				X			X				X	X	X
Urine routine [7]		X	X	X					X							X	X	X
SARS-CoV-2 nucleic acid detection [8]		X	X	X		X		X		X		X		X		X	X	X
12-lead ECG [9]	X	X	X	X						X								X
Chest CT/Chest X-ray	X	X							X							X	X	X
CRP assay[10]		X	X	X		X		X		X		X		X		X	X	X
Cytokine assay [10]		X				X		X				X				X	X	X
Cell subgroup [11]		X				X		X				X				X	X	X
meplazumab infusion [12]			X	X		X												
Drug combination	X																	
Adverse reactions	X																	

Notes:

1. If the screening date is continuous with the baseline date, only the baseline examination will be performed.
2. Demography: including gender, date of birth, height, weight.
3. Vital signs: including blood pressure (systolic and diastolic), pulse rate and O_2 saturation (finger oxygen SpO_2), respiration rate, and body temperature. The examination should be performed at 30 min before administration and 1h ± 10 min post the administration; then it should be performed every 6 h within 24 h post-administration, and every 1 d till the end of short-term follow-up.
4. Blood routine: including white blood cell (WBC), red blood cell (RBC), hemoglobin (HGB), platelet (PLT), lymphocyte (LY), monocyte (MO), neutrophil (NE), eosinophil count (EO#), and basophil count (BA#). Samples were collected within 6 hours after each administration and every 2 days after the end of treatment until the end of the short-term follow-up period.
5. Blood biochemistry: including fasting blood glucose (FBG), creatinine (Cr), urea (BUN), uric acid (UA), aspartate transferase (AST) and alanine transferase (ALT) Gamma-glutamyltransferase (γ-GT), total bilirubin (TBIL), albumin (ALB), total protein (TP), sodium (NA), potassium (K), calcium (Ca), chloride (CL), lactate, and cholinesterase. Samples were collected within 6 hours after each administration and every 2 days after the end of treatment until the end of the short-term follow-up period.
6. Blood coagulation function: including prothrombin time (PT), activated partial thromboplastin time (APTT), international normalized ratio (INR), and plasma fibrinogen (FIB).
7. Urine routine: includes urine white blood cell, urine nitrite, urobilinogen, urine occult blood, urine pH value, urine specific gravity, urine bilirubin, urine ketone body, urine glucose and urine protein. The treatment period was checked 6 hours after each administration, and once a week thereafter until the end of the short-term follow-up period.
8. For 2019-nCoV nucleic acid detection, the drug was administered 12 hours after each administration in the treatment period, once every 2 days during the treatment period, and once a week thereafter until the end of short-term follow-up (28 days after the first treatment), or stop after the transition to negative.
9. 12-lead ECG: It should be measured after the treatment period (within 30 minutes), 1 week after the first dose, and 28 days after the first administration.
10. Cytokines (carried out when available), CRP detection: cytokines including interleukin-2, 6, 8, 10, 15 (IL-2, IL-6, IL-8, IL-10, IL-15), Interferon-gamma (IFN-γ), tumor necrosis factor alpha (TNF-α), and transforming growth factor-β1 (TGF-β1). Within 6hr after each administration. Test was performed every 2 days during the treatment period and weekly thereafter .
11. Immune cells (if applicable): test once every 2 days during the treatment period within 6 hours after each administration, and once every week thereafter.
12. Meplazumab was infused once on Day 0 and Day 1, respectively. The third infusion was decided based on the results of viral nucleic acid detection and clinical manifestations. The third reinfusion was performed 3-5 days after the second infusion.

1. Introduction

Coronavirus (CoV) is an RNA virus that can infect humans and a variety of vertebrates. It is also an important pathogen that causes the common cold and upper respiratory tract infections. However, in recent years, a variety of highly infectious and pathogenic CoV have appeared, including the severe acute respiratory syndrome coronavirus (SARS-CoV) outbreaking in 2002, the Middle East respiratory syndrome coronavirus (MERS-CoV) in 2012, and the novel coronavirus (SARS-CoV-2) at the end of 2019. The emergence of these coronaviruses is a serious health threat, leading to progressive respiratory failure or death. Currently, the treatment of SARS-CoV-2 is mainly to support symptomatic treatment and antiviral treatment, and no specific anti-virus drug has been approved. So, it is urgent to develop a new treatment method and drug for this viral infection. Based on the previous work, we carried out the clinical treatment of pneumonia caused by SARS-CoV-2. Using meplazumab, a humanized anti-CD147 monoclonal antibody, block the interaction between CD147 and Spike protein of SARS-CoV-2 to develop novel drug and new strategies for COVID-19 pneumonia.

1.1 SARS-CoV-2

Coronavirus is an enveloped, positive single-stranded RNA virus, which can infect humans and a variety of vertebrates. CoV is an important pathogen causing the common cold. To date, there are seven known human coronaviruses. Three of them have high infectivity and high mortality. They are SARS-CoV, MERS-CoV, and SARS-CoV-2. Typical symptoms of MERS include fever, cough, shortness of breath, and even pneumonia, with a case fatality rate of about 34.4%. Typical symptoms of SARS usually include fever, chills and body aches, and even pneumonia, with a case fatality rate of about 9.6%. CoV contains at least four structural proteins: spike protein (S protein), envelope protein (E protein), membrane protein (M protein), and nucleocapsid protein (N protein). The S protein promotes adhesion to the host and fusion with the cell membrane during virus infection, while the N protein is mainly involved in maintaining the stability of the genome and the composition of the nucleocapsid.

1.2 CD147

CD147 was first discovered by Biswas in 1982 and named the tumor cell collagenase stimulating factor. Then, it was successively called matrix metalloproteinase-inducing factor (EMMPRIN), M6, and HAb18G. At the 6th human leukocyte differentiation antigen collaboration group meeting, it has named CD147. CD147 belongs to the immunoglobulin superfamily member, is a type I transmembrane glycoprotein which is highly expressed in a variety of cancer cells, including liver cancer, glioblastoma, lung cancer, breast cancer, lymphoma, oral squamous cell carcinoma, melanoma, bladder cancer, and kidney cancer, etc., is a broad spectrum of tumor-associated antigen [1]. Meanwhile, CD147 expressed in host cells is an important receptor or coreceptor for various pathogens to invade target cells. CD147 has been reported to mediate human cytomegalovirus [2], HIV [3], measles virus [4], and other

infections in the host. Our previous study showed that CD147 plays a role in SARS-CoV infected host cells by identifying CyPA bound to the SARS-CoV N protein. Antagonistic peptide (AP)-9 of CD147 blocks viral infection [5]. Our recent results indicate that the RAP2 protein expressed by Plasmodium falciparum plays an important role in the process of invading host red blood cells through direct interaction with CD147 molecules on the erythrocyte membrane. By adding the humanized monoclonal antibody with CD147 target, the invasion of red blood cells by Plasmodium could be blocked, and the therapeutic and preventive effects of falciparum malaria could be realized [6].

In 2007, Licartin (iodine [$_{131}$I] -labeled metuximab injection), which is anti-CD147 Mab and developed by the National Translational Science Center for Molecular Medicine, was approved for the treatment of liver cancer. Licartin is a novel biological product, it is delivers $_{131}$I to the tumor site specifically by binding CD147 on the surface of cancer cells, thus producing the anti-tumor effect. It has reported that the tumor recurrence rates of one year and two years in patients, who received Radiofrequency Ablation (RFA) combined with Licartin, has decreased by 24.5% and 12.4%, respectively, compared with the control group treated with RFA alone, and the median time of tumor recurrence was extended by 7 months [7]. Other data showed that among 138 patients with HCC, the median survival was extended by 6 months in the Licartin combined with TACE group compared with the TACE group alone [8]. The above data indicate that the target-CD147 therapy is an effective method for the treatment of liver cancer. Multicenter phase IV clinical studies also showed that Licartin combined with TACE significantly improved 1-year survival compared to TACE alone (79.47% vs. 65.59%, HR=0.598, P=0.041) [9]. A randomized controlled study of anti-relapse treatment

after liver transplantation for hepatocellular carcinoma showed that compared with the control group, the 1-year recurrence rate was reduced by 30.4%, the survival rate was increased by 20.6%, and the AFP negative maintenance rate reached 87.82% [10]. In addition, the above clinical applications and studies have shown that the application of monoclonal antibodies against the CD147 target has good safety in the human body, and no adverse reactions related to monoclonal antibodies have been observed.

In 2013, the CD147 detection kit (immunohistochemical method) was certified as a third-class medical device by the state and marketed. In 2015, humanized metozumab injection targeting CD147 entered the clinical stage I in the treatment of lung cancer. In 2019, humanized meplazumab injection was granted by the FDA as an orphan drug for malaria.

1.3 Humanized Meplazumab Injection

Meplazumab Injection is a novel humanized monoclonal antibody, which developed by the National Translational Science Center for Molecular Medicine, and Jiangsu Pacific Meinuoke Biopharmaceutical Co. LTD. Meplazumab is a recombinant human IgG_2 antibody expressed by CHO cells. We use bioinformatics and recombinant technology to replace the FR sequence in the light and heavy chain variable regions of the antibody with the human FR sequence. More than 2/3 of the whole molecule is the human source, in which the variable region has the function of binding antigen, while the constant region has the function of antibody effect, immunogenicity, and species characteristics. The FC segment of the chimeric antibody can prolong the half-life of the antibody in serum and theoretically reduce the immunogen of the heterologous antibody. Compared with its parent non-humanized MAb, the equilibrium dissociation constant of humanized antibody did not change significantly, and the affinity constant (KD) was 1.7×10^{-10}M, which indicated that Meplazumab and its parent non-humanized MAb 6H8 (affinity constant KD = 4.48 × 10-10M) had a similar affinity, which ensured the binding ability of MAb to target molecules in vivo and in vitro. On January 17, 2020, Humanized Meplazumab Injection has been approved by the FDA to carry out a phase I clinical trial in Australia.

1.4 Therapeutic mechanisms of Meplazumab for COVID-19

Coronavirus has at least four structural proteins, including spike protein (S), envelop protein (E), matrix protein (M), and nucleocapsid protein (N). Among them, S protein mediates the invasion to the host cell via binding ACE2 on the surface of the cell. N protein is the main component of the nucleocapsid. Recently, we revealed that CD147 could also be bound with S protein as a functional entry receptor, and the SARS-CoV-2 infection was efficiently inhibited by meplazumab in a dose-dependent manner. Meanwhile, CD147 is a receptor for ligand CyPA, and its interaction with CD147 was key to the inflammation and chemotaxis. The anti-CD147 antibody could attenuate the chemotactic index of T cells induced by CyPA. Relevant contents have been applied for a number of domestic and

international patents [11-14].

Therefore, in this study, the COVID-19 pneumonia was treated with humanized meplazumab with the following mechanisms:

(1) Meplazumab inhibited the invasion of SARS-CoV-2 by blocking the binding between S protein and CD147 on the surface of the host cell membrane.

(2) CyPA is one of the major pro-inflammatory factors, which participates in the inflammatory response of tissues and organs throughout the body. Meplazumab inhibited the interaction between CD147 and CyPA, preventing host cells from releasing pro-inflammatory factors after viral infection.

(3) Meplazumab blocks the activity of matrix metalloproteinases 2 and 9 in host cells, inhibits tissue fibrosis, and thus improves the prognosis of patients.

Based on these shreds of evidence, meplazumab can be used as a new drug for the treatment of coronavirus infection, including SARS-CoV-2, with good clinical application prospects.

2. Summary of preclinical findings

2.1 Interaction between CD147 and Coronavirus

2.1.1 SARS-CoV S protein can specifically bind to CD147

Co-Immunoprecipitation (co-IP) analysis showed that SARS-CoV S protein expressed in vitro can specifically bind to CD147; SPR results as shown in Fig. 1 indicate that the K_D of

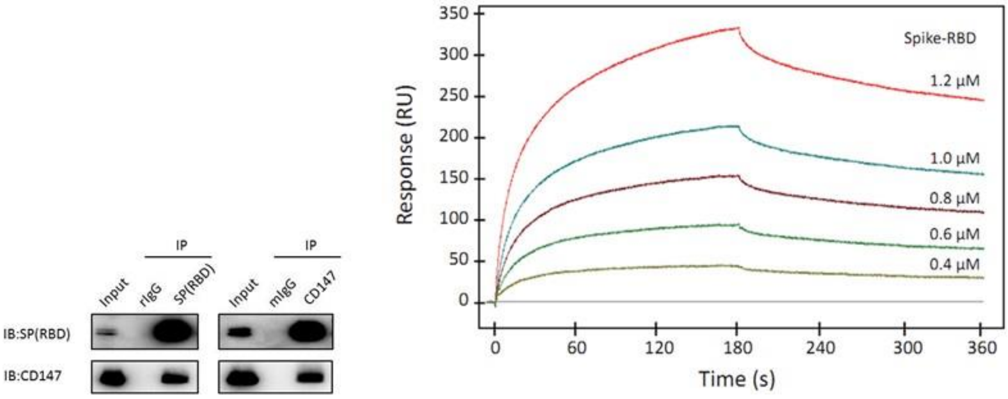

SARS-CoV spike protein binding to CD147 is 1.85×10^{-7}M.

Fig.1 Identification of interaction between CD147 and SP.

The interaction of CD147 and SP detected by co-IP assay (left) and SPR (right), $K_D=1.85\times10^{-7}$M.

2.1.2 Co-localization of CD147 and SP in Vero E6 cell.

Subcellular localization of SP and CD147 in SARS-CoV–infected Vero E6 cells were detected by immuno-electron microscope. Vero E6 cells infected with SARS-CoV-2 were observed by an immune-electron microscope. Using10 nm (CD147) and 20 nm (SP) gold colloid- labeled antibodies, we found that the two proteins, mainly presented in viral inclusion bodies of Vero E6 cells. These results reinforce the finding that the CD147-SP interaction enhances viral invasion of host cells.(Fig. 2)

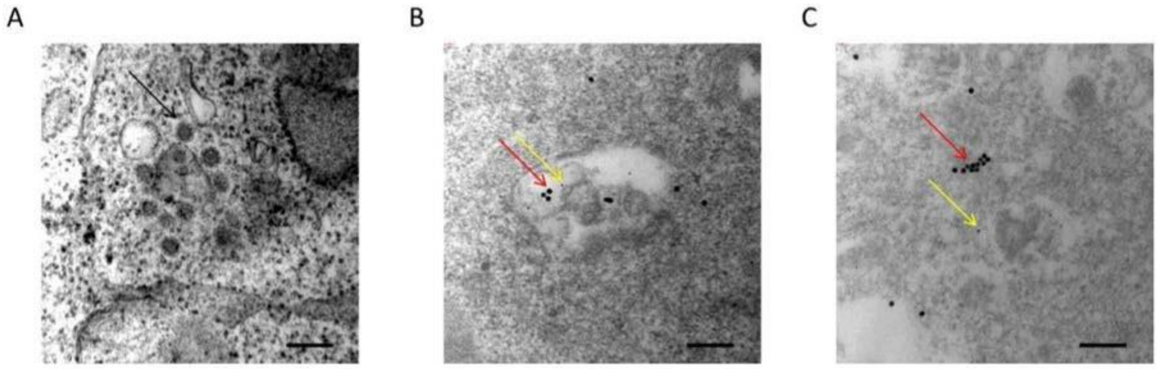

Fig. 2 Subcellular localization of CD147 (10nm) and SP (20nm) in SARS-CoV–2 infected Vero E6 cells

(A) SARS-CoV-2 virion in infected Vero E6 cell (arrow); (B and C) CD147 (10nm, yellow arrows) and SP (20nm, red arrows) in infected Vero E6 cell. Scale bar = 200nm.

2.2 Non-clinical toxicological study of humanized meplazumab injection

2.2.1 Pharmacological research

In a pharmacologic study using cynomolgus monkeys, 2, 6, and 12mg/kg of humanized meplazumab were administered intravenously once a week for 4 weeks, with a recovery period of 8 weeks, no significant effect on the cardiovascular system and respiratory rate.

In a pharmacology study using rhesus monkeys, 4, 20mg/kg of humanized meplazumab was administered intravenously once a week for 4 weeks, with a recovery period of 4 weeks, no significant effect on the cardiovascular system and respiratory rate.

2.2.2 Acute toxicity study

In the acute toxicity study using cynomolgus monkeys, neither male nor female animals in the 0, 30 and 100 mg/kg groups were found dead or dying. No abnormalities were observed in clinical observation, body weight, food intake, body temperature, electrocardiogram (ECG), coagulation, serum biochemistry and immune function, and gross anatomy. No animal death was observed at the maximum dose of 100 mg/kg, and no toxicity was observed with the test product.

2.2.3 Long-term toxicity study

Cynomolgus monkeys were administrated meplazumab with 2, 6, and 12 mg/kg/week for 4 times, with 8 weeks of recovery. There was no significant toxicity associated with the test product.

Rhesus monkeys were administrated meplazumab with 4 and 20mg/kg/ week for 4 times with 4 weeks of recovery. There was no significant toxicity observed in the 4mg/kg group.

2.2.4 Immunogenicity study

In cynomolgus monkeys, which received 0, 2, 6, and 12mg/kg treatment, the antibody was detected in 1/10, 8/10, 6/10, and 4/10 animals, respectively. The confirmation test indicated that positive individual animals were detected in all groups.

In rhesus monkeys, the antibody was detected in 4/10 and 1/10 animals in 4 and 20mg/kg doses groups, respectively. The confirmation test indicated that positive individual animals were detected in all the drug groups.

2.2.5 Local tolerance study

For vascular irritation studies, cynomolgus monkeys were administered intravenously with 2, 6, and 12mg/kg of meplazumab once a week for 4 weeks, with a recovery period of 8 weeks. At the end of the administration and the recovery period, the gross anatomic and histopathological examination of the injection sites (blood vessels and muscles) showed no administration-related stimulating effect was detected.

For vascular irritation studies, rhesus monkeys were administered intravenously with doses of 4 and 20mg/kg of meplazumab once a week for 4 weeks, with a recovery period of 4 weeks. At the end of the administration and the recovery period, the gross anatomic and histopathological examination of the administration sites (blood vessels and muscles) showed no administration-related stimulating effect was detected.

In vitro hemolysis studies showed that the dose of 2mg/mL humanized meplazumab did not cause hemolysis and coagulation in the peripheral erythrocyte suspension of human and cytophilic monkeys. The results of toxicological studies indicate that the humanized meplazumab injection has good safety, efficacy, and stable quality.

3. Research purpose

To assess the safety and superiority of humanized meplazumab in patients with novel coronavirus 2019 (COVID-19) pneumonia compares to recommended therapeutic.

4. Study the design and principles

4.1 Overall design

This is a single-center, single-arm, open-labeled add-on clinical trial.

4.2 Overview of research design

This is a prospective, single-center, single-arm, open-labeled add-on clinical trial. The aim of this trial is to evaluate the safety and superiority of meplazumab in patients with COVID-19 pneumonia who received the current recommended treatment.

The study plans to enroll 10-20 patients with COVID-19 pneumonia in Tangdu hospital of the Fourth Military Medical University.

4.3 Study design principles

According to the results of the preclinical study of humanized meplazumab, single

The dosage used for treatment. For eligible patients, 10mg meplazumab dissolved in 100mL saline will administrate at day 1 (d0) and day 2 (d1) by intravenous infusion, respectively. If necessary, the third administration will be performed between day 4 to day 6, which will be decided according to the following items:

1) SARS-CoV-2 nucleic acid assay.

2) Clinical manifestations.

3) The overall assessment of the doctor.

Patients will be assessed within 28 days post initial administration to determine the therapeutic effect and safety of meplazumab.

All patients should receive recommended treatment from *Diagnosis and Treatment for 2019 Novel Coronavirus Diseases* released by National Health Commission of China, and all necessary prevention/symptomatic treatment according to the clinical diagnosis are accepted. The end of the study was defined as the last visit of the last enrolled patient, or the last data point of statistical analysis, or the last safety visit of the last enrolled patient.

5. Subject selection

Patients who diagnosed as COVID-19 pneumonia according to the criteria of *Diagnosis and Treatment for 2019 Novel Coronavirus Diseases (Version 4)* released by the National Health Commission of China. Enrolled patients fulfilled inclusion and exclusion criteria

5.1 Inclusion criteria

Patients must meet **all** of the following criteria for inclusion:

- Man or woman aged 18 ≤ age ≤ 78.
- Patients with common, severe, or critical COVID-19 pneumonia were laboratory and clinically diagnosed according to *Diagnosis and Treatment for 2019 Novel Coronavirus Diseases* released by the National Health Commission of China.
- The subjects must understand the study and be willing to participate in the study, and sign the informed consent form.

5.2 Exclusion criteria

Patients who meet any of the following criteria are excluded from the study:

- Allergic reactions or a history of allergy to any of the ingredients treated in this trial.

- Patients not suitable to participate in this study by the judgment of the investigator. *Note: The investigator shall ensure that subjects meet all inclusion/exclusion criteria during screening. If the subject's status (including laboratory results) changes between the screening and the first administration and a certain exclusion criterion is met, the subject shall be excluded from the study.*

6. Allocation and blinding method

As a single dosage trial, patients should enroll in sequence. The second and third subjects start an infusion of meplazumab till 1 day after the treatment of the previous subject. The fourth and subsequent subjects are not required. As an open-labeled study, there is no need for blindness.

7. Dosage and administration

7.1 Dose escalation

This is a single dosage trial, and no dose escalation was involved.

The basis for dosage selection:

Based on the results of toxicological studies in non-human primates, inhibition assay for coronavirus infection *in vitro*, and pharmacological activity of humanized meplazumab, we choose 10mg as the clinical therapeutic dosage for each infusion, which is expected to take a low risk of adverse reactions.

Repeated dose toxicology studies conducted in cynomolgus monkeys and rhesus monkeys over a period of 4 weeks show that the lowest dose of No Observed Advanced Effect Level (NOAEL) of humanized meplazumab is 4 mg/kg. At this dose, the total $AUC_{(0\text{-}168h)}$ of monoclonal antibody in rhesus monkeys is 3420μg·h/mL (female) and 2894μg·h/mL (male), and the maximum blood concentration C_{max} is 80±9μg/mL (female) and 90±35μg/mL (male). In the repeated dose group of 1mg/kg, the total drug exposure $AUC_{(0\text{-}168h)}$ is 460±73.2 μg·h/mL, and the half-life is 47.8±11h. In a single-dose study, the exposure of 0.1mg/kg humanized meplazumab in rhesus monkeys is 126 ± 22.6h·μg/mL. The results of hemolysis and oxygen-carrying - oxygen-releasing test in vitro show that 2mg/mL of meplazumab can not cause hemolysis of human erythrocytes and has no significant effect on the oxygen-carrying and oxygen-releasing function of erythrocytes.

In vitro efficacy studies of coronavirus infection with HEK293 cells, IC_{50} of CD147 antibody is 31.25 μg/mL, and minimum inhibitory concentration (MIC) is 62.25 μg/mL.

The results of the clinical trials have shown that the single infusion of 10mg and 20mg of humanized meplazumab does not cause drug-related adverse reactions in subjects.

The end of study:

The end of the study is defined as the last visit of the last subject, the last data point for statistical analysis, or the lastsubject's safety visit.

7.2 Treatment scheme

This treatment scheme including the screening stage, pre-treatment preparation stage, treatment stage, and treatment end-stage. The treatment-related contents of each stage were as follows:

7.2.1 Screening stage

The patient who signed the informed consent will perform screening projects according to the study protocol. Only eligible patients would be allowed to proceed to the next stage.

7.2.2 Pre-treatment preparation stage

Subjects received a physical examination (including vital signs), SARS-CoV-2 nucleic acid test, blood routine test, examination of respiratory function, blood biochemistry test, urine routine test, and 12-lead ECG analysis one day before receiving humanized meplazumab injection treatment. Drugs that may be used in treatment were prepared at the same time.

7.2.3 Treatment stage

1) The first dose (0d)
   - Physical examination: before infusion.
   - Vital signs (including respiratory rate and oxyhemoglobin saturation): before infusion, 1hr±10 min post infusion, then every 6hr till 24 hour post infusion.
   - The intravenous drip of 30mL of methylprednisolone 30 mins before infusion.
   - 10mg of meplazumab was dissolved in 1mL sterile water, then diluted with 100mL 0.9% saline and administrated by intravenous infusion.
   - Blood routine test and blood biochemistry test: within 6hr post-infusion.

• ECG analysis: within 30mins post-infusion.

• Urine test: post-infusion.

• SARS-CoV-2 nucleic acid test: 12hr post-infusion.

• Blood sample of cytokines and C-reactive protein testing: within 6hr after dripping.

2) Second dose (1d)

• Physical examination: before infusion.

• Vital signs (including respiratory rate and oxyhemoglobin saturation): before infusion, 1hr±10 min post infusion, then every 6hr till 24 hour post infusion.

• Intravenous drip of 30mL of methylprednisolone 30 mins before infusion.

• 10mg of meplazumab was dissolved in 1mL sterile water, then diluted with 100mL 0.9% saline and administrated by intravenous infusion.

• Blood routine test and blood biochemistry test: within 6hr post-infusion.

• ECG analysis: within 30mins post-infusion.

• Urine test: post-infusion.

• SARS-CoV-2 nucleic acid test: 12hr post-infusion.

• Blood sample of cytokines and C-reactive protein testing: within 6hr after dripping.

3) Third dose (5d±1d) (if necessary)

Administer need to decide whether to perform the third dose based on the SARS-CoV-2 nucleic acid test results and clinical manifestations.

• Physical examination: before infusion.

• Vital signs (including respiratory rate and oxyhemoglobin saturation): before infusion, 1hr±10 min post-infusion, then every 6hr till 24-hour post-infusion.;

• The intravenous drip of 30mL of methylprednisolone 30 mins before infusion.

• 10mg of meplazumab was dissolved in 1mL sterile water, then diluted with 100mL 0.9% saline and administrated by intravenous infusion.

• Blood routine test and blood biochemistry test: within 6hr post-infusion.

• ECG analysis: within 30mins post-infusion.

• Urine test: post-infusion.

• The SARS-CoV-2 nucleic acid test: 12hr post-infusion.

• Blood sample of cytokines and C-reactive protein testing: within 6hr after dripping.

• Chest CT/ chest X-Ray: within 12hr post-infusion.

• The arterial partial pressure of oxygen (PaO_2): within 6hr post infusion.

7.2.4 Treatment end stage

The vital signs of the patient should be closely monitored during the treatment process and evaluated actively until the subjects indicators were stable. If the subject had a fatal allergic reaction, symptomatic treatments with oxygen inhalation, bronchodilators, epinephrine, antihistamines or glucocorticoids should be performed according to the first aid plan.

8. Concomitant therapy

Humanized meplazumab injections should be used under the supervision of the principal investigator or other researchers. Administration of all study drugs should be recorded in the CRF, as well as reasons for medication discontinuation, dose reduction, or omission. This information and drug counts for all study drugs per cycle would be used to assess treatment compliance.

9. Concomitant medication

All medicines, as well as obvious non-drug treatments (including physical therapy and blood or platelet transfusion) used in the 30 days before treatment until treatment finished, should be recorded at CRF in detail. Drugs included not only drugs prescribed by doctors but also all over-the-counter drugs, vitamins, and Chinese medicine soup drugs, proprietary Chinese medicines, etc.

9.1 Medications allowed during the study period

Researchers took the premise of protecting the interests and safety of the subjects, and subjects were still treated in accordance with the *Diagnosis and Treatment for 2019 Novel Coronavirus Diseases* released by the National Health Commission of China during the study period. The decision to use other concomitant drugs can be made without affecting the evaluation of the study drug. All the co-administration and symptomatic treatment during the trial should be recorded at CRF.

9.2 Drug therapy that subjects were taboo to use during the study

During the study, avoid the use of non-essential drug treatments that affect the evaluation of research drugs. If there were serious adverse reactions during the treatment, they should be dealt with according to the relevant clinical diagnosis and treatment guidelines.

10. Research and evaluation

10.1 Evaluation indexes of antiviral efficacy

Primary outcome:

Virological clearance (i.e., negative conservation rate and time to negative) using qRT-PCR in nasopharyngeal swab samples.

Secondary outcomes:

Time to recovery of vital sign (including body temperature, respiratory rate, and SPO_2);

Chest radiographic improvement;

Rate of PaO_2/FiO_2 recovery; Time

(days) to discharge;

Recovery of inflammation, including:

- Peripheral blood routine (white blood cell count, white blood cell classification, red blood cell, platelet (percentage and absolute value));
- Coagulation function;
- Blood biochemistry (liver and kidney function, creatine kinase, electrolyte, lactate dehydrogenase);
- Cytokines, CD3, CD4, CD8, B cells, NK cells.
- C-reactive protein.

10.2 Therapeutic evaluation

It was evaluated during the screening period, on the day of each administration, every day within one week after the last administration, and then once a week until 28 days post the first administration, or the subjects progressed, died, or fell off. If the subject withdraws in advance, try to perform an imaging examination to complete the evaluation of the curative effect.

10.3 Safety evaluation

During the trial, any adverse events (AE) /serious adverse events (SAE), occurred in all subjects during the study period were observed and recorded, including clinical symptoms, abnormal vital signs with clinical significance, abnormalities in laboratory examination, and abnormal ECG examination, to determine the correlation between them and research drugs, and to follow up the adverse events until they recover/return to normal, return to baseline, stable, or clinical judgment does not require follow-up, adverse events will be evaluated according to CTCAEv5.0.

After each administration, pay close attention to the changes of body temperature and other abnormalities and measure vital signs and blood oxygen saturation. During the test, routine blood routine, blood biochemistry, chest imaging examination, hematuria routine, finger oxygen saturation, arterial oxygen partial pressure, electrocardiogram and other indexes were detected.

11. Termination of the study

The sponsors have the right to terminate this study at any time. When the study is

terminated, the independent ethics committee (IEC) and the institutional review committee (IRB) must be reported. The reasons for termination of the study include, but are not limited to:

- Drug administration regulatory departments, ethics committees, sponsors or researchers believe that there are significant safety risks in therapeutic substances;
- The sponsor may terminate the study for any scientific, medical, or ethical reasons but must take full account of the rights, safety, and health of the subjects.
- Other reasons determined by the sponsor or researcher that it is not appropriate to continue the trial.

12. Completion and exit of subjects

12.1 Completion

If the subject completes all evaluations for 28 days after administration or dies in accordance with the study plan, the subject is regarded as a completed case.

12.2 Termination and exit

The withdrawal decided by the researcher

Withdrawal from the study refers to the fact that the selected subjects are not suitable to continue the study in the course of the study, and the researcher decides to withdraw from the study.

(1) In the clinical trial, the subjects have some complications or deterioration, so they are not suitable to continue the trial.
(2) The subjects disobeyed thedoctor’s advice and used other treatments without authorization, which affected the evaluation;
(3) The researchers believe that continuing treatment is disadvantageous to the patients.
(4) Subjects have adverse events or serious adverse events and are not suitable to continue to undergo the trial.
(5) The compliance of the subjects was poor, which affected the efficacy and safety.

The subjects dropped out of the study on their own.

The subjects are unwilling to continue to participate in the clinical study, and according to the provisions of the informed consent form, the subjects have the right to withdraw from the study at any stage of the study, or the subjects have not explicitly proposed to withdraw from the study, but no longer accept medication or testing and loss of follow-up is also a “withdrawal” (or “shedding”). The reasons for its withdrawal should be known and recorded as far as possible. Such as they find it difficult to tolerate some adverse reactions, can’t continue to accept clinical research for other reasons or lose follow-up without explaining the reasons, and so on.

Treatment of withdrawal cases

The researcher must fill in the reason of withdrawal in the case report form, contact the patient as much as possible, complete the evaluation project that can be completed, fill in the follow-up record form at the end of treatment, and record the time of the last administration as far as possible. For those who withdraw due to adverse events and are finally judged to be related to the treatment after follow-up, they must be recorded in the case report form and notified to the sponsor.

All study-related toxicities and SAE that exist at the time of withdrawal must be followed up till remission unless requested by the researcher. The condition cannot be alleviated because of the patient's disease itself.

After the patient terminated the study, the researchers tried to track all existing or new AE that occurred within 28 days after the last infusion of humanized meplazumab. And report all new AE and SAE occurring within this period of time. It must be reported to the sponsor within 24 hours if it is an SAE and follow up until the adverse events mentioned above are alleviated. After the patient terminates the study, the researcher needs to notify the sponsor immediately. Any SAE needs to contact the sponsor in time with the corresponding SAE reporting procedure.

12.3 Case report form (CRF)

All filling out, modification, and replacement of CRF must be carried out by the investigator or its authorized personnel. Queries will be generated by the EDC system. The investigator or its authorized personnel shall answer or correct the questions raised.

12.4 Record retention

In accordance with the International Conference on Harmonisation- Good Clinical Practice (ICH-GCP) guidelines, the investigator/research unit is required to maintain the original file of all CRF, data collected from each subject, and all study documentation required under ICH-GCP section 8 and the current regulations. Measures should be taken to prevent accidental or premature destruction of these documents.

13. Statistical analysis

13.1 Statistics and analysis scheme

Data on all safety, tolerability, and antiviral effects for each dose group created the table and summarized according to the statistical analysis plan. Generally, the data are summarized, used descriptive statistics (number of subjects, mean, median, standard deviation, minimum and maximum) to summarize the continuous variables, frequency and percentage were used to summarize the type variables. The data will be presented by dose group.

13.2 Analysis data set

Full analysis set (FAS): Defined as subjects who were successfully enrolled and treated

with humanized meplazumab injection.

Security analysis set: Defined as subjects who received humanized meplazumab injection and collected safety data after at least one treatment.

DLT Evaluation Analysis set: Defined as subjects who observed DLT during the study period or who did not observe DLT but completed 2-3 drug transfusions and underwent safety assessment 28±3 days after the first transfusion.

Efficacy evaluable set: Defined as subjects who had been evaluated for efficacy after at least one treatment in the full analysis set.

13.3 General principles of statistical analysis

SAS9.4 or later was used for statistical analysis.

The study baseline was defined as the results of the last non-missing test before the first humanized infusion of meplazumab injection.

The continuous variables will use the example number (non-missing number), mean, standard deviation, median, maximum and minimum.

Statistical description. Calculate the number and percentage of each category of the categorical variable. Unless otherwise stated, the number of missing cases will not be included in the percentage calculation. Demographic data and baseline indicators will be analyzed in the FAS population. All demographic variables and baseline characteristics (sex, birth date, weight, disease category, combined medication, previous treatment history, vital signs, etc.) were summarized by dose group.

13.4 Curative effect analysis

The changes of each observation index before and after each treatment will be calculated, statistics and its bilateral accurate 95% confidence interval (Clopper-Pearson confidence interval), and the number and percentage of cases under each result classification will be calculated at the same time.

13.5 Safety analysis

The incidence of DLT and bilateral accurate 95% confidence interval (Clopper-Pearson confidence interval) were calculated.

Adverse events during treatment will be summarized and analyzed. Adverse events during treatment were defined as those that occurred after the first treatment with humanized meplazumab injection. Adverse events occurring during treatment will be summarized according to the organ system and preferred terminology, and the number of cases and incidence rates under each category will be calculated.

Descriptive statistics were used to summarize the safety evaluation parameters such as laboratory examination, vital signs, electrocardiogram, and so on. The changes of each parameter relative to the baseline were summarized, and the number and percentage of cases under each category were calculated according to the classification of normal and abnormal

(with or without clinical significance).

13.6 Interim analysis

There is no interim analysis plan.

13.7 Determine the sample size

In this study, 10-20 patients were enrolled in a single-dose group.

14 Adverse event report

Timely, accurate, and complete reporting and analysis of safety information from clinical studies are critical to protecting subjects, researchers, and sponsors, it is also a mandatory requirement of the pharmaceutical administration. The sponsor has established standard operating procedures (SOPs) in accordance with the requirements of the quality management code for clinical trials of drugs (Order No. 3 of the Ministry of Health), the administrative measures for adverse drug reactions reporting and monitoring (Order No. 81 of the Ministry of Health), and the administrative measures for drug registration (Order No. 28 of the Ministry of Health), so as to ensure the proper reporting of safety information.

All clinical studies initiated by sponsors are required to report adverse events in accordance with this SOPs.

14.1 Related definition

14.1.1 Pre-treatment event

Pre-treatment event (PTE) clinical study subjects had signed informed consent to participate in the study, but any adverse medical event that occurred prior to the administration of any study drug; this event does not necessarily have a causal relationship with the participating study.

14.1.2 Definition and classification of adverse events

• Adverse Events

Adverse events (AE): any adverse medical events that occur to subjects after signing the informed consent form in clinical trials, do not necessarily have a causal relationship with research drugs or research procedures. As a result, adverse events can be any adverse or undesirable symptoms, signs, or diseases, including adverse drug reactions, important laboratory outliers, and diseases that occur during the study. Researchers must report all adverse events in the electronic case report form (CRF). All adverse events that occurred during the study should be recorded in the CRF table.

• Serious adverse events

A serious adverse event is an adverse event that occurs at any stage of the study (i.e. screening, treatment, follow-up), during the use of any dose of research drug, control drug or placebo and meets one or more of the following criteria:

(1) Lethal;

(2) Life-threatening;

(3) Causing prolonged hospitalization or hospitalization;

(4) Leading to significant or permanent disability/loss of function;

(5) Leading to congenital abnormalities or birth defects;

(6) Serious medical events: for example, important medical events that do not immediately endanger life, or death, or require hospitalization but endanger the patient, or require medical intervention to prevent the above outcome, it is necessary to make a scientific medical judgment immediately and decide whether to report quickly. These should also be considered serious adverse events.

Further explanations for serious adverse events are as follows:

(1) Deaths caused by any adverse events occurring within 28 days after the last administration, or the last visit. If the subject died during the study and an autopsy was performed, the autopsy results should be included in the subject's CRF.

(2) The occurrence of adverse events immediately brings the risk of death to the subjects. It does not include adverse events that may lead to death after serious progress, such as drug-induced hepatitis without liver failure.

(3) Any adverse events that lead to hospitalization and extended hospitalization (extended hospitalization refers to the delayed planned or expected discharge date, usually overnight in the hospital for at least one day). The selective surgery or admission examination decided before the trial was not included, and the treatment process did not change during the study.

(4) Any adverse event that results in injury, damage or destruction of thesubject's function, physiological structure, or both, physical activity or quality of life.

(5) It is suspected that the exposure of either parent to the treatment will lead to adverse results for future generations.

• Important adverse events

Major adverse events: refers to any adverse events, other than serious adverse events, that lead to the use of targeted medical measures (such as drug withdrawal, dose reduction and symptomatic treatment) and significant abnormalities in hematology or other laboratory tests.

• Adverse reaction

A new pharmaceutical product or new usage, during clinical use prior to approval, especially during the period when the treatment dose is not yet established, harmful or undesired reactions at any dose that have a causal relationship to drug use should be considered for adverse drug reactions (ADRs).

14.2 Other points to consider for PTE and AE

Adverse outcomes may usually be:

(1) Indicate a new diagnosis or unexpected deterioration of an existing disease. Sporadic events caused by existing underlying diseases should not be regarded as PTE or AE;

(2) Need treatment intervention;

(3) Need invasive diagnostic procedures;

(4) Need to discontinue or change the dose of research drugs or concomitant drugs;

(5) Researchers regard it as an adverse outcome for any reason;

(6) PTE/AE caused by research procedures (for example, post-collection bruising) should be recorded as PTE/AE. Compare the signs and symptoms before and after the study procedure:

(7) Each event should be recorded by a single diagnosis. Concomitant signs (including abnormal laboratory values or ECG results) or symptoms should not be recorded as additional AE. If the diagnosis is unknown, the signs or symptoms can be recorded as PTE or AE accordingly.

Laboratory values and ECG results:

(1) Changes in laboratory values or ECG parameters belong to PTE or AE only if they are determined to be of clinical significance (that is, if some measures or interventions are required, or if the researchers conclude that the changes are beyond the normal range of physiological fluctuations). Laboratory retesting and/or continuous monitoring of outliers is not regarded as an intervention. In addition, repeated or additional non-invasive tests used to verify, evaluate or monitor an anomaly are not considered intervention.

(2) If the abnormal laboratory test value or ECG result is the pathological result of an overall diagnosis (for example, elevated creatinine in renal failure), only the diagnosis should be reported as PTE or AE accordingly.

Existing diseases:

(1)A surviving illness (which exists at the time of the signing of informed consent) is considered an accompanying illness and should not be recorded as PTE or AE. Baseline evaluations (e.g., laboratory inspections, ECG, X-rays, etc.) should not be recorded unless relevant to the operation of the study recorded as PTE. However, if the subject's accompanying condition worsens or develops complications, the deterioration or complications should be recorded as PTE (deterioration or complications occur before the study drug begins to be given) or AE (Deterioration or complications occur after the study of the drug has begun to be given). Researchers should ensure that the documented event terminology can reflect changes in the condition (e.g., "the deterioration of...").

(2)If the subject has a pre-existing disease (e.g., asthma, epilepsy), only seizures become more frequent, more serious, or aggravating, the seizure should be recorded as PTE/AE, i.e., the researcher should ensure that the record AE terms can describe changes in the relative baseline of the condition (e.g., "the deterioration of...").

(3)If the subject has accompanying degenerative conditions (e.g., cataracts, rheumatoid arthritis), the deterioration of the disease should be recorded as PTE/AE only if the deterioration of the disease exceeds expectations. In addition, the researcher should ensure that the recorded AE terms describe changes in the condition (e.g., "the deterioration of...").

Deterioration of PTE or AE:

(1) If the subject develops a deterioration or complication of PTE after the study of the drug administration, the deterioration or complications should be recorded as AE. The researchers should ensure that the recorded AE terms describe changes in the condition (e.g., "the deterioration of...").

(2) If the subject develops a deterioration or complication of AE after any change in the drug, the deterioration or complication should be recorded as a new AE. The researcher should ensure that the recorded AE terms describe changes in the condition (e.g., "the deterioration of...").

Changes in AE/PTE severity:

If the subject has a change in AE/PTE severity, the event should be recorded once at its maximum severity.

Pre-planned surgery or operation:

(1) Pre-planned operations (surgery or treatment) arranged prior to signing informed consent are not considered PTE or AE. However, if pre-planned operations are carried out ahead of time due to the deterioration of the existing condition (e.g., as an emergency), the deterioration of the condition should be recorded as PTE or AE. Complications from any planned surgery should be reported as adverse events.

(2) Elective surgery or operation: The elective operation performed without any change in the subject's condition should not be reported as PTE or AE but should be recorded in the subject's source file. Complications due to elective surgery should be reported as adverse events.

(3) Inadequate clinical response (lack of effectiveness): Inadequate clinical response, effectiveness, or pharmacological action should not be recorded as AE. The lead researchers must distinguish between the worsening of existing diseases and the lack of treatment effectiveness.

14.3 Definition of causation

The causal relationship between the drug and adverse events is determined according to the following criteria.

(1)Not related: Adverse events are not related to the study drug; the timing of the reaction does not conform to the chronological order of the drug, the reaction is consistent with the known type of reaction of the non-experimental drug, the patient's clinical status, or other treatment may also produce the reaction, the disease state improves, or the other treatment is discontinued, Re-use of other treatment synods appears and is closely related to other risk factors.

(2)Doubtful: Adverse events are more likely to be explained by other explanations; the timing of the reaction does not conform to the chronological order of the drug, the reaction is not quite in line with the type of reaction known in the experimental drug, and the patient's clinical status or other treatment methods may also produce the reaction. The possibility of medication-related cannot be ruled out.

(3)Possible: Adverse drug reactions may be associated with the study drugs. Other explanations are not convincing; the timing of the reaction is consistent with the

chronological order of the drug, the reaction conforms to the type of reaction known in the experimental drug, and the patient's clinical status or other treatment methods may also produce the reaction

(4)Probable: Adverse events may be related to the study drugs, correlations may be possible of time, and other explanations are unlikely

(5)Very likely: Adverse events have been classified as possible adverse reactions, and there is no reason to use other explanations. The time of reaction appears in accordance with the chronological order of the drug, the reaction conforms to the known type of reaction of the experimental drug, the reduction or stop of the drug is improved, and the repeated administration occurs again.

(6)It is not possible to determine: The timing of the reaction is not clearly related to the timing of the drug use, the reaction is similar to the type of reaction known in the experimental drug, and other drugs used may also cause the same reaction, there is not enough evidence to judge.

14.4 Criteria for judging the severity

Assess the severity of AE according to CTCAE v5.0.

Grade 1 Mild; asymptomatic or mild symptoms; clinical or diagnostic observations only; intervention not indicated.

Grade 2 Moderate; minimal, local or noninvasive intervention indicated; limiting age-appropriate instrumental ADL*.

Grade 3 Severe or medically significant, but not immediately life-threatening; hospitalization or prolongation of hospitalization indicated; disabling; limiting self-care ADL**.

Grade 4 Life-threatening consequences; urgent intervention indicated.

Grade 5 Death related to AE.

14.5 The measure related to the investigational product

(1) Drug withdrawal: Experimental drug discontinuation due to specific AE.

(2) Dose remains unchanged: No need to discontinue experimental drugs due to specific AE.

(3) Unknown: Only when the measure is taken cannot be determined.

(4) Inapplicability: Experimental drugs discontinuation due to the other reason than specific AE, for example, termination of the study, death of the patient, discontinuation of the investigational product prior to the occurrence of the AE.

(5) Dose reduction: Decreasing the dose due to specific AE.

(6) Dose interruption: Experimental drugs are temporarily interrupted (suspend) (including the patients interrupt proactively) due to specific AE, and drugs are resumed later.

14.6 Outcome of AE

(1) Recovered/resolved: "The end date of (serious) adverse event "shall be indicated.

(2) Recovering/resolving: The event is not completely resolved, but the patient is recovering.

A follow-up is needed.

(3) Unrecovered/unresolved: The event is in progress.

(4) Recovered/resolved, with sequelae: Only when the patients have long-lasting or lifelong sequelae, such as blindness caused by diabetes, hemiplegia caused by stroke. “The end date of (serious) adverse event” shall be indicated.

(5) Fatal: “The end date of (serious) adverse event” shall be indicated. The time of death shall be recorded when the patient is dead due to the AE.

(6) Unknown: The researchers are unable to understand the AE. For example, the patient lost the follow-up.

If the outcome of the AE is rated as “recovering/resolving”, or “not recovered / unresolved,” or “unknown,” the end date of AE may not be recorded temporarily.

If the outcome of the AE is rated as “recovered/resolved” or “recovered/resolved, with sequelae,” the end date of AE must be recorded.

All the AE must be followed up in order to determine the final outcome or to achieve a stable state.

When the patient completes the clinical research, the researcher shall follow up the outcome of AE that may be related to the experimental drug or cannot be determined or until it achieves a stable state.

14.7 Special report

The AE that the particular sponsor concern requires a rapid reporting process. Such events include but are not limited to:

(1) Excessive use of drugs due to wrong calculation or re-infusion;

(2) Severe transfusion reaction;

(3) The early termination caused by AE.

The event of a special report should be recorded in CRF. If such events conform to the standards of SAE, *the SAE report form* shall be completed.

14.8 Collection and reporting procedures

14.8.1 Procedure for collection and reporting of pre-treatment events and all adverse events

Collection period.

PTE collection will begin with the patient signing the informed consent form to participate in the research and continue until the patient receives experimental drugs for the first time or the screening fails. PTE will be collected for the patient who stopped before experimental drugs were infused back to the research.

AE will be collected from the time when the patient first received experimental drugs to the tracking period of 28 days after the last administration of the patient, no less than 28 days. Only spontaneous reports will be collected when they exceeds the tracking period.

The end date of AE / PTE is the date when the patient recovers, the event subsides but has sequelae or the patient dies.

Report

At each research visit, the researchers will assess whether subjective AE occurred. A neutral question can be asked, such as “How have you felt since your last visit?”. Patients can report AE at any other time during the research. Patients with PTE must be monitored until symptoms subside or any clinically relevant changes in laboratory tests have returned to baseline, or the changes are satisfactorily explained. Non-severe PTE related to or unrelated to the research procedure does not need to be followed up according to the purpose of the research protocol. AE in all patients, whether or not related to the use of the experimental drug, must be monitored until symptoms subside and any clinically relevant changes in laboratory test values have returned to baseline, or the observed changes are satisfactorily explained. All clinically significant laboratory abnormalities confirmed by laboratory retests will be followed up until they return to acceptable levels or are satisfactorily explained. All PTEs and AEs will be recorded on the PTE / AE page of the CRF, regardless of whether the researcher concludes that the event is related to the treatment of the experimental drug. The following information

will be recorded for each event:

(1) Event terminology;

(2) The times and dates of start and end;

(3) Severity;Researcher’ judgment on the causal relationship between the event and the administration of the experimental drug (relevant or irrelevant) (PTE is not required);Researcher’ judgment on the causal relationship between the event and the research procedure, including details of suspicious procedures;

(4) The measure related to the experimental drug (not applicable to PTE);

(5) The outcome of the event;

(6) Seriousness.Patients’ diaries and questionnaires will not be the main means to collect AE. However, if researchers find potential AE through the information collected in these documents, patients should be followed up appropriately for medical evaluation. Through this follow-up, if it is determined that AE not reported before is found, it should be reported according to the normal reporting requirements.

14.8.2 Collection and reporting of serious adverse events

Any serious adverse event occurred in the trial, whether related to the test drug or not, the investigator should give timely rescue treatment, Fill in the “Significant Adverse Event (SAE) Report Form” of the National Drug Administration (NMPA) within 24 hours of being informed, sign and date, And immediately report it to the sponsor (or CRO appointed by the sponsor) by fax, the ethics committee of the research center where it is located, the National Drug Administration (NMPA), the provincial and municipal local drug supervision and administration, and the national health and health committee Board. SAE should keep a detailed record of the description of the symptoms, the severity, the time of occurrence, the time of treatment, the measures taken, the time and manner of follow-up, and the outcome.

Researchers must provide their assessment of causality when reporting serious adverse events. If the investigator’s assessment of causality is lost or unavailable, the sponsor’s judgment will be used until the investigator’s assessment can finally be obtained.

If the investigator cannot determine whether an adverse event is a serious adverse event, it is considered a serious adverse event until the nature of the event is proven. Such incidents need to be notified in writing to local authorities and researchers in accordance with local requirements. For all serious adverse events (including those that are still in development after the end of the study and within 28 days of the end of the study), the investigator needs to follow up until there are clear results to ensure that all issues are resolved. Provide detailed follow-up information (e.g., whether special treatment is needed after the study, whether hospitalization is required, etc.). The investigator submits a follow-up report to the CRO until the adverse event resolves. In the case of permanent damage, follow up until the adverse event is considered stable.

14.9 Death

All deaths that occurred during the experiment or within 28 days after exiting the trial (last visit) must be notified to the sponsor within 24 hours, notified to the regulator within 7 days, and reported relevant within the next 8 days (15 days in total) Follow-up (cause of death, autopsy report, and hospital report) information.

If the experiment is withdrawn from death, the event is reported as disease progression or adverse event, and the cause of death is recorded at CRF. If death comes from disease progression and other causes, the investigator must determine the main cause of death and appropriately classify the reasons for withdrawal.

14.10 Hospitalized

Adverse events requiring hospitalization are considered serious adverse events. Generally, admission is for admission procedures and treatment, and this adverse event should be considered a serious adverse event.

Because of elective surgery, routine clinical procedures, annual inspections, admission observations or protocols, not due to adverse events. If an unexpected event occurs during this process, it should be reported as a “serious” or “non-serious” adverse event according to conventional criteria.

Note: Hospitalization or extension of hospital stay for non-medical reasons/facility reasons or purely for clinical trial purposes does not meet the criteria for a medical event and cannot be considered a serious adverse event.

14.11 Pregnancy

All pregnancies must be reported, and the investigator reports to the sponsor using the preliminary pregnancy report form within 30 days of becoming aware of the pregnancy. The investigator must follow up and record all pregnancy processes and outcomes, even if the subject has dropped out of the study or the study has ended.

All pregnancy outcomes must be reported by the investigator to the sponsor using the pregnancy outcome report form within 30 days of being informed of normal or selective abortion.

Pregnancy alone is not considered an AE unless it is suspected that the experiment medicine may affect the effectiveness of the contraceptive; selective abortion without complications should not be treated as AE unless it is a therapeutic abortion. Hospitalization due to normal delivery of a healthy newborn should not be considered SAE.

Any SAE that occurs during pregnancy (including SAE that occurred after the last dose of study drug) must be recorded on the SAE report form (e.g., severe maternal complications, spontaneous or therapeutic abortion, ectopic pregnancy, stillbirth, newborn Infant deaths, congenital abnormalities, or birth defects) and report within 24 hours following the SAE reporting process.

14.12 Overdose

Exceeding the therapeutic dose of the regimen prescribed in the protocol should be recorded as an overdose on the CRF, and the presence of any AEs associated with an overdose should be recorded. AEs related to drug overdose should be given symptomatic supportive treatment.

15 Research Drug Information

15.1 The name and specifications of the treatment

Humanized Meplazumab for injection is a new humanized IgG2 monoclonal antibody developed by Jiangsu Pacific Menok Biopharmaceutical Co., Ltd. (hereinafter referred to as Pacific Menok) and The Fourth Military Medical University. Meplazumab for injection is a humanized IgG2 antibody recombinantly expressed in CHO cells (CHO DG44, a mutant Chinese hamster ovary cell). The molecule consists of 2 heavy chains containing 442 amino

acid and 2 Light chains containing 214 amino acids are linked by disulfide bonds, each light chain contains 2 intrachain disulfide bonds, and each heavy chain contains 4 intrachain disulfide bonds; the heavy-to-heavy chain contains 4 Interchain disulfide bonds, the light chain-heavy chain contains 2 interchain disulfide bonds, the complete protein contains a total of 18 disulfide bonds. The asparagine at position 292 of the heavy chain Fc region of the molecule is the sole glycosylation site of the molecule, and the glycosylation modifications are mainly GOF, G1Fa, G1Fb and G2F glycoforms. The theoretical amino acid molecular weight of this molecule is 144094Da and a theoretical isoelectric point of 7.45. The affinity constant (KD) of Meplazumabl for injection and the CD147 molecule was 1.7 × 10^{-10} M. The molecular formula is $C_{6406}H_{9866}N_{1692}O_{2009}S_{50}$.

Biological Characteristics

1) Antibody humanization

We used bioinformatics, DNA recombination technology and other means to amplify the light and heavy chain variable region genes of antibodies from hybridoma cell lines expressing anti-CD147 antibody 6H8 and used bioinformatics methods and recombination technology to replace the FR sequences in the variable regions of the antibody light and heavy chains with human-derived FR sequences and insert them into expression vectors containing the human IgG2 antibody constant region gene, and transfect the corresponding host cell expression produce.

The CDR regions of the light and heavy chains of the expressed antibody molecule are of mouse origin, while the FR region and constant region are of human origin, and more than 2/3 of the entire molecule are of human origin. Among them, the variable region has the function of binding antigen, and the constant region has Antibody effector function, immunogenicity, and species characteristics.The Fc segment of the chimeric antibody can prolong the half-life of the antibody in the serum and theoretically reduce the immunogenicity of the heterologous antibody. This technology retains the complete murine monoclonal antibody variable region sequence, ensuring antibody affinity and specificity. Although theoretically, the constructed chimeric antibody still retains the heterogeneity of the murine variable region and may induce a HAMA response, animal experiments have shown that humanized Meplazumab injections have no Cause abnormal clinical symptoms in test animals, autonomic activity, weight, food

intake, body temperature, blood pressure, electrocardiogram, hematology index, serum biochemical index, urine index, CD3 + / CD4 +, CD3 + / CD8 + lymphocytes, bone marrow cells, histopathology and injection site were no significant effects.

2) High affinity

The strength of the antibody-antigen interaction is mainly determined by the affinity between them. These interactions are caused by non-covalent bonding. Because some amino acid sites of the parental non-human monoclonal antibody framework region may be involved in antigen binding or have an important role in maintaining the conformation of the antigen-binding region, therefore, the affinity of a humanized antibody will be reduced to a certain extent compared with its parental non-human monoclonal antibody. However, the affinity of humanized Meplazumab injection to the extracellular molecule of CD147 was measured by the SPR system. Analysis using the Kinetic-Langmuir model showed that humanized Meplazumab injection Compared with the parental non-human monoclonal antibody 6H8, the equilibrium dissociation constant did not change significantly. The affinity constant (KD) was 1.7 × 10-10M, indicating that humanized Meplazumab injection has a similar affinity to its parental mouse monoclonal 6H8 (affinity constant KD = 4.48 × 10-10M), which guarantees the ability of the monoclonal antibody to bind to the target molecule in vivo and in vitro.

15.2 Formulation

Based on the results of the formulation study, the final formulation of Meplazumab for injection is shown in the table below.

Table 1 Final formulation of humanized Meplazumab injection for injection (100 units)

Component name	Prescribed amount
Meplazumab	10.00 g
Histidine	1.60 g
Histidine Hydrochloride	3.08 g
Mannitol	70.0 g
Sucrose	50.0 g
Polysorbate 80	1.00 g
Water for Injection	Add to 1000 mL

15.3 Drug management

15.3.1 Transportation and storage

The monoclonal antibody drugs are received, treated, and stored by the specific person in the research center. The drug should be kept under the specified conditions before administration. Only the person in charge of the research can enter and verify it and record it in the CRF. The investigator is responsible for returning all unused or partially used treatments to the sponsor.

15.3.2 Waste treatment

Disused antibody drugs (including mislabeled, underused, rejected, etc.) should be collected and dealt with in a centralized manner. All supplies used for transfusion should be collected, including infusion tubes, etc. The collected antibody drugs and infusion wastes should be kept by a specific person and registered timely. Meanwhile, they should be disposed of within one week according to the standard of medical waste.

16. Ethics

16.1 Responsibility of the investigator

The investigator is responsible for ensuring that the clinical study is carried out in accordance with the protocol, the current ICH-GCP, and the relevant regulations of the national drug administration of China (NMPA). The ICH-GCP is an internationally recognized ethical and scientific quality standard for the design, operating, recording, and reporting of research on the human body. Studies that comply with this standard are consistent with the principles set forth in the declaration of Helsinki in terms of protecting the rights and interests of subjects and to be reliable in terms of the quality of research data.

16.2 Independent ethics cast (IEC) /institutional review board (IRB)

Prior to study initiation, the investigator shall provide IEC/IRB with the following documents: investigator's manual, study protocol, CRF, informed consent, etc.

Only after IEC/IRB has given its full consent to the study protocol, the informed consent, the materials to assist subject inclusion, the compensation measures for subjects, and the sponsor has received a copy of the IEC/IRB approval document can the trial begin. The approval document shall indicate the approved research topic (program number), the name of the research document (including version number), and the approval date.

At the end of the study, the investigator shall notify the IEC/IRB that the test has been

Completed.

16.3 Informed consent

It is the investigator's responsibility to explain to each subject the purpose, methodology, benefits, and potential risks of the clinical trial. Prior to any procedure related to a clinical trial, informed consent must be obtained from the subject. Informed consent shall be expressed both orally and in writing. Informed consent must be dated and signed by the subject, and for those subjects who for any reason cannot sign the informed consent themselves, it must be signed by their parent, legal guardian, or protector. Copies of the signed informed consent and information sheet will be kept by the subject.

The informed consent must be approved by both the sponsor and IEC/IRB. The informed consent should comply with the Helsinki Declaration, the current GCP guidelines, the applicable regulations and the requirements of the sponsor.

By signing the informed consent, the subject/patient must also agree to allow the sponsor, the FDA, the auditor, and the inspector to verify the raw data obtained regarding the clinical study, and the reviewer must comply with the confidentiality statement.

16.4 Protection of subject data

This study only collected and processed data from subjects who were essential to the conduct of the study of the efficacy, safety, quality, and application of drugs.

The data will be collected and used to ensure its confidentiality and to comply with relevant laws and regulations protecting the subject's privacy.

The investigator must take appropriate technical steps and regulatory measures to protect the subject's personal information from unauthorized access and disclosure, accidental and illegal destruction, and accidental loss and alteration. The sponsor who has access to the subject's personal information will keep it confidential throughout the study period.

16.5 Data monitoring committee (DMC)

An independent data monitoring committee should be established by the sponsor. The committee is composed of experts from the areas of infectious diseases, respiratory medicine, cardiovascular medicine, neurology, biostatistics, and medical ethics.

Responsible for assessing safety and tolerability and DLT judgment in the dose-increasing phase and deciding whether to enter the next dose group or stop the test according to the judgment of result.

17. Management requirements

17.1 Modify the program

All changes to the scheme should be signed and dated by the sponsor and then issued. It cannot be implemented without IEC/IRB approval, except to avoid the subject's current risk or to make only logistical or administrative changes to the study (such as misprints, inconsistencies).

17.2 Data management

17.2.1 Completion and transfer of raw data and case report form (CRF)

The CRO data division is responsible for data management in this study to ensure the authenticity, integrity, privacy, and traceability of clinical trial data.

The data in the electronic case report form are all from the original medical records, which are filled in by the researcher or the designated personnel of the researcher. The completeness and accuracy of the information should be ensured. If there are any errors that need to be corrected, the modification shall be conducted according to the CRF instructions. The CRF system will automatically record the name and modification date of the data modifier.

After the completion of the CRF, the data in the CRF system should be submitted to the CRF system through the network in a timely manner. After the data in the CRF system is confirmed by SDV, DM review, and questioning, the researcher needs to confirm the electronic signature before the data is locked.

17.2.2 Design and establishment of a database

The database was established by the CRO data department and should meet the requirements of the biostatistical guidelines for drug clinical trials. The database needs to manage data traces such as system login, data entry, modification, or deletion, and the database should be established according to CDISC standards whenever possible.

17.2.3 Data entry

The data are entered into the EDC database by authorized personnel. After the data entry is completed, the EDC system will be checked by programming research specific logic checks to ensure the completeness and accuracy of the information.

17.2.4 Query handling

After the data is entered and stored in the EDC system, the audit system is activated and raises questions that need to be reviewed and answered by the researcher. The data manager will review the researcher's answer and close the query if the answer is acceptable. The data manager also manually checks the input data to ensure its logicality, consistency, and accuracy. The subject data list/report was generated programmatically to support manual data verification throughout the study. When the data needs to be clarified/verified/confirmed by the researcher, a manual query can be added to the EDC system. The data administrator needs to confirm that all queries have been resolved before locking the repository.

17.2.5 Data quality assurance

The clinical study was reviewed for quality assurance by the sponsor or a person authorized by the sponsor. GCP reviews can also be conducted by the drug approval department. The quality auditor has access to all medical records, documents and letters related to the study, and the informed consent document for the clinical trial.

17.3 Inspection

The sponsor shall designate inspectors to conduct on-site supervision. The supervisor is from the sponsor or CRO company authorized by the sponsor and shall operate according to the company's SOP. Inspectors shall visit periodically from the beginning to the end of the study.

Inspectors may access the relevant raw data for this clinical study and review the CRF in accordance with the SOP to determine the completeness, accuracy and consistency of the information with the original data.

Copies of CRF, laboratory data, and medical test results must be readily available to clinical supervisors, auditors, and health authorities. Inspectors are required to review all CRF and informed consent.

17.4 Audit and Inspection

Comply with GCP and the sponsor audit plan. The sponsor representative may choose to audit this test so as to evaluate the execution and solutions of the test, GCP compliance, and related management regulations. Besides the center's facilities (such as storage location of the drug and laboratory, etc.) and test records will be inspected.

Government regulators may also have to inspect the sponsor and/or research center facilities. After receiving notification on the inspection of the research center, the sponsor needs to immediately inform the relevant researchers. Also, the researchers should inform the sponsor of any of the upcoming inspection.

Representatives of government regulators and the person in charge of the audit must have access to the following practices:

- Inspect the test center facilities;
- Meet all team members related to the test;

- Check the test data and the original file directly;
- Consult all the other documents related to the test.

The audit and inspection may be carried out in the process of the test or any time after the test to ensure test effectiveness and data integrity.

17.5 Original Records for Verification

Researchers must properly deal with all the data obtained from the clinical research process to protect the rights and privacy of all participants in the clinical test. Researchers must allow the examiners/auditors/inspectors to check and review the clinical research data needed to verify the accuracy of the original data and understand the progress of research. If the original records can't be verified, the researchers shall assist the examiners/auditors/inspectors with further confirmation on the quality control of the data.

17.6 The End/Termination of the Research

The end of the Research

Only after the completion of the last participant's last visit and the ORU's notification to the sponsor should be viewed as to be ended, the sponsor will notify the end time of the study to all ORUs. After this, any continuation of the test must be approved by the sponsor, in which case the test can be implemented without a supplement plan.

The Termination of the Research

The sponsor reserves the right to terminate the test unit testing at any time. Reasons for the sponsor to terminate or stop the test at a research center may include, but not limited to:

(1) Researchers do not comply with the test plan or GCP guidelines;

(2) Security concerns;

(3) There is enough evidence to suggest the lack of efficacy;

(4) The researchers can’t recruit enough subjects for the test.

17.7 Confidentiality Agreement and Patient’s Privacy

Researchers need to make the commitment that any confidential information obtained from the sponsor or provided or disclosed through the contractual relationship shall be kept from the third party, and all information can only be used within the range which has been speculated in the agreement.

The confidentiality agreement is independent and valid for the duration of the contractual relationship between the two parties as long as the sponsor has reasonable and legitimate reasons to require the researchers to maintain the agreement.

Researchers must ensure the protection of clinical trial subjects’ privacy. In all the documents submitted to the sponsor, the status of clinical trial subjects is certified only by the code of clinical trial subjects instead of the name and the admission number. Researchers must properly keep the relevant clinical trial participants’ name, address and the tables corresponding to the clinical research subjects’ code. Researchers should strictly keep all of these table confidential, to which the sponsor shall have no access.

17.8 The Use and Publication of the Information

The Second Affiliated Hospital of Infectious Department, National Translational Science Center for Molecular Medicine, as the sponsor, has the exclusive right for this study. Author and the text will reflect the cooperation of a number of researchers, ORUs and staffs from the sponsor. The author should be determined before the writing. Since many researcher units participated, no individual shall be allowed to write articles and publish in the name of his own unless otherwise with the consent of the sponsor. The sponsor has the final say with regard to the articles and publications.

18. References

[1] Landras, A, C Reger de Moura, F Jouenne, et al., CD147 Is a Promising Target of Tumor Progression and a Prognostic Biomarker [J]. Cancers (Basel), 2019, 11(11).

[2] Vanarsdall, AL, SR Pritchard, TW Wisner, et al., CD147 Promotes Entry of Pentamer-Expressing Human Cytomegalovirus into Epithelial and Endothelial Cells [J]. mBio, 2018, 9(3).

[3] Pushkarsky, T, G Zybarth, L Dubrovsky, et al., CD147 facilitates HIV-1 infection by interacting with virus-associated cyclophilin A [J]. Proc Natl Acad Sci U S A, 2001, 98(11): p. 6360-5.

[4] Watanabe, A, M Yoneda, F Ikeda, et al., CD147/EMMPRIN acts as a functional entry receptor for measles virus on epithelial cells [J]. J Virol, 2010, 84(9): p. 4183-93.

[5] Chen, Z, L Mi, J Xu, et al., Function of HAb18G/CD147 in invasion of host cells by severe acute respiratory syndrome coronavirus [J]. J Infect Dis, 2005, 191(5): p. 755-60.

[6] Zhang, MY, Y Zhang, XD Wu, et al., Disrupting CD147-RAP2 interaction abrogates erythrocyte invasion by Plasmodium falciparum [J]. Blood, 2018, 131(10): p. 1111-1121.

[7] Bian, H, JS Zheng, G Nan, et al., Randomized trial of [131I] metuximab in treatment of hepatocellular carcinoma after percutaneous radiofrequency ablation [J]. J Natl Cancer Inst, 2014, 106(9).

[8] Wu, L, YF Yang, NJ Ge, et al., Hepatic artery injection of (1)(3)(1)I-labelled metuximab combined with chemoembolization for intermediate hepatocellular carcinoma: a prospective nonrandomized study [J]. Eur J Nucl Med Mol Imaging, 2012, 39(8): p. 1306-15.

[9] Ma, J,JH Wang, 131I-Labeled-Metuximab Plus Transarterial Chemoembolization in Combination Therapy for Unresectable Hepatocellular Carcinoma: Results from a Multicenter Phase IV Clinical Study [J]. Asian Pac J Cancer Prev, 2015, 16(17): p. 7441-7.

[10] Xu, J, ZY Shen, XG Chen, et al., A randomized controlled trial of Licartin for preventing hepatoma recurrence after liver transplantation [J]. Hepatology, 2007, 45(2): p. 269-76.

[11] Saphire, AC, MD Bobardt,PA Gallay, Human immunodeficiency virus type 1 hijacks host cyclophilin A for its attachment to target cells [J]. Immunol Res, 2000, 21(2-3): p. 211-7.

[12] Zhi-Nan Chen, an Antagonist Target CD147 Receptor of SARS-CoV and HIV-1, 2003, (CN1442203A)

[13] Zhi-Nan Chen, Use of HAb18G/CD147 Molecule as Target of Antiviral Antagonist and thus Obtained Antiviral Antagonist, (PCT/CN2003/00451)
[14] Zhi-Nan Chen, HAb18G/CD147, Its Antagonist and Application, (PCT/CN2002/000356)
